# Chemoenzymatic Total Synthesis of Lansai B

**DOI:** 10.1002/chem.202500740

**Published:** 2025-04-16

**Authors:** Marcel Schatton, Mona Haase, Julia Tenhaef, Catharina Gronkowsky, Stephan Noack, Jörg Pietruszka

**Affiliations:** ^1^ Institute for Bioorganic Chemistry & Bioeconomy Science Center (BioSC) Heinrich‐Heine‐University Düsseldorf im Forschungszentrum Jülich Jülich Germany; ^2^ Institute of Bio‐ and Geosciences (IBG‐1: Bioorganic Chemistry) & Bioeconomy Science Center (BioSC) Forschungszentrum Jülich Jülich Germany

**Keywords:** biocatalysis, late‐stage‐functionalization, methyltransferase, natural alkaloid, total synthesis, tryptophan synthase

## Abstract

Lansai B is an indole alkaloid whose biosynthesis is not yet elucidated. However, utilizing nature's tools enabled the stereoselective formation of this unique framework, first by providing the substituted tryptophan as starting material and later in the scaffold‐building step forming the pyrroloindole motif. For the latter, the C3‐methyltransferase from *Streptomyces griseoviridis* (SgMT) is employed for the diastereoselective methylation. Investigating the scope of the enzyme proved that introducing a bromide in the 5‐position was ideal for the synthetic endeavor. With this, the stage was set for the synthesis of lansai B, introducing the prenyl group late via a Suzuki coupling.

## Graphical abstract

Lansai B was obtained in a chemoenzymatic approach from the small building block 5‐bromoindole utilizing two enzymes. Applying a tryptophan synthase and a further characterized C3‐methyltransferase the sterically demanding framework is constructed within just six steps. After late‐stage Suzuki‐coupling and *n*‐methylation the natural product was isolated.

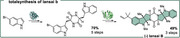

## 1 Introduction

**Scheme 1:**
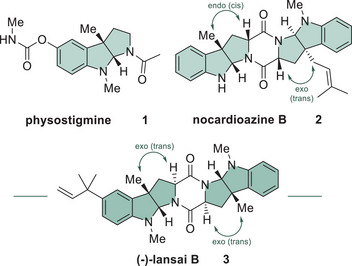
Structure of the natural products physostigmine (**1**), nocardioazine B (**2**), and lansai B (**3**) containing the HHPI motif (highlighted in green).

Within the hexahydropyrroloindole (HHPI) family, there are many different natural products with various biological activities.^[^ ^1^ ^]^ While physostigmine (**1**) is an inhibitor of the acetylcholinesterase,^[^ ^2^, ^3^ ^]^ nocardioazine A (**2**) is a noncytotoxic inhibitor of the p‐glycoprotein efflux pump (Scheme 1).^[^ ^4^ ^]^ Further studies have demonstrated the antibacterial and anticancer properties of these compounds.^[^ ^5^, ^6^ ^]^ In particular, lansai B (**3**) has been the subject of considerable interest due to its slight anticancer effect on BC (lymphoma) cancer cell lines. The compound was first isolated in 2008 from *Streptomyces* sp. *SUC1*.^[^ ^7^ ^]^ The semi‐rigid 2,5‐diketopiperazine (DKP) core of lansai B (**3**) has been noted as a common motif for natural products showing interesting physiological properties.^[^ ^8^, ^9^, ^10^, ^11^ ^]^ They exhibit a high capacity for forming hydrogen bonds, both as an acceptor and a donor, and display remarkable ability to interact with a diverse range of receptors.^[^ ^12^ ^]^ The formation of the DKP from tryptophan allows for the fusion of the HHPI ring system with the DKP core, thereby creating an even more rigid but unique three‐dimensional structure.^[^ ^7^, ^13^, ^14^ ^]^ Due to the (*S*)‐configurations of the adjacent stereogenic centers, the compound yields an arched structure.^[^ ^14^ ^]^

**Scheme 2:**
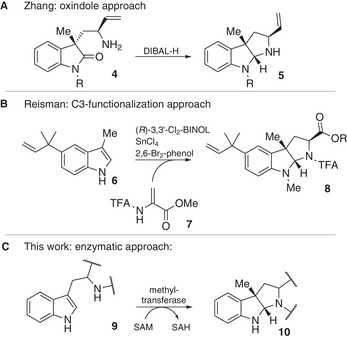
Approaches constructing HHPIs. (**A)** Oxindole approach applied by Zhang et al. in their synthesis of nocardioazine B. (**B)** C3‐functionalization approach applied by Reisman et al. in their synthesis of lansai B (**3**). (**C)** Enzymatic approach described in this publication.

However, the synthesis of HHPIs remains challenging with respect to the stereogenic centers that are required.^[^ ^15^, ^16^, ^17^ ^]^ There are two major approaches for the catalytic, asymmetric construction of the HHPI motif, starting from either 3,3‐disubstituted oxindoles (Scheme 2: A) or by domino C3‐functionalization of 3‐substituted indoles with simultaneous cyclization (Scheme 2: B).^[^ ^18^, ^19^, ^20^ ^]^

The latter was used from Reisman's group for the only published synthesis of lansai B (**3**), where (*R*)‐BINOL‐SnCl_4_ acts as a Lewis acid‐assisted Brønsted acid to add acrylate compound **7** to indole **6**.^[^ ^14^ ^]^ The diastereomeric ratio for this method was reported to range from 4:1 to 16:1, depending on the used BINOL derivative, with an enantiomeric excess of roughly 90%.^[^ ^21^ ^]^ In this manner, Reisman et al. prepared the unsubstituted and 5‐prenylated HHPI moiety (**8**). After removal of the protecting groups, the central DKP ring was finally formed from the two prepared HHPIs to yield lansai B (**3**). However, for the final step a yield of 38% was reported, which is due to the fact that not only the desired hetero‐DKP was formed but also the two homo‐products with either two unsubstituted or two prenylated HHPI moieties.^[^ ^14^ ^]^

In nature, the HPPI structural motif is formed using enzymes such as methyl or prenyltransferases through the consumption of the cosubstrate *S*‐adenosyl‐l‐methionine (SAM) or dimethylallylpyrophosphate as a methyl/prenyl source.^[^ ^22^, ^23^, ^24^ ^]^ In the case of the methyltransferase, a proposed reaction mechanism involves the activation via the indole nitrogen, increasing the nucleophilicity at C3 and thus facilitating the electrophilic methyl‐group transfer from SAM. The intermediate iminium ion is trapped by the intramolecular nucleophile, yielding the HHPI (see Figure S1).^[^ ^25^ ^]^ The specific orientation of SAM and the substrate within the enzyme pocket only allows for the production of a single diastereomer, which results in the formation of exo‐(trans)products when converting l,l‐DKPs.^[^ ^24^ ^]^

In previous studies, the activity of methyltransferases from *Streptomyces griseoviridis* ^[^ ^26^ ^]^ and *Streptomyces* sp. *HPH0547* ^[^ ^24^ ^]^ was tested against a broad range of different DKPs, consisting of tryptophan and other natural amino acids. Since lansai B (**3**) contains a prenyl group in the 5‐position, the scope was now extended to encompass non‐canonical amino acids. Halogen substituents were selected for investigation because of their reactivity in cross‐coupling reactions and, at the same time, their favorable size, which likely allows them to still fit into the enzyme pocket.

This study reports a new synthesis route of the natural compound lansai B (**3**), utilizing the methyltransferase from *Streptomyces griseoviridis* (SgMT). In contrast to Reismans’ total synthesis, the DKP ring is formed at an early stage, avoiding the unselective peptide formation.

## 2 Results and Discussion

### 2.1 Synthesis of Functionalized Diketopiperazines

In order to conduct preliminary experiments, functionalized DKP substrates were synthesized from racemic mixtures of 5‐brominated and chlorinated tryptophans due to their commercial availability (see Scheme S1). The l,l‐cyclic‐tryptophan‐tryptophan‐diketopiperazines (cWW‐DKP) were readily identified through a comparison of the ^1^H‐ and ^13^C‐spectra, as well as the optical rotation (see Table S1). This is due to the d,l‐diastereomers having a nearly symmetrical structure, which leads to overlapping NMR shifts and a very low optical rotation. As the methyltransferase exclusively converts l,l‐cWW‐DKP towards the double‐methylated product, there is no utility in the d,l‐cWW‐DKP. Thus, a much more efficient approach was the employment of a tryptophan synthase, as it gives access to halogenated tryptophans in an enantiomerically pure fashion.

Oelke et al. already described the valuable application of tryptophan synthases from *Salmonella enterica* for the total synthesis of chloptosin.^[^ ^27^, ^28^, ^29^ ^]^ Further tryptophan synthases from *Pyrococcus furiosus* and *Thermotoga maritima* have been extensively studied and bio‐engineered by the Arnold group, resulting in many different mutants of the native genes.^[^ ^30^, ^31^, ^32^ ^]^

Naturally, tryptophan synthases depend on indole‐3‐glycerol phosphate (IGP) and serine as its substrates. The α‐subunit reversibly forms indole from IGP; afterwards, the β‐subunit catalyzes the pyridoxal phosphate‐dependent condensation of serine and indole to tryptophan. Via directed evolution, the β‐subunit was tailored towards a stand‐alone function, accepting indole instead of IGP with even higher activity than the natural enzyme.^[^ ^33^ ^]^ The thermophilic origin of the tryptophan synthase of *P. furiosus* ensures stability of the enzyme at high temperatures, which is advantageous for purification by heat‐denaturation and for enzymatic conversion of indole due to the increased solubility at elevated temperatures.

**Scheme 3:**
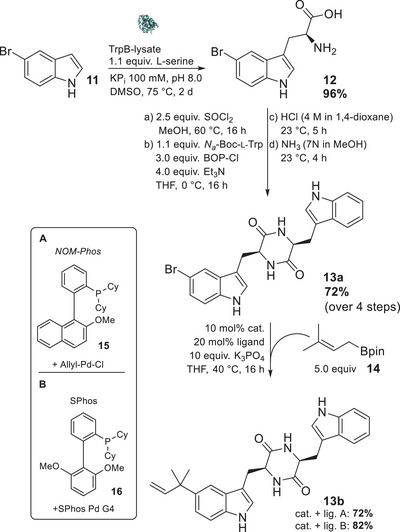
Synthesis of 5‐substituted cyclic Trp‐Trp‐DKPs **13a** and **13b** starting from 5‐bromoindole utilizing tryptophan synthase TrpB^Pf0A9^.

Among the many mutants that have been generated, TrpB^Pf0A9^ exhibited the highest activity for 5‐bromoindole (**11**) and, consequently, was the enzyme of choice. The desired 5‐bromotryptophan (**12**) was obtained in nearly quantitative yield (96%) after filtration of the crystallized product and, as previously reported,^[^ ^33^ ^]^ exclusively in its l‐form (Scheme 3).

To prevent oligomerization when coupling two amino acids, the carboxy functionality was protected as an ester (**S1**—see supporting information for characterization of the intermediate products). Using bis(2‐oxo‐3‐oxazolidinyl)phosphinic chloride (BOP‐Cl), the dipeptide (**S2**) was formed with orthogonally protected *tert*‐butyloxycarbonyl (Boc) tryptophan. Deprotection of the Boc group (**S3**) and subsequent treatment with ammonia in methanol gave the DKP **13a** in good yields (72% over four steps) (Scheme 3). Notably, no further purification was necessary within these steps. In order to prevent a loss of activity in the subsequent cross‐coupling as well as the enzymatic conversion by SgMT, the 5‐bromo‐cWW (**13a**) was chromatographically purified at this juncture. Furthermore, the purification process did only yield the l,l‐cww‐dkp **13a** while none of the d,l‐diastereomer **13e** was isolated, thereby confirming the high enantioselectivity (>95%) of TrpB^Pf0A9^. Afterwards, the brominated compound **13a** was subjected to a Suzuki–Miyaura cross‐coupling with prenyl pinacolborane (**14**). According to the presented catalyst systems by Yang and Buchwald, the palladium‐catalyzed cross‐coupling is regioselective depending on the used ligand: Only the reverse‐prenylated product **13b** is obtained when employing a 2′‐dicyclohexylphosphino‐2‐methoxy‐1‐phenylnaphthalene (**15**, *NOM‐*Phos) ligand.^[^ ^34^ ^]^ Therefore, several catalysts and ligands have been tested on a simple model compound (prenylation of Boc‐protected 5‐bromo indole (**S5**) showed that SPhos/SPhos Pd G4 (**16**) acts superior to *NOM‐Phos* (see Table S2).

Two catalyst/ligand combinations were applied for the prenylation of 5‐bromo‐cWW‐DKP **13a,** giving the prenylated DKP **13b** in 82% and 72% yield, respectively.

### 2.2 Enzymatic Conversion of Functionalized Diketopiperazines and Optimization Study

**Figure 1:**
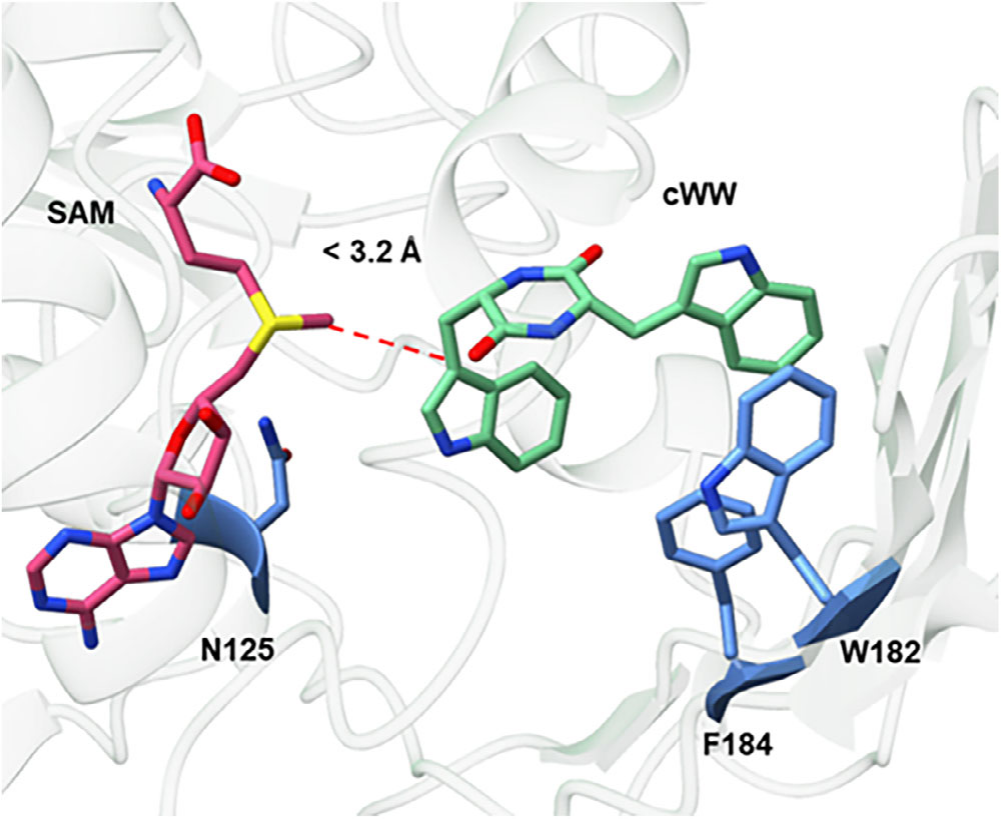
Catalytic site of SgMT (PDB:9GDJ)^[^ ^26^ ^]^ with SAM (pink) and the cWW (green). The mutated residues N125, W182, and F184 are highlighted in blue.

The resulting 5‐Br‐, 5‐prenyl, and 5‐Cl substituted cWW (the latter were synthesized from racemic tryptophan—see supporting information **13c**/**13e**/**13f**) were then tested regarding their activity in methyltransferase‐catalyzed reactions. The activity was determined with the bioluminescence‐based MTase‐Glo Assay (Promega), which detects the formation of *S*‐adenosyl‐l‐homocysteine (SAH) as a by‐product of the main methyltransferase reaction.^[^ ^35^ ^]^ The purified methyltransferase SgMT was obtained by heterologous gene expression in *E. coli* BL21(DE3) and purification via immobilized metal affinity chromatography on a Ni‐NTA column. The unsubstituted cWW (R = H, **13d**) was determined in a previous study as the best accepted substrate of SgMT, serving as a reference for this advanced substrate scope.^[^ ^26^ ^]^ Compared to this substrate, the substituted cWWs are larger in size. For the methyl transfer to happen with a methyltransferase like SgMT, specific criteria need to be fulfilled: According to previous studies, the distance between the methyl group on the SAM and the C3 position on the indole needs to be less than 3.2 Å. In addition, the angle between the transferred methyl group on the sulfur of the SAM and the C3 position on the indole, as well as the distance between the catalytic residues (Y126, H220, and D218), play an important role (Figure 1).^[^ ^24^ ^]^ Changing one parameter by adding a substituent to the substrate can have a huge impact on the activity of the enzyme.

**Figure 2:**
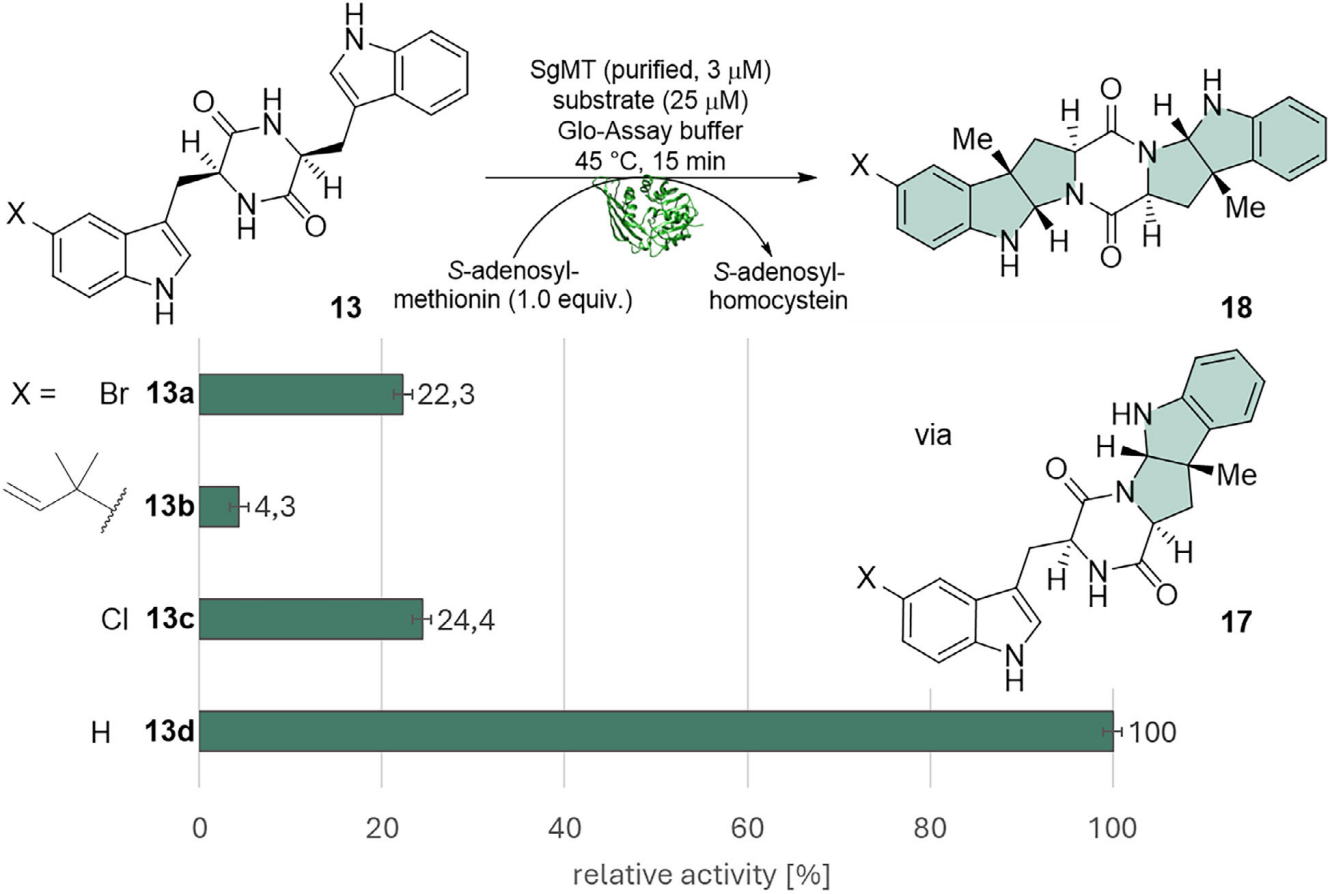
Relative activity of SgMT for the synthesized functionalized DKPs **13a‐c** in comparison to the unsubstituted substrate **13d** measured with the MTase Glo‐Assay (Promega). HHPIs **17** and **18** were formed.

For all substituted cWWs, the activity of the enzyme is decreased compared to the unsubstituted cWW **13d** (Figure 2). The halogenated cWW showed 22.3% (R = Br, **13a**) and 24.4% (R = Cl, **13c**) residual activity; the prenylated cWW **13b** only 4.3%. The size of the substituent influences the conversion rate of the enzyme significantly. Since the established prenylation conditions were also tested for the chlorinated DKP **13c** but did not yield any product, the chlorinated compound would require additional steps for a successful prenylation, such as conversion into, e.g., an iodide. Thus, 5‐Br cWW (**13a**) was investigated further as a substrate for SgMT.

To improve the rate of the enzyme towards compound **13a,** a mutagenesis study was implemented. An alanine scan of the binding site of the cWW was performed to find suitable amino acids for the mutagenesis experiments of seven positions close to the cWW binding site (Figure S2). One position on the catalytic site of the cWW (N125) and two positions on the outer side of the binding position of the cWW (W182 and F184) were chosen as a starting point (Figure S2). N125 and F184 showed residual activity in the alanine scan, proving that mutagenesis of these residues does not inactivate the enzyme directly. W182 is the residue taking the most space in the outer area of the binding site close to the potential substituent on the indole ring. In a pre‐experiment, W182 was mutated to phenylalanine, leaving a residual activity (Figure S3). Each position was mutated to all amino acids using the 22c trick. This approach minimizes codon redundancy, resulting in reduced mutant libraries.^[^ ^36^, ^37^ ^]^ After transformation of *E. coli* BL21(DE3) with the generated plasmid library, 66 colonies were picked and cultivated in autoinduction media.

As a positive control, the wild type was cultivated under the same conditions. The empty vector cells served as negative controls. The cells were harvested, and the reaction mixture containing buffer, SAM and the 5‐Br cWW (**13a**) was applied. To determine the activity of the mutants, a colorimetric indole detection assay was used.^[^ ^38^ ^]^ The assay determines the indole substrate left in the solution by forming a colored solution (Figure S4). The wells with the lowest absorption, meaning the highest conversion, were sequenced. For W182 and N125, none of the mutants showed a higher conversion than the wild type (Figures S6 and S7). The most promising mutant library was the library of F184 (Figure S8). The five mutants with the highest conversion in this experiment (mutants 1, 17, 33, 40, and 62—see supporting information) were sequenced: Four of the sequenced hits were determined to be the mutant F184L one mutant F184A. The mutants were purified, and the activity was measured with the Glo‐Assay as described before. As substrates, the 5‐Br cWW **13a** and the single C3‐methylated 5‐Br cWW **17a** were chosen. Only the mutant F184L showed an improved conversion for the 5‐Br cWW (12%), but this mutant had a lower activity for the second methylation step compared to the wild type (−26%) (Figure S9). For the total synthesis of lansai B (**3**), which is methylated on both sides, the wild type still is the best option. Nevertheless, the enlarged alanine scan and the mutagenesis study give further insights into the active center of SgMT.

**Figure 3:**
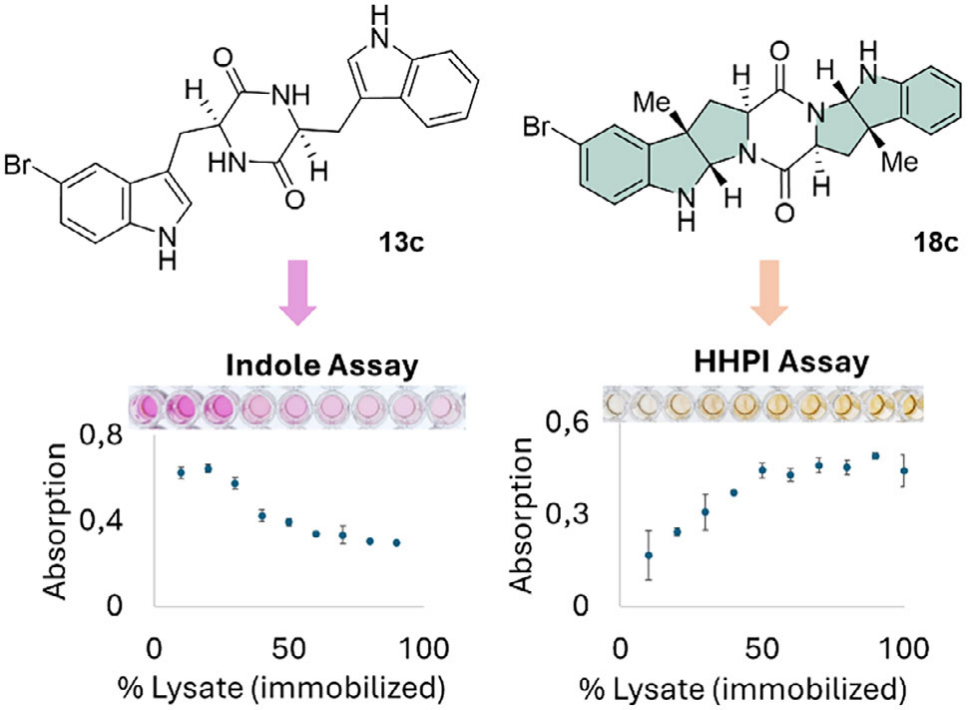
Optimization of the biocatalytic methylation reaction of SgMT with the 5‐bromo cWW **13c**. Two assays were used to detect the substrate **13c** (indole assay, left) and the product **18c** (HPPI assay, right) in parallel via absorption.^[^ ^38^ ^]^ The amount of immobilized lysate of SgMT cells was varied.

To optimize the methylation reaction of the 5‐Br cWW (**13a**) with SgMT, the optimal amount of enzyme was determined (Figure 3). For this, the methyltransferase was immobilized directly out of the lysate with Ni‐NTA agarose beads. Different amounts of lysates of the SgMT‐containing cells (10 vol% – 100 vol%) were used in small‐scale reactions. After 20 h, the reactions were stopped. From each transformation, two samples were analyzed via the indole assay and an additional HHPI assay, which detects the amount of product formed via a reaction with cerium sulfate.^[^ ^38^ ^]^ This way, conversion of the substrate and product formation is quantified by absorption measurement independently. Shown in Figure 3, 70 Vol% immobilized SgMT lysate is sufficient to achieve maximal conversion of the brominated cWW‐DKP (**13a**).

### 2.3 Preparative Application of the Methyltransferase and Completion of the Total Synthesis

After the enzymatic key step was investigated in detail and the conditions were optimized towards high conversion of the brominated cWW‐DKP **13a**, the methyltransferase was applied at preparative scale. The methyltransferase reaction was performed on a 50 mg scale under optimized conditions, comprising 70% immobilized SgMT lysate, 3 mM SAM in K*P_i_* buffer at pH 8 at 40°C, and a reaction time of 1 day. This resulted in the complete conversion of the substrate. Following the extraction process, 79% of the desired double methylated product was formed, while only 6% of the monomethylated intermediate was obtained.

Structural analysis of the intermediate confirmed that the methyl group is attached to the unsubstituted indole‐moiety exclusively, thus showing matching NMR shifts as the already described compound **17d** (single methylated product of **13d**).^[^ ^24^ ^]^ In accordance with the published data, only one diastereomer was obtained for both of the two products, **17a** and **18a**. As demonstrated in the corresponding publication, MD simulations have shown that the methyltransferase requires a precise orientation of the substrate and SAM (see Figures S1 and S2). This results in the formation of the exo(trans)‐configurated product during the conversion of l,l‐cWW‐DKP.^[^ ^24^ ^]^

In addition to the Suzuki coupling yielding prenylated product **19**, the remaining conversion necessary for completion of the overall synthesis is represented by the *N*‐methylation. Thus, many different methylation conditions were tested on the unsubstituted HHPI DKP **S13** (Table S3) as a model compound.

However, HHPI **S13** displayed only low reactivity when subjected to a nucleophilic substitution with methyl iodide, even when a potent base such as potassium *tert*‐butoxide was employed. Elevated temperatures under Eschweiler–Clarke conditions utilizing microwave heating also proved disadvantageous, since this led to decomposition. Notably, Eschweiler–Clarke conditions^[^ ^39^ ^]^ were applied to a smaller HHPI **S11,** which did not contain the DKP ring, resulting in a moderate 54% yield of the *N*‐methylated product.

In pursuit of a reactive methylation species suitable under mild conditions, a combination of proton sponge (**20**) as base and Meerwein's salt (**21**) was identified as the optimal choice for the final methylation. The high amounts of reagent were added subsequently since the reaction tended to stop after a certain amount of time. Eventually, a yield of 72% was isolated for the DKP model compound **S14**. The tested reaction conditions as well as full analytics for the two model compounds are shown in the supporting information (chapter 2.5).

With this method in hand, *N*‐methylation of the 5‐brominated compound **18a** was attempted first. Despite the gradual addition of 12 equivalents of proton sponge and 9.0 equivalents of Meerwein's salt in total, completion of the reaction was not achieved, leaving a mixture of substrate, single and double methylated product **S15** (the latter with 33% isolated yield). Presumably the electron‐withdrawing bromine‐substituent reduces the nucleophilicity of the HHPI nitrogen.

**Scheme 4:**
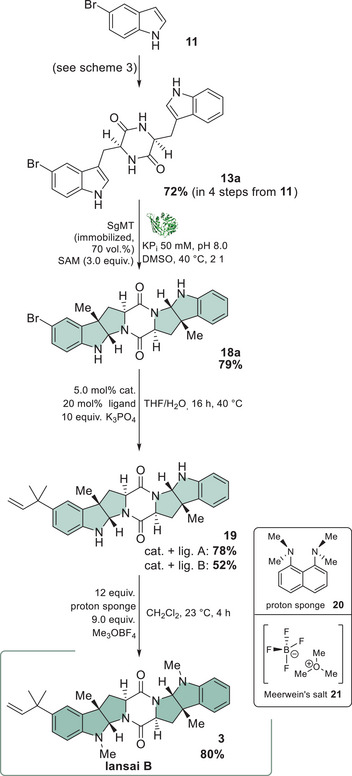
Final steps of the enantioselective synthesis of lansai B (**3**).

Therefore, prenylation was performed prior to *N*‐methylation by application of the same two catalyst systems as for DKP **13a** (described in Scheme 3). The prenylated product **19** was obtained with a yield of 78% using the naphthyl ligand, while the SPhos catalyst and ligand only exhibited moderate conversion (52%) (Scheme 4). This demonstrates the electronic disparities between the pyrroloindoline and indole, since the latter showed higher conversion applying catalytic system B within the screening (supporting information, chapter 2.4.2) and was therefore used for the prenylation of compound **13a** (Scheme 3). Using 12 equivalents of base **20** and 9.0 equivalents of Meerwein's salt (**21**) finally gave *N*‐methylated product lansai B (**3**) in 80% yield.

Thus, the substituent in 5‐position highly affects the electronic properties of the HHPI compounds so that prenylation is highly recommended prior to *N*‐methylation. However, even when prenylated, HHPI **19** exhibits only moderate activity during *N*‐methylation and is susceptible to degradation, which necessitates the use of raised amounts of methylation agent and preferably short reaction times. Despite the challenges mentioned, the prenylated DKP **19** was successfully transformed into the intended natural product lansai B (**3**) in a high yield, thus concluding the total synthesis. The results obtained from NMR spectroscopy and optical activity are consistent with the previously reported data.^[^ ^14^ ^]^ This finding serves to reinforce the conclusion that the natural product was synthesized in the correct exo(trans)‐configuration. Consequently, this outcome validates the utility of the methyltransferase SgMT in synthetic processes.

## 3 Conclusion

The natural compound lansai B (**3**) was obtained in eight steps from 5‐bromoindole (**11**), with only four chromatographic purification steps being necessary. The formation of the HHPI via methyltransferase was feasible in high yields, although not the natural substrate was used, but a derivative thereof. Thus, an efficient shortcut in the synthesis of the challenging HHPI framework was installed, also proving that methyltransferases can be beneficial tools in the total synthesis of natural products. Furthermore, the early formation of the DKP and the late‐stage functionalization of the brominated intermediate provide convenient access to novel DKPs.

## Supporting Information

Detailed information including synthetic procedures as well as biological methods are provided together with additional figures as cited in the manuscript and nmr spectra of all compounds. The authors have cited additional references within the Supporting Information.^[^ ^7^, ^14^, ^24^, ^26^, ^30^, ^31^, ^34^, ^40^, ^41^, ^42^, ^43^ ^]^

## Conflicts of Interest

The authors declare no conflicts of interest.

## Supporting information



Supporting Information

## Data Availability

The data that support the findings of this study are available from the corresponding author upon reasonable request.
