# Supplementary material for: Chemoenzymatic Total Synthesis of Lansai B
Source: Chemistry. 2025 Apr 16;31(26):e202500740. doi: 10.1002/chem.202500740 (PMC12063042; doi:10.1002/chem.202500740)
Supplement: Supplementary file 1 — Supporting Information [file CHEM-31-e202500740-s001.pdf]

Table of Contents

1. **List of tables/figures** S3
2. **Chemical Methods** S5
   - 2.1. General Information S5
   - 2.2. Substrate synthesis from racemic 5-functionalized tryptophan mixtures for enzyme scope and pretests S6
     - 2.2.1. cWW-DKP-Synthesis from racemic 5-chloro-tryptophan (**13c+f**) S7
     - 2.2.2. cWW-DKP-Synthesis from racemic 5-bromo-tryptophan (**13a+e**) S9
   - 2.3. Enantiomerically pure substrate synthesis utilizing $TrpB^{Pf0A9}$ S12
     - 2.3.1. Formation of 5-bromo-tryptophan (**12**) with TrpB Pf0A9 .... S12
     - 2.3.2. Formation of *O*-Me protected Tryptophan (**S1**) S13
     - 2.3.3. Dimerization / Formation of peptide bond towards dipeptide **S2** S14
     - 2.3.4. *N*-Deprotection towards Amine **S3** S15
     - 2.3.5. *N*-Deprotection and cWW-DKP-Formation (**13a**) S16
   - 2.4. Suzuki-Coupling S17
     - 2.4.1. Preparation of Prenyl-pinacolborane (**14**) S17
     - 2.4.2. Screening of different catalyst-system S18
     - 2.4.3. Suzuki-Coupling to form 5-prenyl-cWW-DKP (**13b**) S20
     - 2.4.4. Enzymatic conversion of 5-bromo-cWW-DKP (**13a**) in preparative scale to pyrroloindole **17a** and **18a** S22
   - 2.5. Screening for *N*-Methylation-Conditions S24
   - 2.6. *N*-Methylation towards 5-bromo-hexahydropyrroloindole **S15** S28
   - 2.7. Suzuki coupling towards 5-prenyl-hexahydropyrroloindol **19** S30
   - 2.8. *N*-Methylation of 5-prenyl-hexahydropyrroloindole-DKP to (-)-lansai B (**3**) S32
3. **Biological Methods** S34
   - 3.1. Protein sequence S34
   - 3.2. Bacterial strains and media S35
   - 3.3. Proteinexpression S36
   - 3.4. Mutagenesis S36
   - 3.5. Enzyme purification of SgMT S37
   - 3.6. General procedure for Mtase glo assay S37
   - 3.7. Enzymatic preparative scale methylation reaction with immobilized SgMT .. S37
   - 3.8. Reaction optimization (MTase reaction) S38
     - 3.8.1. HPI Assay S38
     - 3.8.2. Indole Assay S38
   - 3.9. Preparation of tryptophan synthase for preparative scale reactions S38

3.10. Automated Screening Process S39
4. Appendix S40
4.1. Additional figures S40
4.2. NMR-spectra S43
4.3. References S65

1. List of tables/figures

Table S1: Comparison of the analytical data of the diastereomeric diketopiperazines **13a-13f** 11
Table S2: Screened catalyst and ligand combinations for the reverse-prenylation 19
Table S3: Screened reaction conditions for the *N*-methylation of bis-hexahydropyrroloindole-DKPs 25

Figure S1: Proposed mechanism for the reaction of SgMT. The cWW substrate is highlighted in green, SAM in pink and the residues of the catalytic triade in blue. 40
Figure S2: Catalytic side of SgMT (PDB: 9GDJ) with SAM (dark pink), the cWW (green) and the amino acids tested in the alanine scan (blue).[26] 40
Figure S3: Activity of SgMT variants for the cWW substrate measured with the MTase Glo-Assay (Promega). 41
Figure S4: Principle of the indole-Assay: The indolic substrate reacts with the DMAB forming a colorimetric product under acidic conditions in presence of iPrOH.The assay was carried out in a 96 well plate format. 41
Figure S5: Layout of the 96 well plate format for the mutagenesis study: 66 variants were tested plus two negative (empty vector) and two positive contols (wt). 41
Figure S6: Results of the mutagenesis study for position N125. The indole assay was used to form the colorimetric product, which was measured via absorption at 540 nm. 42
Figure S7: Results of the mutagenesis study for position W182. The indole assay was used to form the colorimetric product, which was measured via absorption at 540 nm. 42
Figure S8: Results of the mutagenesis study for position F184. The indole assay was used to form the colorimetric product, which was measured via absorption at 540 nm. 42
Figure S9: Activity of SgMT variants for the 5-Bromo-cWW substrate (blue) and the single methylated 5-Bromo-cWW intermediate (orange) measured with the MTase Glo-Assay (Promega). 42
Figure 10: 1H-NMR-spectrum (600 MHz) of **13c** in CD_3OD. 43
Figure S11: ^{13}C-NMR-spectrum (151 MHz) of **13c** in CD_3OD. 43
Figure S12: ^{1}H-NMR-spectrum (600 MHz) of **13f** in CD_3OD. 44
Figure S13: ^{13}C-NMR-spectrum (151 MHz) of **13f** in CD_3OD. 44
Figure S14: ^{1}H-NMR-spectrum (600 MHz) of **13a** in CD_3OD. 45
Figure S15: ^{13}C-NMR-spectrum (151 MHz) of **13a** in CD_3OD. 45
Figure S16: ^{1}H-NMR-spectrum (600 MHz) of **13e** in CD_3OD. 46
Figure S17: ^{13}C-NMR-spectrum (151 MHz) of **13e** in CD_3OD. 46
Figure S18: ^{1}H-NMR-spectrum (600 MHz) of **12** in CD_3OD. 47
Figure S19: ^{13}C-NMR-spectrum (151 MHz) of **12** in CD_3OD. 47
Figure S20: ^{1}H-NMR-spectrum (600 MHz) of **S1** in CD_3OD. 48
Figure S21: ^{13}C-NMR-spectrum (151 MHz) of **S1** in CD_3OD. 48
Figure S22: ^{1}H-NMR-spectrum (600 MHz) of **S2** in CD_3OD. 49
Figure S23: ^{13}C-NMR-spectrum (151 MHz) of **S2** in CD_3OD. 49
Figure S24: Crude ^{1}H-NMR-spectrum (600 MHz) of **S3** in CD_3OD. 50
Figure 25: Crude ^{13}C-NMR-spectrum (151 MHz) of **S3** in CD_3OD. 50
Figure S26: ^{1}H-NMR-spectrum (600 MHz) of **14** in $CDCl_3$. 51
Figure S27: ^{13}C-NMR-spectrum (151 MHz) of **14** in $CDCl_3$. 51
Figure S28: Crude ^{1}H-NMR-spectrum (600 MHz) of **S6** in $CDCl_3$. 52
Figure S29 : ^{1}H-NMR-spectrum (600 MHz) of **13b** in CD_3OD. 53

Figure S30: ^{13}C-NMR-spectrum (151 MHz) of **13b** in CD_3OD. 53
Figure S31: ^{1}H-NMR-spectrum (600 MHz) of **17a** in CD_3OD. 54
Figure S32: ^{13}C-NMR-spectrum (151 MHz) of **17a** in CD_3OD. 54
Figure S33: ^{1}H-NMR-spectrum (600 MHz) of **18a** in CD_3OD. 55
Figure S34: ^{13}C-NMR-spectrum (151 MHz) of **18a** in CD_3OD. 55
Figure S35: Crude 1H-NMR-spectrum (600 MHz) of S12 in CD3OD. 56
Figure S36: Crude 13C-NMR-spectrum (151 MHz) of S12 in CD3OD. 56
Figure S37: Synthethic ^{1}H-NMR-spectrum (600 MHz) of **S12** in CD_3OD. 57
Figure S38: Synthethic ^{13}C-NMR-spectrum (151 MHz) of **S12** in CD_3OD. 57
Figure S39: 1H-NMR-spectrum (600 MHz) of **S14** in $CDCl_3$. 58
Figure S40 ^{13}C-NMR-spectrum (151 MHz) of **S14** in CD_3OD 58
Figure S41: ^{1}H-NMR-spectrum (600 MHz) of **S15** in CD_3OD. 59
Figure S42: ^{13}C-NMR-spectrum (151 MHz) of **S15** in CD_3OD. 59
Figure S43: ^{1}H-NMR-spectrum (600MHz) of **S15** in $CDCl_3$. 60
Figure S44: ^{13}C-NMR-spectrum (151 MHz) of **S15** in $CDCl_3$. 60
Figure S45: ^{1}H-NMR-spectrum (600 MHz) of **19** in CD_3OD. 61
Figure S46: ^{13}C-NMR-spectrum (151 MHz) of **19** in CD_3OD. 61
Figure S47: ^{1}H-NMR-spectrum (600 MHz) of **19** in $CDCl_3$. 62
Figure S48: ^{13}C-NMR-spectrum (151 MHz) of **19** in $CDCl_3$. 62
Figure S49 ^{1}H-NMR-spectrum (600 MHz) of Iansai B (**3**) in $CDCl_3$. 63
Figure S50: ^{13}C-NMR-spectrum (151MHz) of Iansai B (**3**) in $CDCl_3$. 63
Figure S51: ^{1}H-NMR-spectrum (700 MHz) of Iansai B (**3**) in $CDCl_3$. 64
Figure S52: ^{13}C-NMR-spectrum (175 MHz) of Iansai B (**3**) in $CDCl_3$ 64

2. Chemical Methods

2.1. General Information

All used chemicals were obtained from commercial sources and were not subjected to any further purification processes. Inert conditions were maintained by utilizing dried solvents from a MB-SPS-800 solvent purification system. In order to exclude oxygen and water, flame-dried glassware was utilized in conjunction with an inert atmosphere (N_2 or Ar). For the purpose of reaction control, thin layer chromatography (TLC) was employed, utilizing a Polygram SilG/UV254 stationary phase and a mixture of ethyl acetate and petroleumether as the mobile phase. The TLC plates were stained using a cerium molybdate solution, composed of 10 g $Ce(SO_4)_2 \cdot 4H_2O$, 25 g phosphomolybdic acid, 60 mL conc. H_2SO_4, and 940 mL H_2O. Purification was conducted using preparative column chromatography with silica gel (0.040–0.063 mm) supplied by Merck as the stationary phase and the stated solvent mixtures as the mobile phase. The optical rotation of the synthesized compounds was determined utilizing a JASCO P-2000 polarimeter. The ^1H, ^{13}C and 2D NMR (COSY, HSQC, HMBC) spectra of the samples were measured using a Bruker Avance III HD (700 MHz), Bruker Avance DRX 600 (MHz) or Bruker Avance DRX 300 (MHz) NMR spectrometer, with the samples dissolved in deuterated chloroform or deuterated methanol. The solvent peak or the internal standard (tetramethylsilane) peak were employed as the reference peak. For the measurement of IR spectra, a PerkinElmer SpectrumTwo spectrometer was utilized. High-resolution ESI mass spectra were obtained by the HHU Center of Molecular and Structural Analytics at the Heinrich-Heine-Universität Düsseldorf, using an MDS SCIEX Q Model Trap 4000 mass spectrometer.

2.2. Substrate synthesis from racemic 5-functionalized tryptophan mixtures for enzyme scope and pretests

For initial experiments, the cWW-DKP susbtrates were synthesized from racemic 5-bromo/chloro-tryptophan. The obtained diastereomers were chromatographically separated after formation of the diketopiperazine ring. Only the L,L-5-bromo diketopiperazine (**13a**) was converted into the prenylated diketopiperazine **13b**

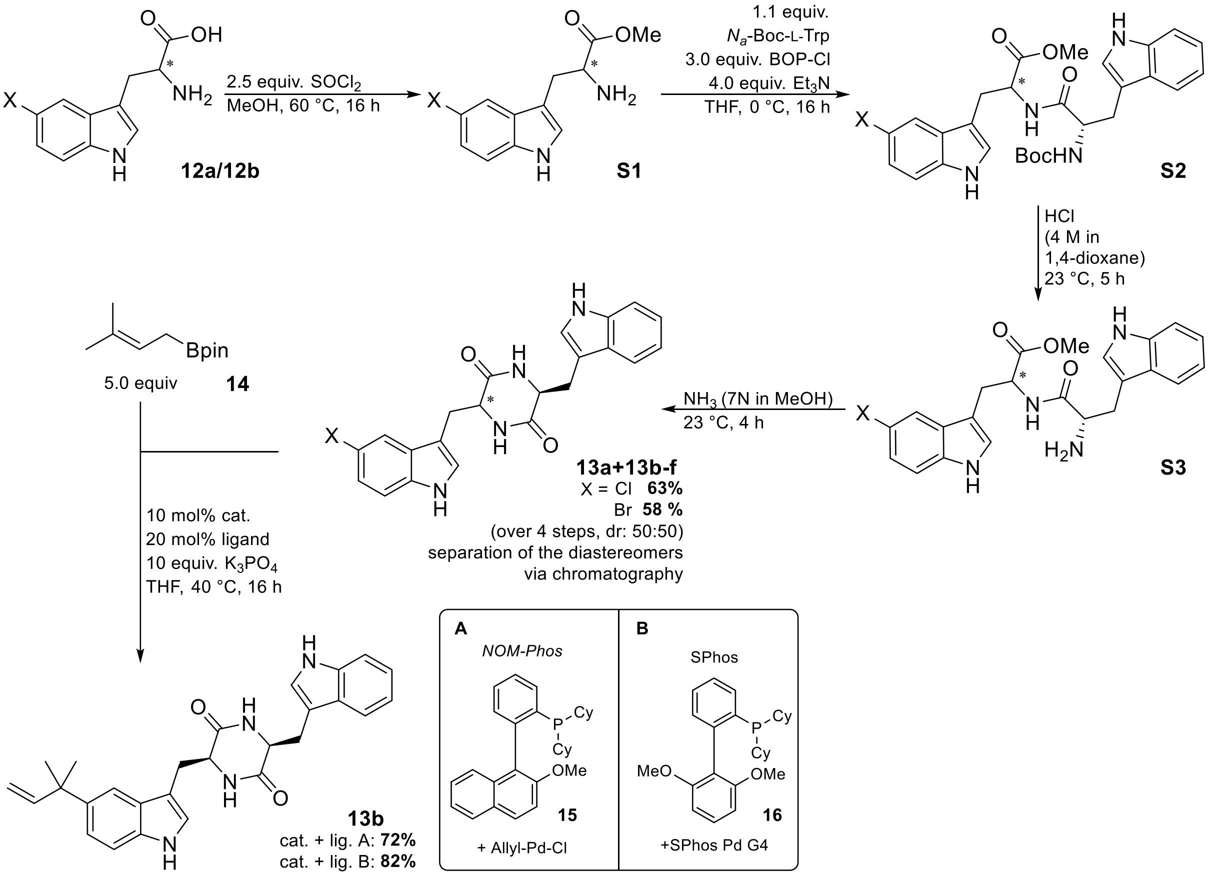

Scheme S1: Initial synthesis of functionalized diketopiperazines from racemic 5-chloro- (**12a**) and 5-bromo-tryptophan (**12b**) leading to diastereomeric diketopiperazines **13a**/**13e** (5-bromo) and **13c**/**13f** (5-chloro).

2.2.1. cWW-DKP-Synthesis from racemic 5-chloro-tryptophan (13c+f)

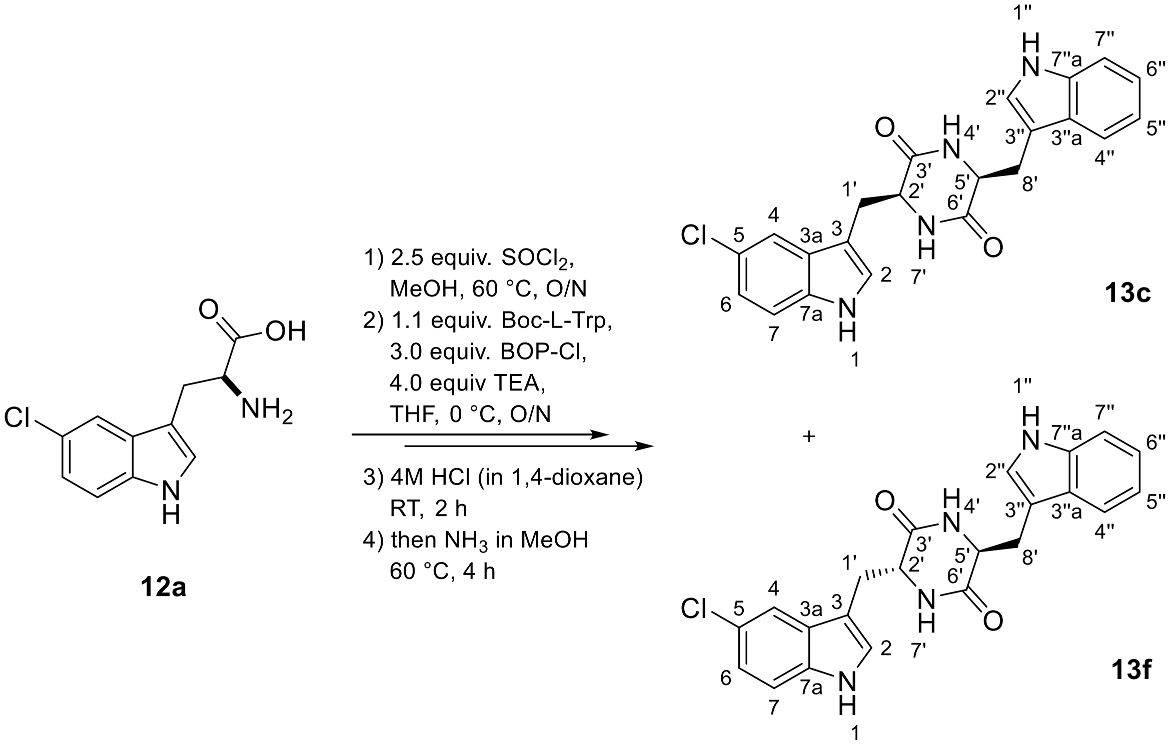

First step: Racemic 5-chloro-DL-tryptophan (840 mg, 3.5 mmol, 1.0 equiv.) was given to a 100 mL heat-dried round-bottomed flask and dissolved in dried methanol (30 mL). To the solution thionyl chloride (0.65 mL, 8.9 mmol, 2.5 equiv.) was added cautiously while stirring. The solution was then heated to 60 °C for 18 hours. Then, the mixture was cooled down, diluted with ethyl acetate (25 mL) and neutralized by addition of an aqueous, saturated solution of sodium carbonate (20 mL). The organic phase was separated and the aqueous phase was extracted with ethyl acetate (3x10 mL). Afterwards, the organic phases were combined, washed with an aqueous, saturated solution of sodium chloride, dried over magnesium sulfate and the solvent removed.

Second Step: The crude product from step 1 (810 mg, 3.2 mmol, 1.0 equiv.) and N_α-(*tert.*-butoxycarbonyl)-L-tryptophan (975 mg, 3.2 mmol, 1.0 equiv.) were given to a 100 mL heat-dried round bottomed flask and dissolved in dried tetrahydrofuran (30 mL). The solution was stirred at 0 °C and subsequently triethylamine (1.8 mL, 12.8 mmol, 4.0 equiv.) and bis-(2-oxo-3-oxazolidinyl) phosphinic chloride (2.5 g, 9.6 mmol, 3.0 equiv.) were added. The solution was stirred for 18 hours at 23 °C. Then, the mixture was cooled down, diluted with ethyl acetate (40 mL) and neutralized by addition of an aqueous, saturated solution of ammonium chloride (20 mL). The organic phase was separated and the aqueous phase was extracted with ethyl acetate (5x15 mL). Afterwards, the organic phases were combined, washed with an aqueous, saturated solution of sodium chloride, dried over magnesium sulfate and the solvent removed under reduced pressure.

Third step: The crude product from step 2 (1.42 g, 2.6 mmol, 1 equiv.) was dissolved in THF (2 mL) and hydrogen chloride (4M in 1,4-dioxane, 8 mL) and stirred for 2 hours. Then, the solvent was removed under reduced pressure.

Fourth step: The crude product from step 3 (1.1 g, 2.4 mmol, 1.0 equiv.) was dissolved in NH_3 (7N in methanol, 8 mL) and stirred at 60 °C for 4 hours. Then, the solvent was removed under reduced pressure. After chromatographic purification (97/3 ethyl acetate/methanol) the two diastereomers of 5-chloro-cWW-DKP were obtained individually as white crystals.

Yield: 908 mg, 2.3 mmol, 63% (Overall yield of both diastereomers combined).

5-Chloro-LL-cWW-DKP (**13c**)

^{1}H-NMR (600 MHz, CD_3OD) δ 7.52 (d, $^4J_{4,6}$ = 2.0 Hz, 1H, 4-H), 7.44 (dd, $^3J_{4“,5“}$ = 8.0 Hz, $^4J_{4“,6“}$ 1.0 Hz, 1H), 7.32 (dd, $^3J_{7“,6“}$ = 8.2 Hz, $^4J_{7“,5“}$ = 0.9 Hz, 1H, 7“-H), 7.27 (d, $^3J_{7,6}$ = 8.6 Hz, 1H, 7-H), 7.11 (ddd, $^3J_{6“,7“}$ = 8.1 Hz, $^3J_{6“,5“}$ = 7.1 Hz, $^4J_{6“,4“}$ = 1.2 Hz, 1H, 6“-H), 7.06 (dd, $^3J_{6,7}$ = 8.6 Hz, $^4J_{6,4}$ = 2.0 Hz, 1H, 6-H), 7.03 (ddd, $^3J_{5“,4“}$ = 7.9 Hz, $^3J_{5“,6“}$ = 7.0 Hz, $^4J_{5“,7“}$ = 1.0 Hz, 1H, 5“-H), 6.63 (s, 1H, 2“-H), 6.45 (s, 1H, 2-H), 4.08 (ddd, $^3J_{2‘,1‘}$ = 7.1, $^3J_{2‘,1‘}$ = 3.9, $^3J_{2‘,NH}$ = 1.1 Hz, 1H, 2‘-H), 4.03 (ddd, $^3J_{5‘,8‘}$ = 7.4 Hz, $^3J_{5‘,8‘}$ = 4.0 Hz, $^3J_{5‘,NH}$ = 1.1 Hz, 1H, 5‘-H), 2.96 (dd, $^2J_{1‘,1‘}$ = 14.5, $^3J_{1‘,2‘}$ = 3.9 Hz, 1H, 1’-H$_a$), 2.85 (dd, $^2J_{8‘,8‘}$ = 14.5 Hz, $^3J_{8‘,5‘}$ = 3.9 Hz, 1H, 8’-H$_a$), 2.28 (dd, $^2J_{1‘,1‘}$ = 14.4 Hz, $^3J_{1‘,2‘}$ = 7.0 Hz, 1H, 1’-H$_a$), 2.07 (dd, $^2J_{8‘,8‘}$ = 14.5 Hz, $^3J_{8‘,5‘}$ = 7.3 Hz, 1H, 8’-H$_b$).

^{13}C-NMR (151 MHz, CD_3OD.) δ 169.7 (C-3‘/6‘), 169.7 (C-6‘/3‘), 138.1 (C-7“a), 136.4 (C-7a), 129.7(C-3a), 128.7 (C-3”a), 127.6 (C-2), 125.9 (C-5), 125.8 (C-2”), 122.7 (C-6/6”), 122.6 (C-6”/6), 120.2 (C-5”), 119.7 (C-4”), 119.1 (C-4), 113.6 (C-7), 112.5 (C-7”), 109.6 (C-3), 109.5 (C-3”), 57.0 (C-2’), 56.6 (C-5’), 31.4 (C-1’), 31.3 (C-8’).

IR (ATR): ṽ [cm^{-1}] = 3340 (br), 2930 (m), 1667 (vs), 1453 (s), 1330 (s), 1080 (m), 741 (s).

HRMS (ESI): m/z calc. for $C_{22}H_{19}ClN_4O_2+H^+$: 407.1269 [M+H]$^+$; found: 407.1259.

Optical Rotation: $[\alpha]_D^{20}$ = -79 (c = 0.192, MeOH).

Melting Point: 207 °C.

5-Chloro-DL-cWW-DKP (**13f**)

^{1}H-NMR (600 MHz, CD_3OD) δ 7.49 – 7.46 (m, 2H, 4-H, 5”-H), 7.31 (dd, $^3J_{7”,6”}$ = 8.2 Hz, $^4J_{7”,5”}$ = 0.9 Hz, 1H, 7”-H), 7.26 (d, $^3J_{7,6}$ = 8.6 Hz, 1H, 7-H), 7.09 – 7.03 (m, 1H, 6”-H), 7.02 (dd, $^3J_{6,7}$ = 8.6 Hz, $^4J_{6,4}$ = 2.1 Hz, 1H, 6-H), 6.98 – 6.94 (m, 3H, 2-H, 2”-H, 4”-H), 3.54 – 3.50 (m, 1H, 2’-H), 3.41 – 3.33 (m, 1H, 5’-H), 3.23 (dd, $^2J_{1’,1’}$ = 14.8 Hz, $^3J_{1’,2’}$ = 4.5 Hz, 1H, 1’-H$_a$), 3.15 (dd, $^2J_{8’,8’}$ = 14.9 Hz, $^3J_{8’,5’}$ = 4.6 Hz, 1H, 8’-H$_a$), 3.01 (dd, $^2J_{1’,1’}$ = 14.8 Hz, $^3J_{1’,2’}$ = 4.4 Hz, 1H, 1’-H$_b$), 2.92 (dd, $^2J_{8’,8’}$ = 14.9 Hz, $^3J_{8’,5’}$ = 4.4 Hz, 1H, 8’-H$_b$).

^{13}C-NMR (151 MHz, CD_3OD) δ 170.7 (C-6‘), 170.5 (C-3‘), 137.9 (C-7“a), 136.2 (C-7a), 129.9 (C-3a), 128.8 (C-3”a), 127.2 (C-2”), 125.8 (C-5), 125.7 (C-2), 122.7 (C-6), 122.5 (C-6”), 120.1 (C-5”), 119.7 (C-4”), 119.2 (C-4), 113.3 (C-7), 112.2 (C-7”), 109.2 (C-3”), 109.1 (C-3), 56.5 (C-2’), 56.2 (C-5’), 30.3 (C-1’), 29.7 (C-8’).

IR (ATR): ṽ [cm^{-1}] = 3420 (w), 3346 (br), 2924 (w), 1669 (vs), 1456 (s), 1315 (m), 1093 (w), 746 (m).

HRMS (ESI): m/z calc. for $C_{22}H_{19}ClN_4O_2+H^+$: 407.1269 [M+H]$^+$; found: 407.1259.

Optical Rotation: $[\alpha]_D^{20}$ = -5 (c = 0.415, MeOH).

Melting Point: 169 °C.

2.2.2. cWW-DKP-Synthesis from racemic 5-bromo-tryptophan (13a+e)

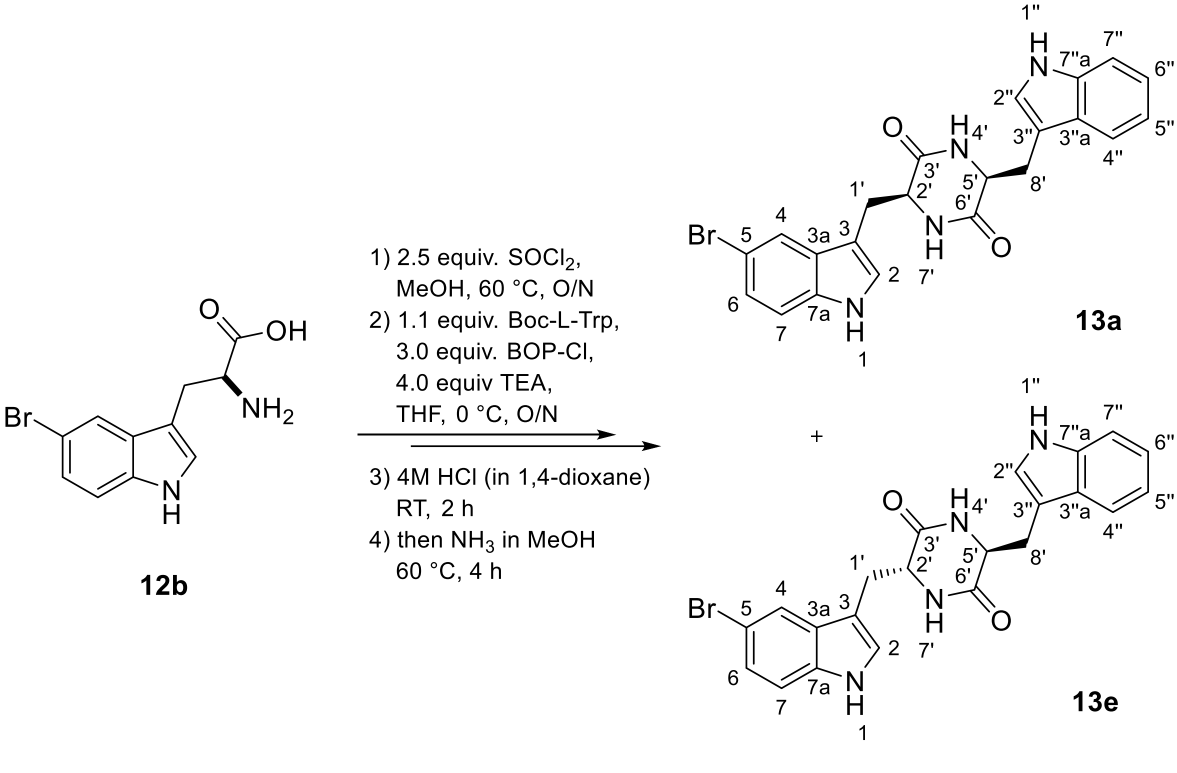

First step: Racemic 5-bromo-DL-tryptophan (1.0 g, 3.5 mmol, 1.0 equiv.) was given to a 100 mL heat-dried round-bottomed flask and dissolved in dried methanol (30 mL). To the solution thionyl chloride (0.64 mL, 8.9 mmol, 2.5 equiv.) was added cautiously while stirring. The solution was then heated to 60 °C for 18 hours. Then, the mixture was cooled down, diluted with ethyl acetate (25 mL) and neutralized by addition of an aqueous, saturated solution of sodium carbonate (20 mL). The organic phase was separated and the aqueous phase was extracted with ethyl acetate (3x10 mL). Afterwards, the organic phases were combined, washed with an aqueous, saturated solution of sodium chloride, dried over magnesium sulfate and the solvent removed.

Second Step: The crude product from step 1 (950 mg, 3.2 mmol, 1.0 equiv.) and N_{α}-(*tert.*-butoxycarbonyl)-L-tryptophan (973 mg, 3.2 mmol, 1.0 equiv.) were given to a 100 mL heat-dried round bottomed flask and dissolved in dried tetrahydrofuran (30 mL). The solution was stirred at 0 °C and subsequently triethylamine (1.8 mL, 12.8 mmol, 4.0 equiv.) and bis-(2-oxo-3-oxazolidinyl) phosphinic chloride (2.5 g, 9.6 mmol, 3.0 equiv.) were added. The solution was stirred for 18 hours at 23 °C. Then, the mixture was cooled down, diluted with ethyl acetate (40 mL) and neutralized by addition of an aqueous, saturated solution of ammonium chloride (20 mL). The organic phase was separated and the aqueous phase was extracted with ethyl acetate (5x15 mL). Afterwards, the organic phases were combined, washed with an aqueous, saturated solution of sodium chloride, dried over magnesium sulfate and the solvent removed under reduced pressure.

Third step: The crude product from step 2 (1.56 g, 2.6 mmol, 1.0 equiv.) was dissolved in THF (2 mL) and hydrogen chloride (4M in 1,4-dioxane, 8 mL) and stirred for 2 hours. Then, the solvent was removed under reduced pressure.

Fourth step: The crude product from step 3 (1.06 g, 2.2 mmol, 1.0 equiv.) was dissolved in NH_3 (7N in methanol, 8 mL) and stirred at 60 °C for 4 hours. Then, the solvent was removed under reduced pressure. After chromatographic purification (97/3 ethyl acetate/methanol) the two diastereomers of 5-bromo-cWW-DKP were obtained individually as white crystals.

Yield: 917 mg, 2.0 mmol, 58% (Overall yield of both diastereomers combined).

5-Bromo-LL-cWW-DKP: (**13a**)

^{1}H-NMR (600 MHz, CD_3OD) δ 7.68 (d, $^4J_{4,6}$ = 1.9 Hz, 1H, 4-H), 7.44 (d, $^3J_{4'',5''}$ = 8.0 Hz, 1H, 4"-H), 7.32 (d, $^3J_{7'',6''}$ = 8.2 Hz, 1H, 7"-H), 7.23 (d, $^3J_{7,6}$ = 8.6 Hz, 1H, 7-H), 7.19 (dd, $^3J_{6,7}$ = 8.6 Hz, $^4J_{6,7}$ 1.8 Hz, 1H, 6-H), 7.11 (t, $^3J_{6'','5,7''}$ = 7.6 Hz, 1H, 6"-H), 7.03 (t, $^3J_{5'',4'',6''}$ = 7.5 Hz, 1H, 5"-H), 6.64 (s, 1H, 2"-H), 6.42 (s, 1H, 2-H), 4.08 (dd, $^3J_{5',8'}$ = 7.0, $^3J_{5',8'}$ = 4.0 Hz, 1H, 5'-H), 4.02 (dd, $^3J_{2',1'}$ = 7.5, $^3J_{2',1'}$ = 3.9 Hz, 1H, 2'-H), 2.97 (dd, $^2J_{8',8'}$ = 14.4 Hz, $^3J_{8',5'}$ = 3.9 Hz, 1H, 8'-H_a), 2.84 (dd, $^2J_{1',1'}$ = 14.5 Hz, $^3J_{1',2'}$ = 4.0 Hz, 1H, 1'-H_a), 2.29 (dd, $^3J_{8',8'}$ = 14.4 Hz, $^3J_{8',5'}$ = 7.0 Hz, 1H, 8'-H_b), 2.03 (dd, $^2J_{1',1'}$ = 12.6 Hz, $^3J_{1',2'}$ = 6.3 Hz, 1H, 1'-H_b).

^{1}H-NMR (151 MHz, CD_3OD) δ 173.1 (C-3'), 170.1 (C-6'), 138.3 (C-7"a), 136.7 (C-7a), 130.3 (C-3a), 128.3 (C-3"a), 126.2 (C-2), 125.8 (C-2"), 125.3 (C-6), 122.9 (C-6), 121.8 (C-4), 120.3 (C-5"), 119.1 (C-4"), 114.1 (C-7), 113.1 (C-5), 112.6 (C-7"), 110.3 (C-3), 107.8 (C3"), 55.0 (C-2'), 54.8 (C-5'), 52.8 (C-3'a), 28.8 (C-8'), 28.4 (C-1').

IR (ATR): ṽ [cm^{-1}] = 3348 (br), 3204 (br), 2885 (br), 1724 (m), 1674 (s), 1458 (s), 1360 (m), 1232 (s), 1102 (s), 1034 (s), 882 (m), 799 (m), 743 (vs).

HRMS (ESI): m/z calc. for $C_{22}H_{19}BrN_4O_2+H^+$: 483.1026 $[M+H]^+$; found: 483.1028.

Optical Rotation: $[\alpha]_D^{20}$ = -37 (c = 0.174, MeOH).

Melting Point: 277 °C.

5-Bromo-DL-cWW-DKP (**13e**)

^{1}H-NMR (600 MHz, CD_3OD) δ 7.59 (d, $^4J_{4,6}$ = 1.9 Hz, 1H, 4-H), 7.43 (d, $^3J_{4,5}$ = 8.0 Hz, 1H, 4"-H), 7.28 (d, $^3J_{7'',6''}$ = 8.1 Hz, 1H, 7"-H), 7.18 (d, $^3J_{7,6}$ = 8.6 Hz, 1H, 7-H), 7.10 (dd, $^3J_{6,7}$ = 8.6 Hz, $^4J_{6,4}$ = 1.9 Hz, 1H, 6-H), 7.02 (t, $^3J_{6'',5'',4''}$ = 7.5 Hz, 1H, 6"-H), 6.92 (m, 3H, 2-H, 2"-H, 5"-H,), 3.49 (t, $^3J_{2',1'}$ = 4.5 Hz, 1H, 2'-H), 3.30 (d, $^3J_{5',8'}$ = 4.6 Hz, 1H, 5'-H), 3.19 (dd, $^2J_{8',8'}$ = 14.8 Hz, $^3J_{8',5'}$ = 4.6 Hz, 1H, 8'-H_a), 3.10 (dd, $^2J_{1',1'}$ = 14.9 Hz, $^2J_{1',2'}$ = 4.7 Hz, 1H, 1'-H_a), 2.98 (dd, $^2J_{8',8'}$ = 14.8 Hz, $^3J_{8',5'}$ = 4.4 Hz, 1H, 8'-H_b), 2.88 (dd, $^2J_{1',1'}$ = 14.9 Hz, $^3J_{1',2'}$ = 4.4 Hz, 1H, 1'-H_b).

^{13}C-NMR (151 MHz, CD_3OD) δ 170.7 (C-6'), 170.4 (C-3'), 137.9 (C-7"a), 136.4 (C-7a), 130.6 (C-3a), 128.8 (C-3"a), 127.0 (C-2), 125.7 (C2"), 125.3 (C-6), 122.5 (C-6"), 122.3 (C-4), 120.1 (C-5"), 119.7 (C-4"), 113.8 (C-7), 113.3 (C-5), 112.2 (C-7"), 109.1 (C-3), 109.1 (C-3"), 56.6 (C2'), 56.1 (C-5'), 30.3 (C-8'), 29.7 (C-1').

IR (ATR): ṽ [cm^{-1}] = 3241 (br), 2908 (br), 1663 (s), 1433 (m), 1315 (m), 1080 (m), 890 (m), 742 (vs), 602 (m).

HRMS (ESI): m/z calc. for $C_{22}H_{19}BrN_4O_2+H^+$: 483.1026 $[M+H]^+$; found: 483.1028.

Optical Rotation: $[\alpha]_D^{20}$ = -5 (c = 0.228, MeOH).

Melting Point: 178 °C.

Table S1: Comparison of the analytical data of the diastereomeric diketopiperazines **13a-13f**

	5-Cl-D,L **13f**		5-Br-D,L- **13e**		5-Br-L,L **13a**		5-Cl-L,L **13c**		5-Prenyl-L,L **13b***		L,L **13d****		D,L**	
**Position**	^{1}H	^{13}C	^{1}H	^{13}C	^{1}H	^{13}C	^{1}H	^{13}C	^{1}H	^{13}C	^{1}H	^{13}C	^{1}H	^{13}C
**2**	**6.96**	125.7	**6.92**	127.0	**6.42**	127.5	**6.45**	127.7	**6.46**	126.2	**6.49**	125.9	6.44	125.9
**2"**	**6.96**	127.2	**6.92**	125.7	**6.64**	125.8	**6.63**	125.6	**6.35**	125.9				
**2'**	**3.52**	56.5	**3.49**	56.6	**4.02**	56.6	**4.08**	57.0	**4.06**	56.8	**4.06**	56.9	4.03	56.9
**5'**	**3.37**	56.2	**3.30**	56.1	**4.08**	57.1	**4.03**	56.6	**4.02**	56.8				
**Optical Rotation**	**- 5°** (c = 0.415, MeOH)		**- 5°** (c = 0.228, MeOH)		**- 37°** (c = 0.174, MeOH)		**- 79°** (c = 0.192, MeOH)		**- 71°** (c = 0.17, MeOH)		**- 77°** (c = 0.40, DMSO)		**0°**	

* Synthesis of **13b** is described below. ** Values from published results by M. Haase.[24]

2.3. Enantiomerically pure substrate synthesis utilizing TrpB^{Pf0A9}

2.3.1. Formation of 5-bromo-tryptophan (12) with TrpB Pf0A9

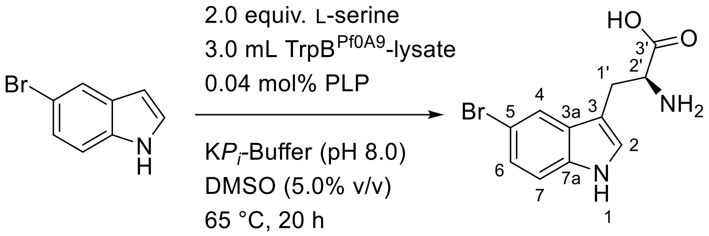

11 **12**

5-Bromoindole (0.50 g, 2.6 mmol, 1.0 equiv.) and L-serine (0.54 g, 5.1 mmol, 2.0 equiv.) were given to a 50 mL screw top bottle flask and dissolved in KP_i buffer (7.0 mL, 100 mM, pH 8.0 at 65 °C) and dimethylsulfoxide (500 µL, 5.0% (v/v)). After pyridoxal phosphate (0.24 mg, 1 µmol, 0.04 mol%) and 3.0 mL of cell lysate containing TrpB^{Pf0A9} (see 3.9 for preparation) was added, the heterogenous mixture was shaken at 65 °C and 180 rpm for 20 hours. Then, the mixture was cooled on ice for 30 minutes, following filtration of the crystallized product including a small-wash step with cooled water (2x2 mL). The crude product was then freeze-dried to give 5-bromo-L-tryptophan as a white solid.

Yield: 0.70 g, 2.5 mmol, 96%

^{1}H-NMR (600 MHz, CD_3OD) δ 7.77 (d, $^4J_{4,6}$ = 1.9 Hz, 1H, 4-H), 7.32 (d, $^3J_{7,6}$ = 8.6 Hz, 1H, 7-H), 7.27 (s, 1H, 2-H), 7.23 (dd, $^3J_{6,7}$ = 8.6 Hz, $^4J_{6,7}$ = 1.8 Hz, 1H, 6-H), 4.26 (dd, $^3J_{2',1'}$ = 7.3, 5.2 Hz, 1H, 2'-H), 3.44 (dd, $^2J_{1',1'}$ = 15.4 Hz, $^3J_{1',2'}$ = 5.2 Hz, 1H, 1'-H_a), 3.35 (dd, $^2J_{1',1'}$ = 15.4 Hz, $^3J_{1',2'}$ = 7.3 Hz, 1H, 1'-H_b).

^{13}C-NMR (151 MHz, CD_3OD) δ 171.6 (C-3'), 136.9 (C-7a), 130.2 (C-3a), 127.2 (C-2), 125.7 (C-6), 121.7 (C-4), 114.3 (C-7), 113.5 (C-5), 107.6 (C-3), 54.4 (C-2'), 27.3 (C-1').

IR (ATR): ṽ [cm^{-1}] = 2959 (w), 2931 (w), 2868 (w), 1666 (vs), 1603 (m), (1498 (s), 1414 (s), 1303 (s), 750 (m).

HRMS (ESI): m/z calc. for $C_{11}H_{12}N_2O_2Br+H^+$: 283.0077 $[M+H]^+$; found: 283.0079.

Optical Rotation: $[\alpha]_D^{20}$ = +32.5 (c = 0.04, Methanol), Lit.: $[\alpha]_D^{20}$ = +31.8 (c = 1.0, aq. HCl).[42]

The ^{1}H-NMR spectrum is consistent with the data presented in the literature. However, minor deviations related to pH-induced shifts have been observed.[42]

2.3.2. Formation of *O*-Me protected Tryptophan (S1)

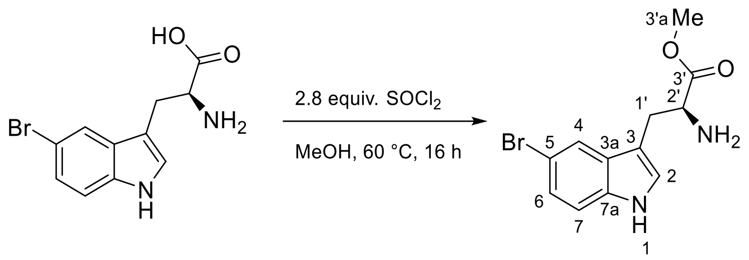

12 **S1**

5-Bromo-L-tryptophan (540 mg, 1.9 mmol, 1.0 equiv.) was given to a 100 mL heat-dried round-bottomed flask and dissolved in dried methanol (25 mL). To the solution thionyl chloride (0.38 mL, 5.3 mmol, 2.8 equiv.) was added cautiously while stirring. The solution was then heated to 60 °C for 18 hours. Then, the mixture was cooled down, diluted with ethyl acetate (25 mL) and neutralized by addition of an aqueous, saturated solution of sodium carbonate (20 mL). The organic phase was separated and the aqueous phase was extracted with ethyl acetate (3x10 mL). Afterwards, the organic phases were combined, washed with an aqueous, saturated solution of sodium chloride, dried over magnesium sulfate and the solvent removed under reduced pressure to give 5-bromo-L-tryptophan methylester (**S1**) as a white solid without further purification.

Yield: 564 mg, 1.9 mmol, quant.

^{1}H-NMR (600 MHz, CD_3OD) δ 7.64 (d, $^4J_{4,6}$ = 1.9 Hz, 1H, 4-H), 7.26 (d, $^3J_{7,6}$ = 8.6 Hz, 1H, 7-H), 7.17 (dd, $^3J_{6,7}$ = 8.6 Hz, $^4J_{6,4}$ = 1.9 Hz, 1H, 6-H), 7.12 (s, 1H, 2-H), 3.74 (t, $^3J_{2',1'}$ = 6.1 Hz, 1H, 2'-H), 3.65 (s, 3H, 3'-H_a), 3.15 – 3.06 (m, 2H, 1'-H_a, 1'-H_b).

^{13}C-NMR (151 MHz, CD_3OD) δ 175.2 (C-3'), 135.3 (C-7a), 129.2 (C-3a), 124.9 (C-2), 123.8 (C-6), 120.5 (C-4), 112.6 (C-7), 111.6 (C-5), 109.0 (C-3), 54.4 (C-2'), 51.1 (C-3'a), 29.7 (C-1').

IR (ATR): ṽ [cm^{-1}] = 3360 (w), 3295 (w), 2915 (m), 2850 (m), 1733 (vs), 1422 (s) 1334 (s), 1219 (s), 1199 (vs), 1180 (vs), 1111 (s), 1002 (vs), 866 (s), 793 (vs), 652 (s).

HRMS (ESI): m/z calc. for $C_{12}H_{13}N_2O_2Br+H^+$: 297.0233 $[M+H]^+$; found: 297.0235.

The ^{1}H- and ^{13}C-NMR spectra are consistent with the data presented in the literature. However, minor deviations related to pH-induced shifts have been observed.[43]

2.3.3. Dimerization / Formation of peptide bond towards dipeptide S2

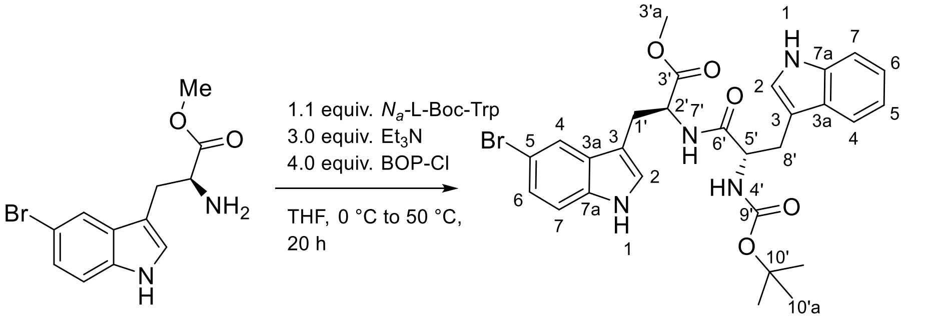

S1 **S2**

5-Bromo-L-tryptophan methyl ester (**S1**) (564 mg, 1.90 mmol, 1.0 equiv.) and N_α-(*tert.*-butoxycarbonyl)-L-tryptophan (636 mg, 2.1 mmol, 1.1 equiv.) were given to a 100 mL heat-dried round bottomed flask and dissolved in dried tetrahydrofuran (40 mL). The solution was stirred at 0 °C and subsequently triethylamine (1.0 mL, 7.6 mmol, 4 equiv.) and bis-(2-oxo-3-oxazolidinyl) phosphinic chloride (1.45 g, 5.7 mmol, 3.0 equiv.) were added. The solution was stirred for 18 hours at 23 °C, after which the temperature was raised to 50 °C for 2 hours. Then, the mixture was cooled down, diluted with ethyl acetate (40 mL) and neutralized by addition of an aqueous, saturated solution of ammonium chloride (20 mL). The organic phase was separated and the aqueous phase was extracted with ethyl acetate (5x15 mL). Afterwards, the organic phases were combined, washed with an aqueous, saturated solution of sodium chloride, dried over magnesium sulfate and the solvent removed under reduced pressure. After chromatographic purification (2/1 petroleumether/ethyl acetate) methyl (2S)-3-(5-bromo-1*H*-indol-3-yl)-2-(2-((*tert.*-butoxycarbonyl)amino)-3-(1*H*-indol-3-yl)propanamido)propanoate (**S2**) was obtained as white crystals.

Yield: 942.3 mg, 1.61 mmol, 85%.

^{1}H-NMR (600 MHz, CD_3OD) δ 7.57 (d, $^3J_{4'',5''}$ = 8.1 Hz, 1H, 4''-H), 7.55 (s, 1H, 4-H), 7.32 (d, $^3J_{7'',6''}$ = 8.1 Hz, 1H, 7''-H), 7.24 (d, $^3J_{7,6}$ = 8.4 Hz, 1H, 7-H), 7.17 (d, $^3J_{6,7}$ = 8.7 Hz, 2H, 6-H), 7.13 – 7.06 (m, 2H, 6''-H, 2''-H), 7.05 – 6.98 (m, 2H, 5''-H, 2-H), 4.66 (t, $^3J_{5',8'}$ = 6.2 Hz, 1H, 5'-H), 4.36 – 4.31 (m, 1H, 2'-H), 3.58 (s, 3H, 3'-H$_a$), 3.18 (dd, $^2J_{8',8'}$ = 14.7 Hz, $^3J_{8',5'}$ = 5.7 Hz, 1H, 8'-H$_a$), 3.11 (d, $^3J_{1',2'}$ = 6.4 Hz, 1H, 1'-H$_a$), 3.03 (dd, $^2J_{8',8'}$ = 14.8 Hz, $^3J_{8',5'}$ = 7.7 Hz, 1H, 8'-H$_b$), 1.34 (s, 9H, 10'a-H), 1.18 – 1.11 (m, 1H, 1'-H$_b$).

^{1}H-NMR (151 MHz, CD_3OD) δ 174.5 (C-6'), 173.3 (C-3'), 157.5 (C-9'), 138.1 (C-7''a), 136.6 (C-7a), 130.5 (C-3a), 128.8 (C-3''a), 126.3 (C-2), 125.2 (C-6), 124.7 (C-2''), 122.4 (C-6''), 121.8 (C-4), 119.8 (C-5''), 119.4 (C-4''), 114.0 (C-7), 113.1 (C-5), 112.4 (C-3''), 112.3 (C-7''), 110.1 (C-3), 80.7 (C-10'), 56.8 (C-2'), 54.5 (C-5'), 52.7 (C-3'a), 29.1 (C-8'), 28.6 (C-10'a), 28.2 (C-1').

IR (ATR): ṽ [cm^{-1}] = 3321 (br), 2978 (w), 1704 (vs), 1664 (s), 1504 (s), 1457 (s), 1436 (s), 1364 (vs), 1222 (vs), 1164 (vs), 1099 (m), 743 (vs) 530 (vs).

HRMS (ESI): m/z calc. for $C_{28}H_{32}N_4O_5Br+H^+$: 583.1551 $[M+H]^+$; found: 583.1555.

2.3.4. *N*-Deprotection towards Amine S3

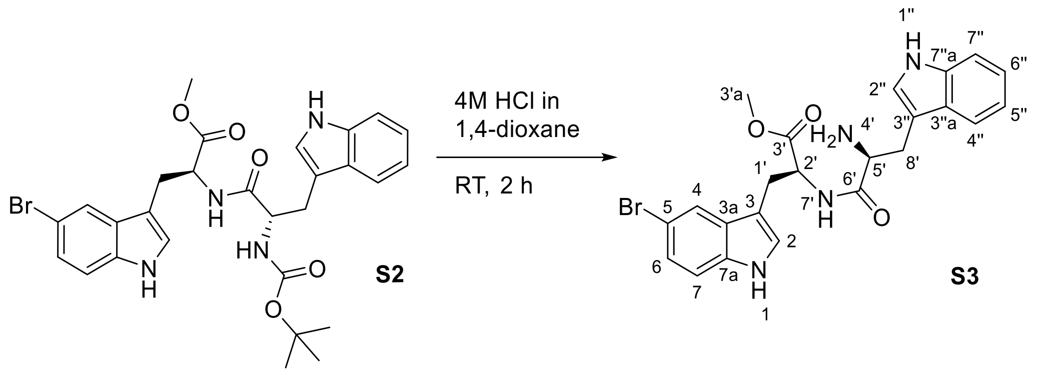

Dipeptide **S2** (830 mg, 1.42 mmol, 1 equiv.) was dissolved in THF (2 mL) and hydrogen chloride (4M in 1,4-dioxan, 5 mL) and stirred for 2 hours. Then, the solvent was removed under reduced pressure to give methyl (2S)-2-(5-amino-3-(1*H*-indol-3-yl)propanamido)-3-(5-bromo-1*H*-indol-3-yl)propanoate) (**S3**) without further purification.

Yield: 646 mg, 1.4 mmol, quant.

Crude ^{1}H-NMR (600 MHz, CD_3OD) δ 7.66 (app. dd, *J* = 8.6, 1.4 Hz, 2H, 4-H, 4”-H), 7.38 (app. dd, $^3J_{7'',6''}$ = 8.1 Hz, $^4J_{7'',5''}$ = 0.9 Hz, 1H, 7“-H), 7.27 (d, $^3J_{7,6}$ = 8.6 Hz, 1H, 7-H), 7.21 (s, 1H, 2“-H), 7.19 (dd, $^3J_{6,7}$ = 8.6 Hz, $^4J_{6,4}$ = 1.9 Hz, 1H, 6-H), 7.14 (dd, $^3J_{6'',7''}$ = 8.1 Hz, $^3J_{6'',5''}$ = 7.0 Hz, $^4J_{6'',4''}$ = 1.1 Hz, 1H, 6“-H), 7.11 (s, 1H, 2-H), 7.05 (ddd, $^3J_{5'',4''}$ = 8.0 Hz, $^3J_{5'',6''}$ = 7.0 Hz, $^4J_{5'',7''}$ = 1.0 Hz, 1H, 5“-H), 4.75 (dd, $^3J_{2',1'}$ = 7.5, 6.1 Hz, 1H, 2‘-H), 4.14 (dd, $^3J_{5',8'}$ = 8.4, 5.9 Hz, 1H, 5‘-H), 3.65 (s, 3H, 3’-H_a), 3.41 (dd, $^2J_{8',8'}$ = 15.0 Hz, $^3J_{8',5'}$ = 5.8 Hz, $^4J_{8',2''}$ = 1.0 Hz, 1H, 8’-H_a), 3.27 (dd, $^2J_{1',1'}$ = 14.7 Hz, $^3J_{1',2'}$ = 6.1 Hz, $^4J_{1',2}$ = 0.8 Hz 1H, 1’-H_a), 3.19 (app. td, *J* = 14.6, 8.0 Hz, 2H, 1’-H_b, 8’-H_a).

Crude ^{13}C-NMR (151 MHz, CD_3OD) δ 173.1 (C-3‘), 170.0 (C-6‘), 138.3 (C-7“a), 136.7 (C-7a), 130.4 (C-3a), 128.3 (C-3”a), 126.2 (C-2), 125.8 (C-2”), 125.3 (C-6), 122.9 (C-6), 121.8 (C-4), 120.3 (C-5”), 119.1 (C-4”), 114.1 (C-7), 113.1 (C-5), 112.6 (C-7”), 110.3 (C-3), 107.8 (C3”), 55.0 (C-2’), 54.8 (C-5’), 52.8 (C-3’a), 28.8 (C-8’), 28.4 (C-1’).

IR (ATR): ṽ [cm^{-1}] = 3348 (br), 3204 (br), 2885 (br), 1724 (m), 1674 (s), 1458 (s), 1360 (m), 1232 (s), 1102 (s), 1034 (s), 882 (m), 799 (m), 743 (vs).

HRMS (ESI): m/z calc. for $C_{23}H_{24}N_4O_3Br+H^+$: 483.1026 $[M+H]^+$; found: 483.1028.

2.3.5. *N*-Deprotection and cWW-DKP-Formation (13a)

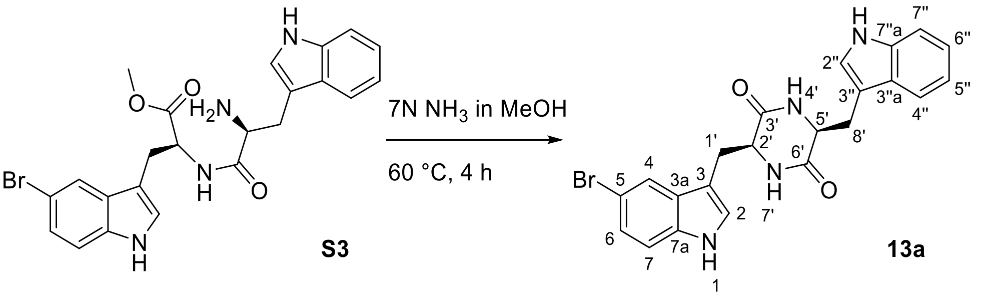

Amine **S3** was dissolved in NH_3 (7N in methanol, 8 mL) and stirred at 60 °C for 4 hours. Then, the solvent was removed under reduced pressure. After chromatographic purification (97/3 ethyl acetate/methanol) the 5-bromo-cWW-DKP (**13a**) was obtained as white crystals.

Yield: 601 mg, 1.3 mmol, 93%.

^{1}H-NMR (600 MHz, CD_3OD) δ 7.68 (d, $^{4}J_{4,6}$ = 1.9 Hz, 1H, 4-H), 7.44 (d, $^{3}J_{4'',5''}$ = 8.0 Hz, 1H, 4“-H), 7.32 (d, $^{3}J_{7'',6''}$ = 8.2 Hz, 1H, 7“-H), 7.23 (d, $^{3}J_{7,6}$ = 8.6 Hz, 1H, 7-H), 7.19 (dd, $^{3}J_{6,7}$ = 8.6 Hz, $^{4}J_{6,7}$ 1.8 Hz, 1H, 6-H), 7.11 (t, $^{3}J_{6'','' 5,7''}$ = 7.6 Hz, 1H, 6“-H), 7.03 (t, $^{3}J_{5'',4'',6''}$ = 7.5 Hz, 1H, 5“-H), 6.64 (s, 1H, 2“-H), 6.42 (s, 1H, 2-H), 4.08 (dd, $^{3}J_{5',8'}$ = 7.0, $^{3}J_{5',8'}$ = 4.0 Hz, 1H, 5‘-H), 4.02 (dd, $^{3}J_{2',1'}$ = 7.5, $^{3}J_{2',1'}$ = 3.9 Hz, 1H, 2‘-H), 2.97 (dd, $^{2}J_{8',8'}$ = 14.4 Hz, $^{3}J_{8',5'}$ = 3.9 Hz, 1H, 8’-H_a), 2.84 (dd, $^{2}J_{1',1'}$ = 14.5 Hz, $^{3}J_{1',2'}$ = 4.0 Hz, 1H, 1’-H_a), 2.29 (dd, $^{3}J_{8',8'}$ = 14.4 Hz, $^{3}J_{8',5'}$ = 7.0 Hz, 1H, 8’-H_b), 2.03 (dd, $^{2}J_{1',1'}$ = 12.6 Hz, $^{3}J_{1',2'}$ = 6.3 Hz, 1H, 1’-H_b).

^{13}C-NMR (151 MHz, CD_3OD) δ 169.7 (C-3‘/6‘), 169.7 (C-6‘/3‘), 138.1 (C-7“a), 136.7 (C-7a), 130.4 (C-3a), 128.7 (C-3”a), 127.5 (C-2), 125.8 (C-2”), 125.3 (C-6), 122.6 (C-4), 122.2 (C-6”), 120.2 (C-5”), 119.7 (C-4”), 114.1 (C-7), 113.3 (C-5), 112.5 (C-7”), 109.6 (C-3”), 109.4 (C-3), 57.1 (C-5’), 56.6 (C-2’), 31.4 (C-8’), 31.3 (C-1’).

IR (ATR): ṽ [cm^{-1}] = 3424 (w), 2181 (w), 2037 (w), 1661 (vs), 1456 (s), 1338 (m), 1092 (s), 867 (m), 799 (s), 743 (vs), 584 (s).

HRMS (ESI): m/z calc. for $C_{22}H_{20}N_4O_2Br+H^+$: 451.0764 $[M+H]^+$; found: 451.0768.

Optical Rotation: $[\alpha]_D^{20}$ = -37 (c = 0.174, MeOH)

Melting Point: 277,1 °C.

2.4. Suzuki-Coupling

2.4.1. Preparation of Prenyl-pinacolborane (14)

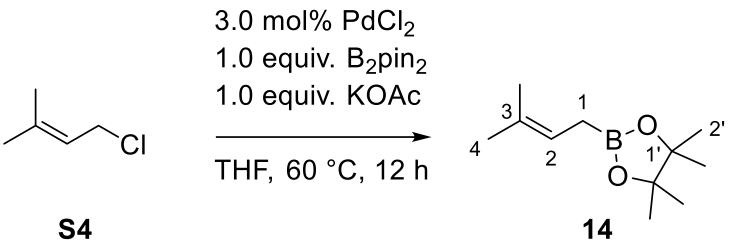

S4 **14**

Bis(pinacolato)diboron (4.5 g, 17.8 mmol, 1.0 equiv), palladium dichloride (94.4 mg, 0.53 mmol, 3.0 mol%) and potassium acetate (1.7 g, 17.8 mmol, 1 equiv.) were added to a 100 mL heat-dried round-bottomed flask and dissolved in dry THF (20 mL). 3-Methyl-2-butenylchloride (2.0 mL, 17.5 mmol, 1.0 equiv.) was added and the mixture stirred at 60 °C for 16 hours. Then, the mixture was cooled down, filtered through a pad of silica (the flask and the pad was rinsed with ethyl acetate) and the solvent removed und reduced pressure. After purification via distillation und vacuum (1-2 mbar, 60-80 °C), (*E*)-4,4,5,5-tetramethyl-2-(3-methylbut-1-en-1-yl)-1,3,2-dioxaborolane was obtained as a colourless liquid.

Yield: 3.0 g, 15.5 mmol, 87 %

^{1}H-NMR (600 MHz, $CDCl_3$) δ 5.34 – 5.15 (m, 1H,), 1.69 (m, 3H), 1.63 – 1.57 (m, 5H), 1.24 (s, 12H).

^{13}C-NMR (151 MHz, $CDCl_3$) δ 131.6, 118.6, 83.2, 25.8, 24.9, 24.7.

HRMS (ESI): m/z calc. for $C_{11}H_{21}BO_2$-H^+: 195.1551 $[M-H]^+$; found: 195.1549.

The ^{1}H- and ^{13}C-NMR spectra are consistent with the data presented in the literature.[40]

2.4.2. Screening of different catalyst-system

To attach the prenylgroup in 5-position, different catalyst/ligand-systems were tested on Boc-protected 5-bromoindole (**S5**) according to Yang et al.[34]

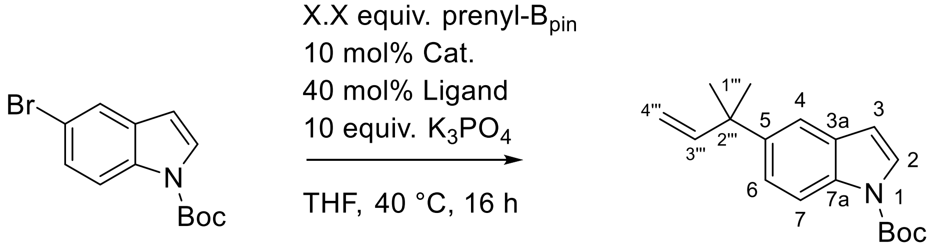

S5 **S6**

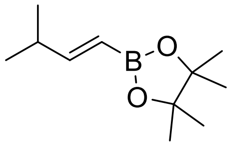

14
Prenyl-B_{pin}

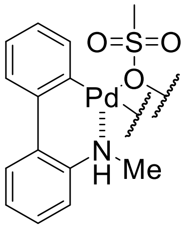

S7
Buchwald-Hartwig-Precat.-Dimer

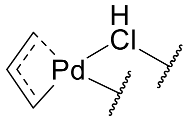

S8
Allylpalladium chloride dimer

NOM-Phos

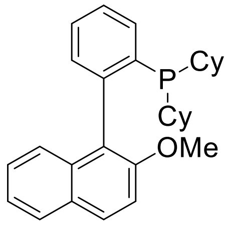

15
dicyclohexyl (2-(2-methoxynaphthalen-1-yl) phenyl)phosphane

RuPhos

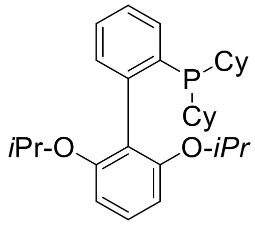

S9

SPhos

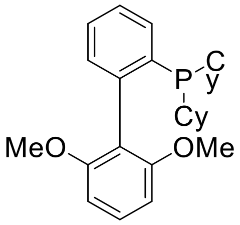

16

Catalyst (10 mol%), ligand (40mol%) and potassium carbonate (1.3 mL of a 2.5 M solution in oxygen-free water, 3.38 mmol, 10 equiv) were given to a Schlenk flask under argon. Prenylpincacolborane (79 mg, 0.41 mmol, 1.2 equiv. / 331 mg, 1.7 mmol, 5.0 equiv.) and tert-butyl 5 bromo-1H-indole-1-carboxylate (100 mg, 0.34 mmol,1.0 equiv) were both dissolved in oxygen-free THF (3 mL each, freeze-pump-thawed) and added to the reaction flask. The mixture was stirred at 40 °C for 16 hours. Then, the mixture was cooled down, diluted with ethyl acetate (10 mL) and neutralized by addition of an aqueous, saturated sodium chloride solution (5 mL). The organic phase was separated and the aqueous phase was extracted with ethyl acetate (3x5 mL). Afterwards, the organic phases were combined, washed with an aqueous, saturated solution of sodium chloride, dried over magnesium sulfate and the solvent removed under reduced pressure. The crude mixture was analyzed with regard to the product content via qNMR in duplicates with trimethoxybenzene as the internal standard.

Table S2: Screened catalyst and ligand combinations for the reverse-prenylation

	Eq. Bpin	Catalyst	Ligand	Yield* [%]
**1**	1.2	$[Allyl\text{-}PdCl]_2$	*NOM*-Phos	52.6 ± 3.9
**2**	5.0	$[Allyl\text{-}PdCl]_2$	*NOM*-Phos	59.2 ± 1.6
**3**	1.2	BH-Precat.-Dimer	*NOM*-Phos	60.9 ± 1.2
**4**	1.2	SPhos G4	SPhos	64.4 ± 6.8
**5**	1.2	RuPhos G4	RuPhos	57.7 ± 4.0

Yield measured via qNMR in duplicates with Trimethoxybenzene as the internal standard

For the entry with the highest detected yield (Entry 4, SPhos, SPhos Pd G4) the crude product was purified over a short pad of silica (40/1 petroleumether/ethyl acetate) to remove major impurities and to give *tert*.-butyl 5-(2-methylbut-3-en-2-yl)-1H-indole-1-carboxylate (**S6**).

Yield (isolated only for Entry 4): 69 mg, 0.24 mmol, 71%

Crude ^{1}H-NMR (600 MHz, $CDCl_3$) δ 8.08 – 8.00 (m, 1H), 7.58 (d, *J* = 3.8 Hz, 1H, 2-H), 7.54 (d, *J* = 1.9 Hz, 1H), 7.33 (dd, *J* = 8.7, 2.0 Hz, 1H), 6.54 (d, *J* = 3.7 Hz, 1H), 6.09 (dd, *J* = 17.4, 10.6 Hz, 1H), 5.07 (dd, *J* = 27.7, 1.4 Hz, 1H), 5.06 (d, *J* = 1.4 Hz, 1H), 1.67 (s, 9H), 1.46 (s, 6H).

The 1H-NMR spectrum is consistent with the data presented in the literature[34], and therefore no further characterization of the product was deemed necessary.

2.4.3. Suzuki-Coupling to form 5-prenyl-cWW-DKP (13b)

According to the results of the catalyst/ligand screening, the prenylation of 5-bromo-cWW-DKP was tested as well with two cat.-systems: SPhos/SPhos Pd G4 and *NOM-Phos*/allylpalladium chloride.

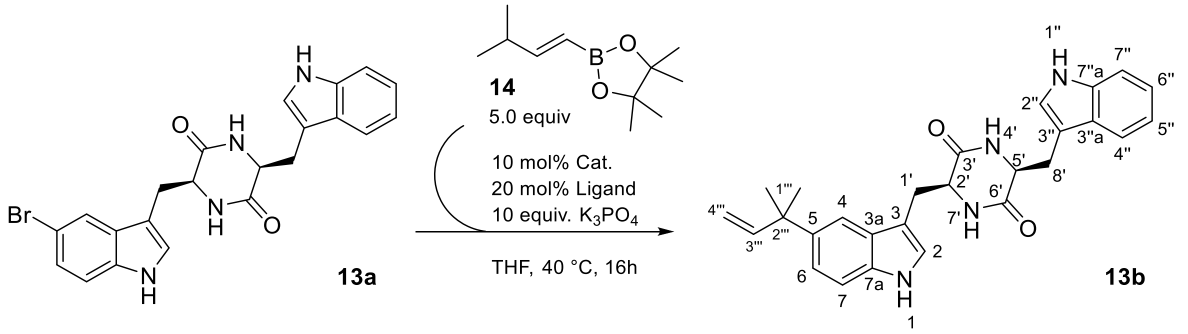

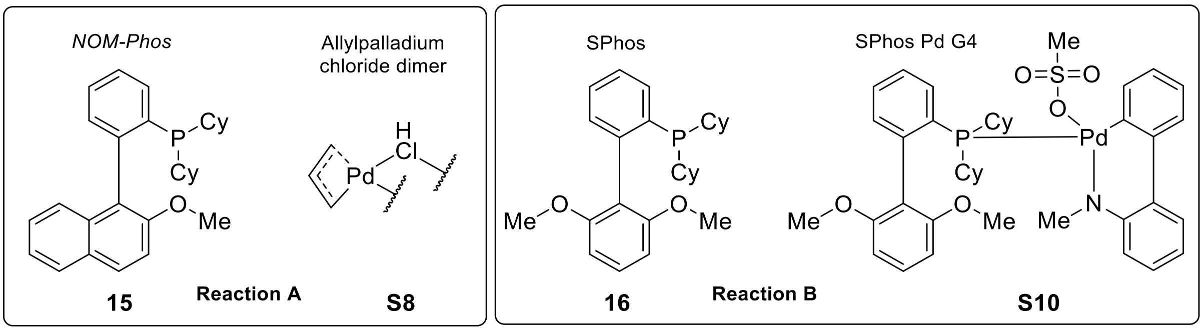

Reaction A: Allylpalladium chloride (4.0 mg, 11 µmol, 10 mol%), *NOM-Phos* (9.5 mg, 22 µmol, 20 mol%) and potassium carbonate (233 mg, 1.1 mmol, 10 equiv.) were given to a Schlenk flask under argon.

Reaction B: SPhos Pd G4 (8.7 mg, 11 µmol, 10 mol%), SPhos (9.0 mg, 22 µmol, 20 mol%) and potassium carbonate (233 mg, 1.1 mmol, 10 equiv.) were given to a Schlenk flask under argon.

For both reactions afterwards: Prenylpinacolborane (108 mg, 0.55 mmol, 5.0 equiv.) and 5-bromo-cWW-DKP (**13a**) (50 mg, 0.11 mmol, 1.0 equiv.) were both dissolved in oxygen-free-THF (2 mL each, freeze-pump-thawed) and added to the reaction flask. Oxygen-free water (2 mL, freeze-pump-thawed) was added and the mixture stirred at 40 °C for 16 hours. Then, the mixture was cooled down, diluted with ethyl acetate (10 mL) and neutralized by addition of an aqueous, saturated sodium chloride solution (5 mL). The organic phase was separated and the aqueous phase was extracted with ethyl acetate (3x5 mL). Afterwards, the organic phases were combined, washed with an aqueous, saturated solution of sodium chloride, dried over magnesium sulfate and the solvent removed under reduced pressure. After chromatographic purification (1/1 petroleumether/ethyl acetate) 5-prenyl-cWW-DKP (**13b**) was obtained as a white powder.

Yield Reaction A: 35.0 mg, 0.80 mmol, 72%. **Yield Reaction B:** 39.9 mg, 0.90mmol, 82%

^{1}H-NMR (600 MHz, CD_3OD) δ 7.57 (d, $^{4}J_{4,6}$ = 1.8 Hz, 1H, 4-H), 7.46 (dd, $^{3}J_{4'',5''}$ = 8.0 Hz, $^{4}J_{4'',6''}$ = 1.0 Hz, 1H, 4”-H), 7.30 (dd, $^{3}J_{7'',6'}$ = 8.1 Hz, $^{4}J_{7'',5''}$ = 0.9 Hz, 1H, 7“-H), 7.23 (dd, $^{3}J_{7,6}$ = 8.6 Hz, $^{4}J_{7,5}$ = 0.7 Hz, 1H, 7-H), 7.14 (dd, $^{3}J_{6,7}$ = 8.6 Hz, $^{4}J_{6,4}$ = 1.8 Hz, 1H, 6-H), 7.08 (ddd, $^{3}J_{6'',5''}$ = 8.2 Hz, $^{3}J_{6'',7''}$ = 7.0, $^{4}J_{6'',4''}$ = 1.2 Hz, 1H, 6”-H), 7.00 (ddd, $^{3}J_{5'',4''}$ = 8.0 Hz, $^{3}J_{5'',6''}$ = 7.0 Hz, $^{4}J_{5'',7''}$ = 1.0 Hz, 1H, 5”-H), 6.46 (s, 1H, 2-H), 6.34 (s, 1H, 2“-H), 6.12 (dd, $^{3}J_{3''',4'''}$ = 17.4, 10.6 Hz, 1H, 3‘‘‘-H), 5.06 (dd, $^{3}J_{4'''a,3'''}$ = 17.5 Hz, $^{3}J_{4'''a,4'''b}$ = 1.6 Hz, 1H, 4‘‘‘a-H), 5.00 (dd, $^{3}J_{4'''b,3'''}$ = 10.6 Hz,

$^{4}J_{4'''b,4'''a}$ = 1.5 Hz, 1H, 4‘‘‘b-H),4.06 (ddd, $^{3}J_{2',1'}$ = 6.9 Hz, $^{3}J_{2',1'}$ = 4.0, $^{3}J_{2',NH}$ = 1.1 Hz, 1H, 2'-H), 4.02 (ddd, $^{3}J_{5',8'}$ = 7.9 Hz, $^{3}J_{5',8'}$ = 3.8 Hz, $^{3}J_{2',NH}$ = 1.1 Hz, 1H, 5'-H), 2.94 (dd, $^{2}J_{1',1'}$ = 14.4 Hz, $^{3}J_{1',2'}$ = 3.9 Hz, 1H, 1‘-H$_a$), 2.90 (dd, $^{2}J_{8',8'}$ = 14.4 Hz, $^{3}J_{8',5'}$ = 3.8 Hz, 1H, 8‘-H$_b$), 2.32 (dd, $^{2}J_{1',1'}$ = 14.4 Hz, $^{3}J_{1',2'}$ = 6.9 Hz, 1H, 1‘-H$_b$), 1.99 (dd, $^{2}J_{8',8'}$ = 14.4, $^{3}J_{8',5'}$ = 7.8 Hz, 1H, 8'-H$_a$), 1.47 (s, 3H, 1‘‘‘a-H), 1.46 (s, 3H, 1‘‘‘b-H).

^{13}C-NMR (151 MHz, CD_3OD) δ 169.8 (C-3‘/6‘), 169.7 (C-6‘/C-3‘), 150.6 (C-4‘‘‘), 140.5 (C-5), 138.2 (C-7”a), 136.4 (C-7a), 128.5 (C-3”a), 128.4 (C-3a), 126.2 (C-2), 125.9 (C-2“), 122.5 (C-6), 121.9 (C-6“), 120.0 (C-5“), 119.6 (C-4“), 116.5 (C-4), 112.4 (C-7“), 111.9 (C-7), 110.3 (C-3‘‘‘), 109.5 (C-2“), 109.5 (C-2), 56.8 (C-2‘/5‘), 56.8 (C-5‘/2‘), 42.1 (C-2‘‘‘), 31.5 (C-1‘/8‘), 31.4 (C-8‘/1‘), 29.4 (C-1‘‘‘).

IR (ATR): ṽ [cm^{-1}] = 3287 (br), 2961 (w), (2923 (m), 2862 (w), 1709 (m), 1656 (vs), 1541 (s), 1383 (m), 1330 (m), 1246 (m), 738 (s).

HRMS (ESI): m/z calc. for $C_{27}H_{28}N_4O_2+H^+$: 441.2285 $[M+H]^+$; found: 441.2281.

Optical Rotation: $[\alpha]_D^{20}$ = -71 (c = 0.17, MeOH).

2.4.4. Enzymatic conversion of 5-bromo-cWW-DKP (13a) in preparative scale to pyrroloindole 17a and 18a

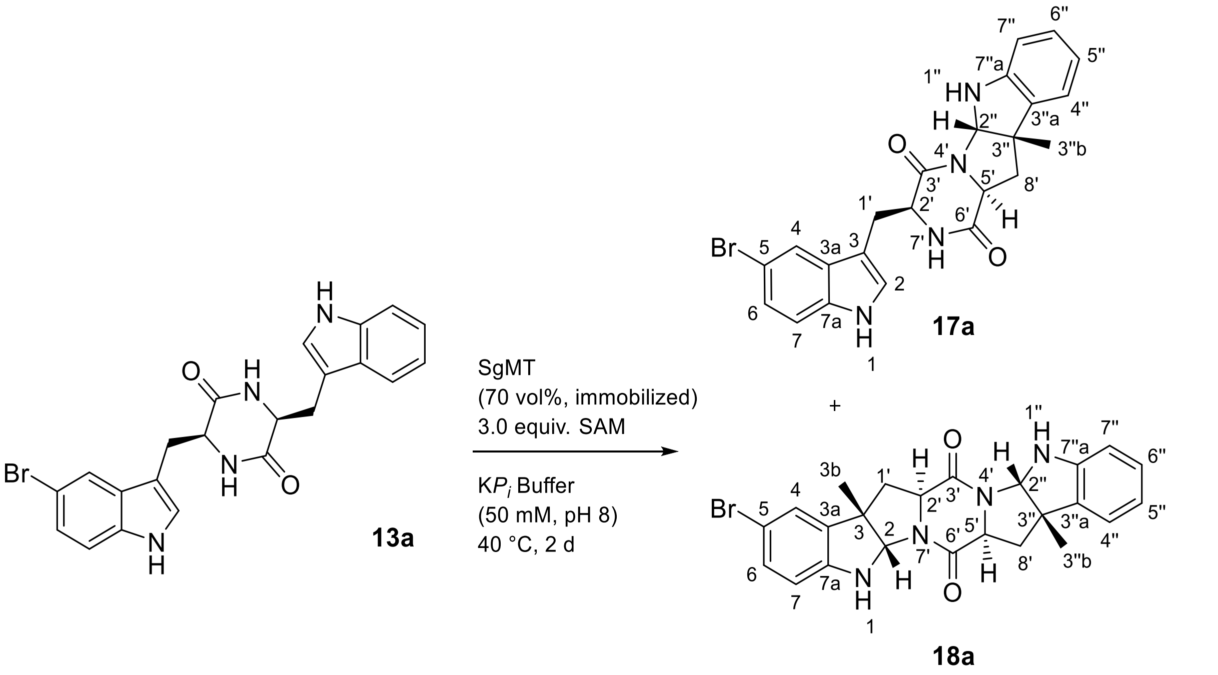

5-Br-cWW-DKP (**13a**) (50 mg, 0.11 mmol, 1.0 equiv., dissolved in 0.5 mL DMSO) and *S*-adenosylmethionine disulfate tosylate (254 mg, 0.33 mmol, 3.0 equiv.) were given to a glass bottle. The Ni-NTA-bound enzyme (70 vol%) was added (see 3.7 for preparation) and the reaction volume adjusted to 50 mL with KP_i buffer (50 mM, pH 8). The heterogenous mixture was shaked gently at 40 °C and 300 rpm for 48 hours. Then, the Ni-NTA resin was removed by filtration and the product extracted with ethyl acetate (3x15 mL). Afterwards, the organic phases were combined, washed with an aqueous, saturated solution of sodium chloride, dried over magnesium sulfate and the solvent removed under reduced pressure. After chromatographic purification (ethyl acetate) the dimethylated **17a** and monomethylated product **18a** were obtained separately as a white powder.

Yield: 41.6 mg, 87 µmol, 79% (**18a**) and 4.0 mg, 6.5 µmol, 6% (**17a**)

Monomethylated Product – 5-Br-L,L-MoMe-cWW-DKP (**17a**)

^{1}H-NMR (600 MHz, CD_3OD) δ 7.78 (d, $^4J_{4,6}$ = 1.9 Hz, 1H, 4-H), 7.30 (d, $^3J_{7,6}$ = 8.6 Hz, 1H, 7-H), 7.19 (dd, $^3J_{6,7}$ = 8.6 Hz, $^4J_{6,4}$ = 1.9 Hz, 1H, 6-H), 7.11 (s, 1H, 2-H), 6.95 (td, $^3J_{6'',5'',7''}$ = 7.6 Hz, $^4J_{6'',4''}$ = 1.3 Hz, 1H, 6“-H), 6.91 (d, $^3J_{4'',5''}$ = 7.4 Hz, 1H, 4“-H), 6.63 (td, $^3J_{5'',6'',4''}$ = 7.4 Hz, $^4J_{5'',7''}$ = 1.0 Hz, 1H, 5”-H, 6.52 (d, $^3J_{7'',6''}$ = 7.8 Hz, 1H, 7“-H), 4.92 (s, 1H, 2“-H), 4.36 (dd, $^3J_{2',1'}$ = 5.2, $^3J_{2',1'}$ = 2.8 Hz, 1H, 2‘-H), 3.67 (ddd, $^2J_{5',8'}$ = 12.2 Hz, $^3J_{5',8'}$ = 5.2, $^3J_{5',2''}$ = 1.8 Hz, 1H, 5‘-H), 3.49 (dd, $^2J_{1',1'}$ = 14.6 Hz, $^3J_{1',2'}$ = 3.5 Hz, 1H, 1’-H_b), 3.10 (dd, $^2J_{1',1'}$ = 14.6 Hz, $^3J_{1',2'}$ = 4.5 Hz, 1H, 1’-H_a), 2.10 (dd, $^2J_{8',8'}$ = 11.8 Hz, $^3J_{8',5'}$ = 5.2 Hz, 1H, 8’-H_a), 0.82 (s, 3H, 3“b-H), 0.53 (t, $J_{8',8',5'}$ = 12.0 Hz, 1H, 8‘-H_b).

^{13}C-NMR (151 MHz, CD_3OD) δ 169.6 (C-2‘), 167.0 (C-6‘), 150.9 (C-7“a), 136.4 (C-7a), 133.5 (C-3”a), 130.5 (C-3a), 129.5 (C-6”), 127.9 (C-2), 125.4 (C-6), 123.7 (C-4”), 122.8 (C-4), 120.1 (C-5”), 114.1 (C-7), 113.4 (C-5), 109.8 (C-7”), 108.9 (C-3), 82.4 (C-2”), 59.3 (C-5’), 57.4 (C-2’), 52.6 (C-3”), 43.6 (C-8’), 30.6 (C-1’), 25.0 (C-3”b).

HRMS (ESI): m/z calc. for $C_{23}H_{22}N_4O_2Br+H^+$: 465.0921 $[M+H]^+$; found: 465.0914.

IR (ATR): ṽ [cm^{-1}] = 3279 (br), 2924 (m), 2854 (m), 1661 (vs), 1609 (m), 1484 (m), 1455 (s), 1312 (m), 1214 (m), 1066 (s), 881 (m), 795 (m), 747 (s), 606 (m).

Optical Rotation: $[\alpha]_D^{20}$ = -180 (c = 0.31, $CHCl_3$)

Dimethylated Product – 5-Br-L,L-DiMe-cWW-DKP (**18a**)

^{1}H-NMR (600 MHz, CD_3OD) δ 7.25 (d, $^3J_{4,6}$ = 2.0 Hz, 1H, 4-H), 7.12 – 7.08 (m, 2H, 4''-H, 6-H), 7.00 (t, $^3J_{6'',5'',7''}$ = 7.6 Hz, 1H, 6''-H), 6.71 (t, $^3J_{5'',4'',6''}$ = 7.4 Hz, 1H, 5''-H), 6.56 (d, $^3J_{7'',6''}$ = 7.8 Hz, 1H, 7''-H), 6.47 (d, $^3J_{7,6}$ = 8.3 Hz, 1H, 7-H), 5.23 (s, 1H, 2-H), 5.21 (s, 1H, 2''-H), 4.08 – 3.99 (m, 2H, 2'-H, 5'-H), 2.65 (app. dt, *J* = 13.4, 6.8 Hz, 2H, 1'-H,8'-H), 2.28 – 2.19 (m, 2H, 1'-H, 8'-H), 1.42 (s, 3H, 3b-H/3''b-H), 1.41 (s, 3H, 3''b-H/3b-H).

^{13}C-NMR (151 MHz, CD_3OD) δ 168.1 (C-3'/6'), 168.0 (C-6'/3'), 150.3 (C-7''a), 149.7 (C-7a), 136.5 (C-3a), 133.9 (C-3''a), 132.3 (C-6), 129.6 (C-6''), 126.9 (C-4), 123.7 (C-4''), 120.3 (C-5''), 111.8 (C-7), 111.3 (C-5), 110.5 (C-7''), 82.5 (C-2/2''), 82.4 (C-2''/2), 61.4 (C-2'/5'), 61.3 (C-5'/2'), 53.1 (C-3/3''), 53.0 (C-3''/3), 41.6 (C-1'/8'), 41.5 (C-8'/1'), 25.0 (C-3b/3''b), 25.0 (C-3''b/3b).

HRMS (ESI): m/z calc. for $C_{24}H_{24}N_4O_2Br+H^+$: 479.1077 $[M+H]^+$; found: 479.1074.

IR (ATR): ṽ [cm^{-1}] = 3348 (br), 2971 (w), 2958 (m), 2925 (m), 2850 (w), 1661 (vs), 1600 (m), 1479 (s), 1427 (s), 1216 (m), 1190 (m), 1165 (m), 1057 (m), 813 (w), 753 (w).

Optical Rotation: $[\alpha]_D^{20}$ = -494 (c = 0.174, $CHCl_3$)

2.5. Screening for *N*-Methylation-Conditions

Regarding the screening for suitable *N*-methylation conditions, Eschweiler-Clarke conditions were tested first. As a model substrate compound **S11** was used initially, since it contained the hexahydropyrroloindole motif and a prenyl group and was synthetically available in high amounts.

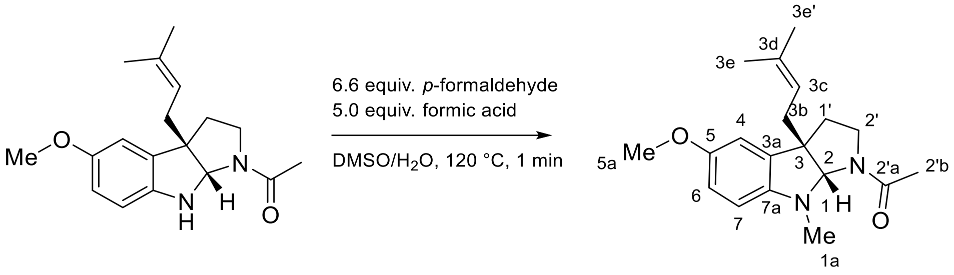

S11 **S12**

The pyrroloindole **S11** (9.0 mg, 30 µmol, 1.0 equiv.) was dissolved in DMSO in a microwave flask. *p*-Formaldehyde (4.3 µL, 0.20 mmol, 6.66 equiv.) and formic acid (5.6 µL, 0.15 mmol, 5.0 equiv.) was added afterwards and the mixture stirred under microwave irradiation (120 W) maintaining a temperature of 120 °C. Then, the mixture was cooled down, diluted with dichloromethane (3 mL) and neutralized by addition of an aqueous, saturated sodium chloride solution (2 mL). The organic phase was separated and the aqueous phase was extracted with ethyl acetate (3x5 mL). Afterwards, the organic phases were combined, washed with an aqueous, saturated solution of sodium chloride, dried over magnesium sulfate and the solvent removed under reduced pressure. After filtration over a pad of silica (1/1 petroleumether/ethyl acetate) major impurities were removed and the crude *N*-methylated product **S12** was obtained.

Yield: 5.1 mg, 16 µmol, 54%.

Crude ^{1}H-NMR (300 MHz, CD_3OD) δ 6.70 (d, $^4J_{4,6}$ = 2.6 Hz, 1H, 4-H), 6.68 (dd, $^3J_{6,7}$ = 8.4, $^4J_{6,4}$ 2.6 Hz, 1H, 6-H), 6.37 (d, $^3J_{7,6}$ = 8.4 Hz, 1H, 7-H), 5.31 (s, 1H, 2-H), 5.03 (ddd, $^3J_{3c,3b}$ = 9.1 Hz, $^3J_{3c,3b}$ = 5.2 Hz, $^3J_{3c,3e/3e'}$ = 1.5 Hz, 1H, 3c-H), 3.72 (s, 3H, 5a-H), 3.23 (app. ddd, *J* = 10.6 Hz, 9.2 Hz, 7.2 Hz, 1H, 2'-H), 2.89 (s, 3H, 1a-H), 2.43 (d, $^3J_{3b,3c}$ = 7.7 Hz, 2H, 3b-H), 2.13 (app. dd, *J* = 6.6, 3.2 Hz, 2H, 1'-H), 2.08 (s, 3H, 2'b-H), 1.66 (d, $^4J_{3e,3c}$ = 1.5 Hz, 3H, 3e'-H), 1.57 (d, $^4J_{3e,3c}$ = 1.4 Hz, 3H, 3e-H).

Crude ^{13}C-NMR (76 MHz, CD_3OD) δ 172.8 (C-2'a), 154.8 (C-5), 147.0 (C-7a), 135.7 (C-3d), 135.5 (C-3a), 120.8 (C-3c), 114.3 (C-6), 111.0 (C-4), 108.5 (C-7), 86.9 (C-2), 57.3 (C-3), 56.4 (C-5a), 48 (C-2'), 38.1 (C-1'), 37.3 (C-3b), 35.5 (C-1a), 26.1 (C-3e), 22.6 (C-2'b), 18.1 (C-3e').

IR (ATR): ṽ [cm^{-1}] = 2932 (br), 1649 (vs), 1496 (vs), 1407 (vs), 1277 (m), 1216 (m), 1031 (s), 857 (w), 803 (w).

MS (APCI): m/z calc. for $C_{19}H_{26}N_2O_2+H^+$: 315.2 [M+H]$^+$; found: 315.1.

The identified reaction conditions were then transferred to a substrate, that is even more closely aligned with the natural product, namely the unsubstituted cWW-DKP **S10**, which is the natural substrate of the methyltransferase. As such, it is converted towards the double C3-methylated product in high yields with minimal amounts of enzyme lysate and was already described recently by M. Haase.[26] This compound however did not perform similarly under Eschweiler-Clarke conditions. Table S2 shows the tested reaction parameters. Further reaction conditions were then applied.

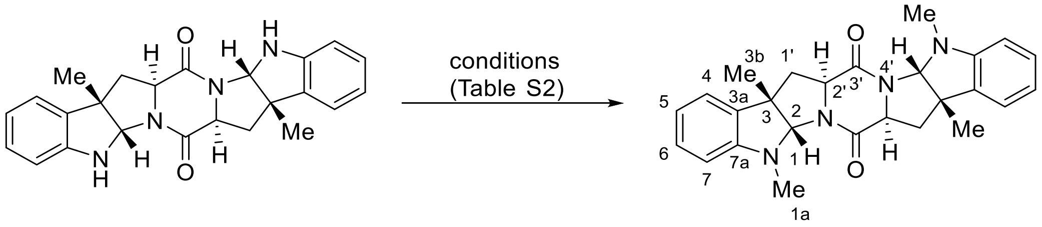

S13 **S14**

The substrate **S13** (5 mg, 12 µmol, 1.0 equiv.) was dissolved in solvent (1 mL). While stirring, the given reagents were added and stirring continued for the stated time at the stated temperature. In various entries, different methylation agents were tested. The reaction was monitored via TLC and MS.

Reactions with a stated qNMR, were worked up under aqueous conditions (addition of 1 mL of saturated sodium chloride solution) and extracted 4 times with 1 mL of ethyl acetate). The combined organic phases were dried over magnesium sulfate and the solvent removed. qNMR was performed with trimethoxy benzene (2-3 mg) as the internal standard and the amount of product was calculated with integral of the *N*-methyl peak.

Reactions with a stated isolated yield, were worked up under aqueous conditions (addition of 1 mL of saturated sodium chloride solution) and extracted 4 times with 1 mL of ethyl acetate). The combined organic phases were dried over magnesium sulfate and the solvent removed. Chromatographic purification was performed afterwards (2/1 petroleumether/ethyl acetate) to give the *N*-methylated product **S14**.

Table S3: Screened reaction conditions for the *N*-methylation of bis-hexahydropyrroloindole-DKPs

	Solv.	Reagent 1	Reagent 2	Time	Temp. [°C]	Outcome	Yield
**1**	DMSO	*p*-formaldehyde (3.0 equiv.)	formic acid (3.0 equiv.)	3 min	80¹	SM recovered	
**2**	DMSO	*p*-formaldehyde (3.0 equiv.)	formic acid (3.0 equiv.)	3 min	100¹	Substrate/Intermediate and product detected	
**3**	DMSO	*p*-formaldehyde (3.0 equiv.)	formic acid (3.0 equiv.)	3 min	120¹	decomposition	
**4**	DSMO 1:1 H_2O	*p*-formaldehyde (10 equiv.)	formic acid (10 equiv.)	1 min	80¹	SM recovered	
**5**	DSMO 1:1 H_2O	*p*-formaldehyde (10 equiv.)	formic acid (10 equiv.)	1 min	120¹	Decomp. + Product	**iso: 5%**
**6**	THF	MeI (10 equiv.)	KO*t*Bu (2.5 equiv.)	16 + 4 h	RT - later 40	Substrate/Intermediate and product detected	**iso: 11%**

**7**	DCM	MeI (10 equiv.)	K2CO3 (2.5 equiv.)	16 + 4 h	RT - later 40	SM recovered	
**8**	DMF	DiMeCO_3 (10 equiv.)	DABCO (0.1 equiv.)	2 + 16 h	90 - later RT	SM recovered	
**9**	DCM	Meerw. (4.0 equiv.)	Prot.sp. (5.0 equiv.)	3 d	RT	Substrate/Intermediate and product detected	**qNMR: 36%**
**10**	DCM	Meerw. (8.0 equiv.)	Prot.sp. (10.0 equiv.)	3 d	RT	only product	**qNMR: 77%**
**11**	DCM	Meerw. (16 equiv.)	Prot.sp. (20 equiv.)	3 d	RT	only product	**qNMR: 7%**
**12**	DCM	Meerw. (4.0 equiv.)	Prot.sp. (5.0 equiv.)	3 d	40	Substrate/Intermediate and product detected	**qNMR: 56%**
**13**[2]	DCM	Meerw. (8.0 equiv.)	Prot.sp. (10.0 equiv.)	1.5 h	RT		**72 %**

[1]: microwave assisted heating. [2]: Upscaled amount of substrate **S10** (18.2 mg, 46 µmol)

Entries 1-5 utilized Eschweiler-Clarke conditions (reductive amination) in very short reaction times using microwave-assisted heating. When performing the reaction at 80 °C, the desired reaction did not occur. The substrate was recovered and no product was detected via MS. Raising the temperature to 100 °C led to some product formation, but major amounts of substrate and intermediate did not react though. However, at 120 °C only product was detected via MS, but major amounts of the compound seemed to degrade. Shortening the reaction times to prevent degradation led to an isolated yield of 11% of the desired product, while no substrate or intermediate could be isolated.

Entries 6-8 tested for more convenient methylation agents, such as methyl iodide and dimethylcarbonate. Only in entry 6 (methyl iodide and potassium *tert*-butoxide) product was formed and a yield of 11% was isolated. The reaction was performed at RT for 16 hours, and for 4 hours at 40 °C (bp. of methyl iodide is 42 °C).

In Entries 9-13 Meerwein's salt and proton sponge was tested, showing the highest conversions towards the product. Given that no decomposition was observed over time, the reactions were carried out for a period of three days to ensure that the maximal amount of substrate was converted and to enable an estimation of the quantity of reagent required. Since entry 11 only showed 7% yield in qNMR, very high amounts of the reagents seemingly leads to degradation or sideproduct formation, possibly formation of an overmethylated product (quaternary carbon kation). Raising the temperature to 40 °C (entry 12) led to higher yields with respect to entry 8, where the same amounts of reagents were used. With a yield of 77% (measured via qNMR), 10 equivalents of proton sponge and 8.0 equivalents of Meerwein's salt showed the best results in this screening. Afterwards this condition was applied to a raised amount of substrate in entry 13, resulting in an isolated yield of 72% of *N*-methylated product **S14**.

Yield: 14 mg, 32 µmol, 72 %.

^{1}H-NMR (600 MHz, $CDCl_3$) δ 7.09 (t, $^3J_{6,5,7}$ = 7.6 Hz, 2H, 6-H), 7.04 (d, $^3J_{4,5}$ = 7.3 Hz, 2H, 4-H), 6.70 (t, $^3J_{5,4,6}$ = 7.4 Hz, 2H, 5-H), 6.34 (d, $^3J_{7,6}$ = 7.8 Hz, 2H, 7-H), 5.44 (s, 2H, 2-H), 4.16

(dd, $^3J_{2',1'}$ = 11.0 Hz, $^3J_{2',1'}$ = 6.0 Hz, 2H, 2'-H), 2.97 (s, 6H, 1a-H), 2.70 (dd, $^2J_{1',1'}$ = 12.8 Hz, $^3J_{1',2'}$ = 5.9 Hz, 2H, 1'-H_b), 2.17 (dd, $^2J_{1',1'}$ = 11.9 Hz, $^3J_{1',2'}$ = 11.9 Hz, 2H, 1'-H_a), 1.47 (s, 6H, 3b-H).

^{13}C-NMR (151 MHz, CD_3OD) δ 167.8 (C-3'), 151.6 (C-7a), 134.6 (C-3a), 129.7 (C-4), 123.3 (C-6), 119.3 (C-5), 107.1 (C-7), 88.1 (C-2), 61.2 (C-2'), 51.5 (C-3), 43.6 (C-1'), 33.3 (C-1a), 25.8 (C-3b).

HRMS (ESI): m/z calc. for $C_{26}H_{28}N_4O_2$+H^+: 429.2285 $[M+H]^+$; found: 429.2285.

Optical Rotation: $[\alpha]_D^{20}$ = -437 (c = 0.200, MeOH).

Melting Point: 148 °C.

2.6. *N*-Methylation towards 5-bromo-hexahydropyrroloindole S15

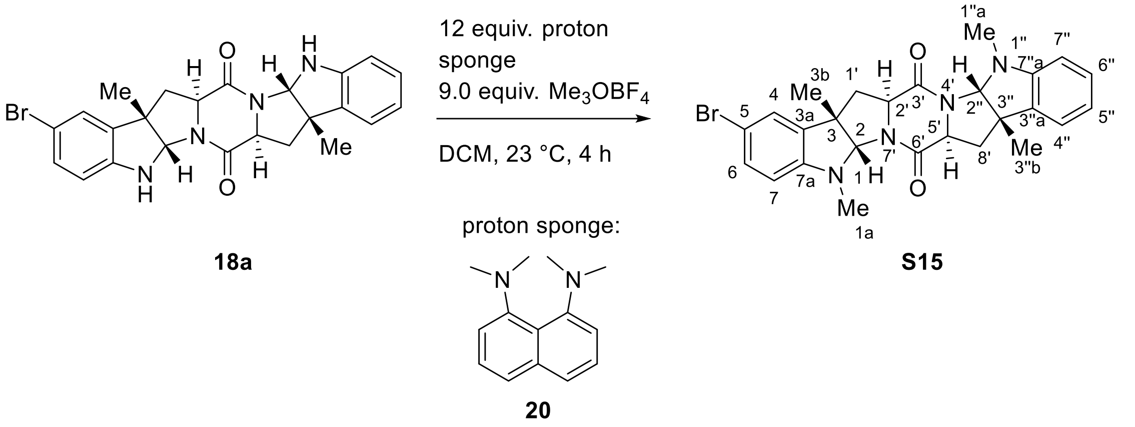

Pyrroloindole **13a** (20 mg, 41 µmol, 1.0 equiv.) was dissolved in DCM and stirred at 23 °C under argon. Proton sponge (36 mg, 0.17 mmol, 4.0 eq.) and Meerwein's salt (18 mg, 0.13 mmol, 3.0 equiv.) were added and the mixture stirred for 1.5 hours. Proton sponge and Meerwein's salt were added in small potions up to a total amount of 12 equiv. (proton sponge) and 9.0 equiv. (Meerwein's salt) while stirring for 4 hours. The reaction was then stopped by removal of the solvent under reduced pressure. After chromatographic purification (/1 petroleumether/ethyl acetate) the *N*-dimethylated product **S15** was obtained as white powder.

Yield: 7 mg, 14 µmol, 33%. (Starting material and one-site *N*-methylated product was recovered.)

^{1}H-NMR (600 MHz, CD_3OD) δ 7.21 (d, $^4J_{4,6}$ = 2.0 Hz, 1H, 4-H), 7.15 (dd, $^3J_{6,7}$ = 8.3 Hz, $^4J_{6,4}$ = 2.0 Hz, 1H, 6-H), 7.08 (dd, $^3J_{4“,5“}$ = 7.4 Hz, $^4J_{4“,6“}$ = 1.2 Hz, 1H, 4“-H), 7.05 (td, $^3J_{6“,5“,4“}$ = 7.7 Hz, $^4J_{6“,4“}$ = 1.2 Hz, 1H, 6“-H), 6.67 (td, $^3J_{5“,6“,4“}$ = 7.4 Hz, $^4J_{5“,7“}$ = 1.0 Hz, 1H, 5“-H), 6.38 (d, $^3J_{7“,6“}$ = 7.8 Hz, 1H, 7“-H), 6.29 (d, $^3J_{7,6}$ = 8.3 Hz, 1H, 7-H), 5.40 (s, 1H, 2-H), 5.38 (s, 1H, 2“-H), 4.27 – 4.09 (m, 2H, 2‘-H/5‘-H), 2.95 (s, 3H, 1“a-H), 2.92 (s, 3H, 1a-H), 2.59 (app. ddd, *J* = 13.5, 7.8, 6.1 Hz, 2H, 1‘-H/8‘-H), 2.20 (app. ddd, *J* = 12.5, 11.2, 4.9 Hz, 2H, 8‘-H/1‘-H), 1.43 (s, 3H, 3“b/3“a-H), 1.42 (s, 3H, 3“b/3“a-H).

^{13}C-NMR (151 MHz, CD_3OD) δ 167.8 (C-6‘/3‘), 167.6 (C-3‘/6‘), 151.6 (C-4“), 150.8 (C-4), 137.2 (C-3a), 134.6 (C-3“a), 132.4 (C-6), 129.8 (C-6”), 126.6 (C-4), 123.3 (C-4”), 119.3 (C-5”), 110.4 (C-5), 108.4 (C-7), 107.1 (C-7”), 88.1 (C-2/2’), 88.0 (C-2’/2), 61.2 (C-2’/5’), 61.1 (C-5’/2’), 51.6 (C-3/3”), 43.5 (C-1’/8’), 43.4 (C-1’/8’), 33.3 (C-1a/1”a), 33.2 (C-1a/1”a), 25.8 (C-3b/3“b), 25.7 (C-3b/3“b).

^{1}H-NMR (600 MHz, $CDCl_3$) δ 7.17 (dd, $^3J_{6,7}$ = 8.3, $^5J_{6,4}$ = 2.0 Hz, 1H, 6-H), 7.11 (d, $^4J_{4,6}$ = 2.1 Hz, 1H, 4-H), 7.09 (td, $^3J_{6“,5“,7“}$ = 7.7 Hz, $^4J_{6“,4“}$ = 1.3 Hz, 1H, 6“-H), 7.04 (dd, $^3J_{4“,5“}$ = 7.4 Hz, $^4J_{4“,6“}$ = 1.2 Hz, 1H, 4“-H), 6.70 (t, $^3J_{5“,6“,7“}$ = 7.4 Hz, 1H, 5“-H), 6.34 (d, $^3J_{7,6}$ = 7.8 Hz, 1H, 7-H), 6.20 (d, $^3J_{7“,6“}$ = 8.3 Hz, 1H, 7“-H), 5.43 (s, 2H, 2-H/2“-H), 4.16 (dd, $^3J_{2‘/5‘,1‘/8‘}$ = 11.1 Hz, 5.9 Hz, 2H, 2‘-H, 5‘-H), 2.97 (s, 3H, 1“a-H), 2.94 (s, 3H, 1a-H), 2.70 (dd, $^2J_{1',1'}$ =12.9 Hz, $^3J_{1',2'}$ = 6.2 Hz, 1H, 1‘-H_a), 2.67 (dd, $^2J_{8',8'}$ = 13.0 Hz, $^3J_{8',8'}$ = 6.2 Hz, 1H, 8’-H_a), 2.17 (dd, $^2J_{8',8'}$ = 13.7 Hz, $^3J_{8',5'}$ = 2.4 Hz, 1H, 8’-H_b), 2.16 (dd, $^2J_{8',8'}$ = 13.6 Hz, $^3J_{8',5'}$ = 2.4 Hz, 1H, 8’-H_b), 1.47 (s, 3H, 3b-H/3“b-H), 1.46 (s, 3H, 3b-H/3“b-H).

^{13}C NMR (151 MHz, $CDCl_3$) δ 165.7 (C-6‘/3‘), 165.4 (C-3‘/6’), 150.3 (C-4“), 149.3 (C-4), 135.4 (C-3“a), 133.0 (C-3a), 131.5 (C-6), 128.9 (C-6”), 125.7 (C-4), 122.5 (C-4”), 118.2 (C-5”), 109.6 (C-5), 107.3 (C-7”), 106.0 (C-7), 86.7 (C-2/2’), 86.6 (C-2/2’), 60.3 (C-2’/5’), 60.2 (C-2’/5’), 50.4 (C-3/3”), 50.4 (C-3/3”), 42.8 (C-1’/8’), 42.7 (C-1’/8’), 33.1 (C-1a/1”a), 33.0 (C-1a/1”a), 25.5 (C-3b/3“b), 25.4 (C-3b/3“b).

HRMS (ESI): m/z calc. for $C_{26}H_{28}N_4O_2Br+H^+$: 507.1390 $[M+H]^+$; found: 507.1393.

IR (ATR): ṽ [cm^{-1}] = 2951 (w), 2924 (w), 2864 (w), 1741 (m), 1667 (vs), 1606 (m), 1492 (s), 1418 (s), 1294 (m), 1210 (m), 1163 (m), 1082 (w), 1001 (w), 806 (w), 746 (w).

Optical Rotation: $[\alpha]_D^{20}$ = -396 (c = 0.643, $CHCl_3$)

2.7. Suzuki coupling towards 5-prenyl-hexahydropyrroloindol 19

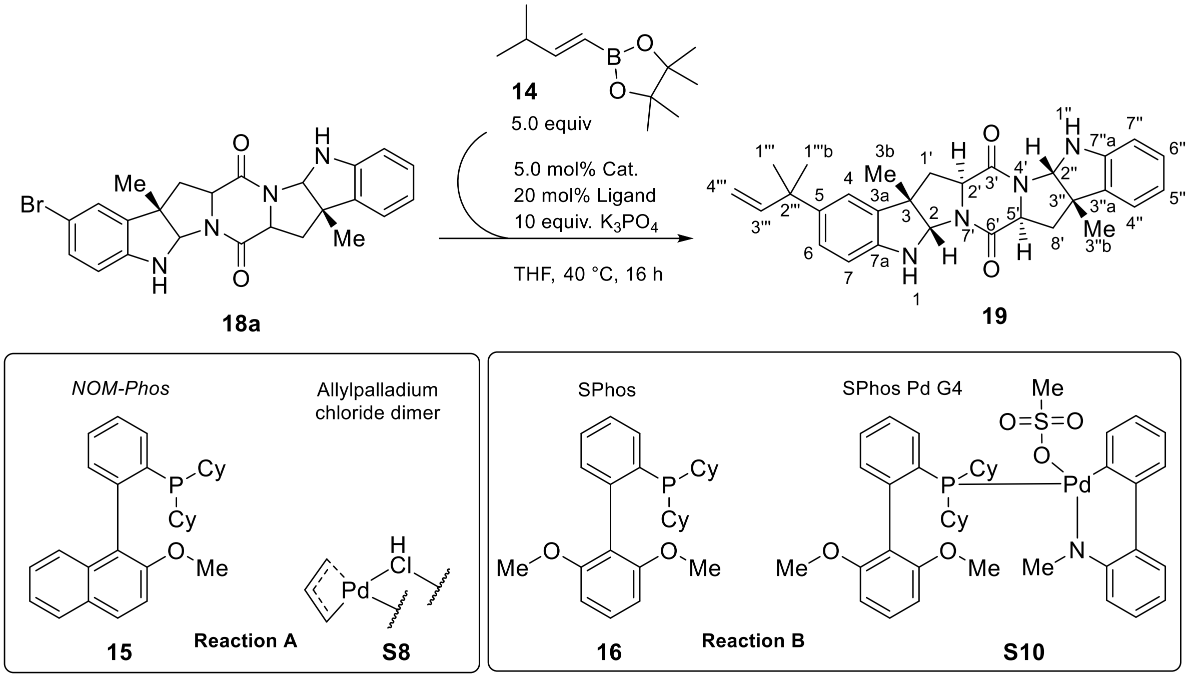

Reaction A: Allylpalladium chloride (1.1 mg, 3.0 µmol, 5.0 mol%), *NOM-Phos* (5.2 mg, 12 µmol, 20 mol%) and potassium carbonate (128 mg, 0.60 mmol, 10 equiv.) were given to a Schlenk flask under argon. Prenylpinacolborane (59 mg, 0.30 mmol, 5.0 equiv.) and DKP **19c** (29 mg, 60 µmol, 1.0 equiv.) were both dissolved in oxygen-free-THF (1 mL each, freeze-pump-thawed) and added to the reaction flask. Oxygen-free water (1 mL, freeze-pump-thawed) was added and the mixture stirred at 40 °C for 16 hours. Then, the mixture was cooled down, diluted with ethyl acetate (10 mL) and neutralized by addition of an aqueous, saturated sodium chloride solution (5 mL). The organic phase was separated and the aqueous phase was extracted with ethyl acetate (3x5 mL). Afterwards, the organic phases were combined, washed with an aqueous, saturated solution of sodium chloride, dried over magnesium sulfate and the solvent removed under reduced pressure. After chromatographic purification (1/1 petroleumether/ethyl acetate) 5-prenyl-cWW-DKP was obtained as a white powder.

Reaction B: SPhos Pd G4 (1.3 mg, 1.6 µmol, 5.0 mol%), SPhos (2.7 mg, 6.6 µmol, 20 mol%) and potassium carbonate (70.0 mg, 0.33 mmol, 10 equiv.) were given to a Schlenk flask under argon. Prenylpinacolborane (32.4 mg, 0.17 mmol, 3.1 equiv.) and DKP **19c** (15.9 mg, 33 µmol, 1.0 equiv.) were both dissolved in oxygen-free-THF (1 mL each, freeze-pump-thawed) and added to the reaction flask. Oxygen-free water (1 mL, freeze-pump-thawed) was added and the mixture stirred at 40 °C for 16 hours. Then, the mixture was cooled down, diluted with ethyl acetate (10 mL) and neutralized by addition of an aqueous, saturated sodium chloride solution (5 mL). The organic phase was separated and the aqueous phase was extracted with ethyl acetate (3x5 mL). Afterwards, the organic phases were combined, washed with an aqueous, saturated solution of sodium chloride, dried over magnesium sulfate and the solvent removed under reduced pressure. After chromatographic purification (1/1 petroleumether/ethyl acetate) 5-prenyl-cWW-DKP was obtained as a white powder.

Yield Reaction A: 22.3 mg, 47 µmol, 78%. **Yield Reaction B:** 8.0 mg, 17 µmol, 52%.

^{1}H-NMR (600 MHz, CD_3OD) δ 7.14 (d, $^{4}J_{4,6}$ = 2.0 Hz, 1H, 4-H), 7.06 (d, $^{3}J_{4'',5''}$ = 7.3 Hz, 1H, 4“-H), 7.01 (dd, $^{3}J_{6,7}$ =8.2Hz, $^{4}J_{6,4}$ = 2.0 Hz, 1H, 6-H), 6.98 (td, $^{3}J_{6'',5'',7''}$ =7.6 Hz, $^{4}J_{6'',4''}$ = 1.2 Hz, 1H, 6“-H), 6.69 (td, $^{3}J_{5'',6'',4''}$ =7.4 Hz, $^{4}J_{5'',7''}$ = 1.1 Hz, 1H, 5“-H), 6.53 (d, $^{3}J_{7'',6''}$ = 7.8 Hz, 1H, 7“-H),

6.51 (d, $^{3}J_{7,6}$ = 8.1 Hz, 1H, 7-H), 5.98 (dd, $^{3}J_{3''',4'''}$ = 17.4, 10.6 Hz, 1H, 3‘“-H), 5.21 (s, 1H, 2-H/2“-H), 5.20 (s, 1H, 2“-H/2-H), 5.00 (dd, $^{3}J_{4''',3'''}$ = 17.5 Hz, J = 1.5 Hz, 1H, 4‘“-H_a), 4.97 (dd, $^{3}J_{4''',3'''}$ = 10.6 Hz, J = 1.5 Hz, 1H, 4‘“-H_b), 4.06 – 3.93 (m, 2H, 2‘-H/5‘-H), 2.63 (app. dt, J = 12.6, 6.3 Hz, 2H, 1‘-H/8‘-H), 2.22 (app. ddd, J = 12.6, 11.3, 3.1 Hz, 2H, 1‘-H/8‘-H), 1.41 (s, 3H, 3b-H), 1.40 (s, 3H, 3“b-H), 1.34 (s, 6H, 1‘“-H, 1‘“b-H).

^{13}C-NMR (151 MHz, CD_3OD) δ 168.2 (C-3“/6“), 168.1 (C-6“/3“), 150.3 (C-7“a), 150.0 (C-3’”), 148.3 (C-7a), 141.1 (C-5), 133.9 (C-3a/3”a), 133.9 (C-3”a/3a), 129.5 (C-6”), 127.5 (C-6), 123.7 (C-4”), 121.4 C-4), 120.3 (C-5”), 110.6 (C-7”/4’”), 110.5 (C-4’”/7”), 110.3 (C-7), 82.8 (C-2/2”), 82.3 (C-2”/2), 61.4 (C-2’/5’), 61.4 (C-5’/2’), 53.2 (C-3/3”), 53.0 (C-3”/3), 41.8 (C-1’/8’/2’”), 41.7 (C-1’/8’/2’”), 41.6 (C-1’/8’/2’”), 29.1 (C-1’”/1’”b), 25.1 (C-3b/3”b), 25.0 (C-3”b/3b).

^{1}H-NMR (600 MHz, $CDCl_3$) δ 7.06 (d, $^{3}J_{4'',5''}$ = 7.4 Hz, 1H, 4”-H), 7.04 – 6.98 (m, 3H, 4-H, 6-H, 6”-H), 6.74 (t, $^{3}J_{5'',4'',6''}$ = 7.4 Hz, 1H, 5”-H), 6.51 (d, $^{3}J_{7'',6''}$ = 7.8 Hz, 1H, 7”-H), 6.46 (d, $^{3}J_{7,6}$ = 8.0 Hz, 1H, 7-H), 5.93 (dd, $^{3}J_{3''',4'''}$ = 17.4, 10.6 Hz, 1H, 3’”-H), 5.19 (s, 1H, 2-H/2”-H), 5.18 (s, 1H, 2”-H/2-H), 5.01 (s, 1H, N-H), 4.01 – 4.96 (m, 2H, 4’”-H), 4.94 (s, 1H, N-H), 4.02 – 3.94 (m, 2H, 2’-H/5’-H), 2.70 (app. ddd, J = 12.7, 8.8, 6.0 Hz, 2H, 1’-H/8’-H), 2.26 (app. dt, J = 12.7, 10.6 Hz, 2H, 1’-H/8’-H), 1.44 (s, 3H, 3b-H/3”b-H), 1.43 (s, 3H, 3”b-H/3b-H), 1.31 (s, 6H, 1’”/1’”b-H).

^{13}C NMR (151 MHz, $CDCl_3$) δ 167.0 (C-3’/6’), 166.7 (C-6’/3’), 148.6 (C-3’”),148.4 (C-7a/7”a), 146.3 (C-7”a/7a), 140.4 (C-5), 132.2 (C-3a/3”a), 132.1 (C-3”a/3a), 128.8 (C-6), 126.5 (C-6”), 122.7 (C-4”), 120.4 (C-4), 119.8 (C-5”), 110.4 (C-4’”), 109.6 (C-7”), 109.2 (C-7), 81.7 (C-2/2”), 81.3 (C-2”/2), 60.7 (C-2’/5’), 60.7 (C-5’/2’), 52.2 (C-3/3”), 52.1 (C-3”/3), 40.9 (C-2’”), 39.9 (C-1’/8’), 39.9 (C-8’/1’), 28.7 (C-1’”/1’”b), 28.6 (1’”b/1’”), 24.2 (C-3b/3”b), 24.1 (C-3”b/3b).

Notably, the CD_3Cl spectra were measured after the CD_3OD-spectra containing some additional peaks in high field. During the process of adding and removing the solvent under reduced pressure, the compound seems to degrade to some extent, which explains the additional peaks.

HRMS (ESI): m/z calc. for $C_{29}H_{32}N_4O_2$+H^+: 469.2598 $[M+H]^+$; found: 469.2600.

IR (ATR): ṽ [cm^{-1}] = 3356 (br), 2961 (w), 2929 (w), 2869 (w), 1659 (vs), 1611 (m), 1485 (m), 1420 (s), 1290 (m), 1191 (m), 1164 (m), 908 (s), 729 (vs).

Optical Rotation: $[\alpha]_D^{20}$ = -578 (c = 0.166, $CHCl_3$)

2.8. *N*-Methylation of 5-prenyl-hexahydropyrroloindole-DKP to (-)-Iansai B (3)

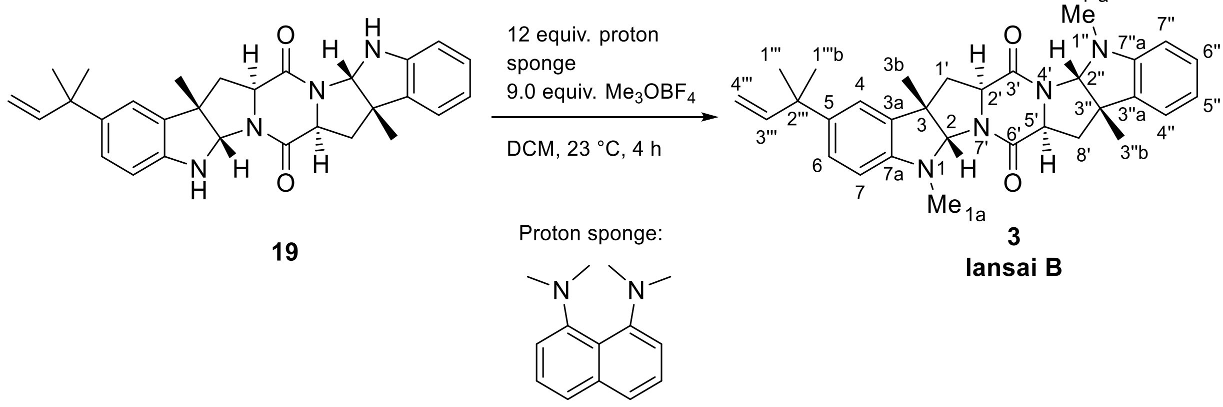

19

3
Iansai B

21

Pyrroloindole **20** (4 mg, 8.5 µmol, 1.0 equiv.) was dissolved in DCM (2 mL) and stirred at 23 °C under argon. Proton sponge (7.2 mg, 34 µmol, 4.0 eq.) and Meerwein's salt (3.7 mg, 26 µmol, 3.0 equiv.) were added and the mixture stirred for 3 hours. Proton sponge and Meerwein's salt were added in small potions up to a total amount of 8.0 equiv. (proton sponge) and 7.0 equiv. (Meerwein's salt) while stirring for 5 hours, when no starting material and intermediate was detected. The reaction was then stopped by removal of the solvent under reduced pressure. The crude product was chromatographically purified (2/1 petroleumether/ethyl acetate) to give Iansai B (**3**) as a white powder.

Yield: 3.4 mg, 6.7 µmol, 80%.

^{1}H-NMR (600 MHz, CDCl$_3$) δ 7.12 – 7.08 (m, 1H, 4”-H), 7.07 (dd, $^{3}J_{6,7}$ = 8.1, $^{4}J_{6,4}$ = 1.9 Hz, 1H, 6-H), 7.04 (dd, $^{3}J_{6'',5''}$ = 7.4 Hz, $^{4}J_{6'',4''}$ = 1.3 Hz, 1H, 6”-H), 7.01 (d, $^{4}J_{4,6}$ = 1.9 Hz, 1H, 4-H), 6.72 (t, $^{3}J_{5'',4'',6''}$ = 7.4 Hz, 1H, 3”-H), 6.34 (d, $^{3}J_{7'',6''}$ = 7.9 Hz, 1H, 7”-H), 6.27 (d, $^{3}J_{7,6}$ = 8.1 Hz, 1H, 7-H), 5.96 (dd, $^{3}J_{3''',4'''}$ = 17.4, $^{3}J_{3''',4'''}$ = 10.6 Hz, 1H, 3’”-H), 5.44 (s, 1H, 2”-H), 5.42 (s, 1H, 2-H), 5.01 (dd, $^{3}J_{4''',3'''}$ = 17.0 Hz, $^{2}J_{4''',4'''}$ = 1.4 Hz, 1H, 4’”-H$_a$), 4.98 (d, $^{3}J_{4''',3'''}$ = 10.3 Hz, $^{2}J_{4''',4'''}$ = 1.4 Hz, 1H, 4’”-H$_b$), 4.19 – 4.11 (m, 2H, 2’-H, 5’-H), 2.97 (s, 3H, 1”a-H), 2.95 (s, 3H, 1a-H), 2.70 (app. ddd, J = 12.7, 6.0, 4.2 Hz, 2H, 1’a-H, 8’a-H), 2.24 – 2.13 (m, 2H, 1’a-H, 8’b-H), 1.47 (s, 3H, 3b-H), 1.46 (s, 3H, 3”b-H), 1.34 (s, 6H, 1’”-H, 1’”b-H).

^{13}C NMR (151 MHz, CDCl$_3$) δ 165.9 (C-3’), 165.7 (C-6’), 150.3 (C-7”a), 148.7 (C-3’”), 148.4 (C-7a), 138.8 (C-5), 133.0 (C-3a), 132.9 (C-3”a), 128.9 (C-4”), 126.4 (C-6), 122.5 (C-6”), 120.4 (C-4), 118.2 (C-5”), 110.3 (C-4’”), 106.0 (C-7”), 105.5 (C-7), 87.0 (C-2”), 86.7 (C-2), 60.3 (C-5’), 60.3 (C-2’), 50.6 (C3), 50.4 (C-3”), 42.9 (C-8’), 42.8 (C-1’), 40.8 (C-2’”), 33.3 (C-1a), 33.1 (C-1”), 28.7 (C-1’”), 28.7 (C-1’”b), 25.6 (C-3”b), 25.6 (C-3b).

^{1}H-NMR (700 MHz, CDCl$_3$) δ 7.12 – 7.10 (m, 1H, 4”-H), 7.09 (dd, $^{3}J_{6,7}$ = 8.1, $^{4}J_{6,4}$ = 1.6 Hz, 1H, 6-H), 7.04 (dd, $^{3}J_{6'',5''}$ = 7.4 Hz, $^{4}J_{6'',4''}$ = 1.3 Hz, 1H, 6”-H), 7.03 (d, $^{4}J_{4,6}$ = 1.9 Hz, 1H, 4-H), 6.72 (td, $^{3}J_{5'',4'',6''}$ = 7.4, $^{4}J_{4'',6''}$ = 0.9 Hz, 1H, 5”-H), 6.37 (d, $^{3}J_{7,6}$ = 7.9 Hz, 2H, 7-H), 6.37 (d, $^{3}J_{7'',6''}$ = 7.9 Hz, 2H, 7”-H), 5.96 (dd, $^{3}J_{3''',4'''}$ = 17.4, $^{3}J_{3''',4'''}$ = 10.6 Hz, 1H, 3’”-H), 5.44 (s, 1H, 2”-H), 5.43 (s, 1H, 2-H), 5.02 (d, $^{3}J_{4''',3'''}$ = 17.3 Hz, $^{2}J_{4''',4'''}$ = 1.4 Hz, 1H, 4’”-H$_a$), 5.00 (dd, $^{3}J_{4''',3'''}$ = 10.5 Hz, $^{2}J_{4''',4'''}$ = 1.5 Hz, 1H, 4’”-H$_b$), 4.22 (ddd, $^{3}J_{2',1'}$ = 11.1 Hz, $^{3}J_{2',1'}$ = 6.2, $^{4}J_{2',2}$ = 1.9 Hz, 1H, 2’-H), 4.18 (ddd, $^{3}J_{5',8'}$ = 11.1 Hz, $^{3}J_{5',8'}$ = 6.1 Hz, $^{4}J_{5',2''}$ = 2.0 Hz, 1H, 5’-H), 2.98 (s, 3H, 1”a-H), 2.97 (s, 3H, 1a-H), 2.70 (app. ddd, J = 12.8, 6.1, 3.3 Hz, 2H, 1’a-H, 8’a-H), 2.19 (app. ddd, J = 12.9, 11.1, 2.2 Hz, 2H, 1’b-H, 8’b-H), 1.48 (s, 3H, 3b-H), 1.47 (s, 3H, 3”b-H), 1.35 (s, 6H, 1’”-H, 1’”b-H).

^{13}C NMR (176 MHz, $CDCl_3$) δ 165.9 (C-3’), 165.9 (C-6’), 150.0 (C-7”a), 148.5 (C-3’”), 147.4 (C-7a), 140.0 (C-5), 133.4 (C-3a), 133.1 (C-3”a), 128.9 (C-4”), 126.6 (C-6), 122.5 (C-6”), 120.5 (C-4), 118.5 (C-5”), 110.5 (C-4’”), 106.7 (C-7”), 106.3 (C-7), 87.3 (C-2”), 86.8 (C-2), 60.3 (C-5’), 60.3 (C-2’), 50.7 (C-3), 50.5 (C-3”), 42.8 (C-8’), 42.8 (C-1’), 40.9 (C-2’”), 33.3 (C-1a/C-1”a), 29.9 (C-1’”), 28.6 (C-1’”a), 25.7 (C-3”b), 25.6 (C-3b).

Notably, the 700 MHz-spectra were measured some days after the 600 MHz-spectra containing some additional peaks in high field. Dissolved in $CDCl_3$ within this time, the compound seems to degrade to some extent, which explain the additional peaks.

HRMS (ESI): m/z calc. for $C_{31}H_{36}N_4O_2+H^+$: 497.2911 $[M+H]^+$; found: 497.2916.

IR (ATR): ṽ [cm^{-1}] = 2967 (m), 2924 (s), 2848 (m), 1735 (m), 1668 (vs), 1602 (m), 1494 (s), 1416 (s), 1299 (s), 1207 (s), 1163 (s), 1083 (m), 1004 (m), 909 (m), 814 (w), 733 (s).

Optical Rotation: $[\alpha]_D^{20}$ = -531 (c = 0.268, $CHCl_3$), Lit.: $[\alpha]_D^{25}$ = -505 (c = 0.12, $CHCl_3$).[14]

The ^{1}H- and ^{13}C-NMR spectra are consistent with the data presented in the literature.[7,14]

3. <u>Biological Methods</u>

3.1. Protein sequence

The gene sequence encoding the methyltransferase SgMT (GenBank: GGT26788.1) and the tryptophan synthase β-subunit Pf0A9 (mutant yielded via directed evolution of the native parental enzyme TrpB, GenBank: AB080770.1) were optimized for codon usage and synthetically synthesized within a pET28a (SgMT) or pET21a ($TrpB^{Pf0A9}$) expression vector by GenScript (USA).

C-terminal His-tagged SgMT:

Nucleotide sequence:

ATGGGCCATATGTCGAGCCAGACCGTTACCCCGGATCCGTATGGTAACCTGGCAGAAAG
CTATGATCGTCTGGCACAGTGGGCAATTGATCAGCAGCAGGAGAGCCCCCGTGATCGTG
TTGGGGACTTTCTGCAGACCTTTTGGCAGAGCCAGGATCGTCCGGTTAGGACCGTTCTG
GAAATTTGTTGTGGTACCGGTCTGATGCTGGCAGAACTGGCTCGTCGTGGGTATGTTGT
TACCGGGCTGGATCGTTCGGCAGCAATGCTGGAGCAGGCTCGTGCACGTATGGGTGGA
AAAACCACTCTGATTAGGGCAGAGCTGCCGGATATTCCGGCACCGGCAGGGGAATTTGA
TGCAGTTGTTAGCGCAGCAGGGGGACTGAATTATCTGAGCGAATCGCAGATTAGCGCAA
CCTTTGGTGCAGTTGCAAGGCTGCTGCCGGCAGGAGGTACCTTTACCTTTGATGTTTTT
GGTCAGGGGTTTTATGCAAAATTTTTTGATCCGTCGGCACCGCGTGTTATGGCACTGGAG
CTGGATGATATTAGCTATATTTGGACCTTTACCAAACCGGCAGAAGCACCGTTTGTTGATA
TGAGCTATACCCAGTTTAGCCCGGCATCGCGTGCAGTTGATGGGGAACCCGCATTTATTC
GTACCAGGGATCTGCACCGTTATTATCCGCTGCCGCATGCAACCGTTCTGCGTCTGGCA
GCAGAACATGGGTTTACCGATGCACGTGCACATGATAATTATAGCAGCGATCCGAGCGGT
CCGCATACCCTGTATGATACCTGGACTATGGTTCGTACCGGATCGCTCGAGCACCACCAC
CACCACCACTGA

Protein sequence:

MGHMSSQTVTPDPYGNLAESYDRLAQWAIDQQQESPRDRVGDFLQTFWQSQDRPVRTVL
EICCGTGLMLAELARRGYVVTGLDRSAAMLEQARARMGGKTTLIRAELPDIPAPAGEFDAVV
SAAGGLNYLSESQISATFGAVARLLPAGGTFTFDVFGQGFYAKFFDPSAPRVMALELDDISYI
WTFTKPAEAPFVDMSYTQFSPASRAVDGEPAFIRTRDLHRYYPLPHATVLRLAAEHGFTDAR
AHDNYSSDPSGPHTLYDTWTMVRTGSLEHHHHHH*

C-terminal His-tagged TrpB^{Pf0A9}

(Code adapted from Dick *et al.*[31], marked in red is the removed mutation E105G[30])

Nucleotide sequence:

ATGTGGTTCGGTGAATTTGGTGGTCAGTACGTGCCAGAAACGCTGGTTGGACCCCTGAA
AGAGCTGGAAAAAGCTTACAAACGTTTCAAAGATGACGAAGAATTCAATCGTCAGCTGAA
TTACTACCTGAAAACCTGGGCAGGTCGTCCAACCCCACTGTACTACGCAAAACGCCTGA
CTGAAAAAATCGGTGGTGCTAAAGTCTACCTGAAACGTGAAGACCTGGTTCACGGTGGT
GCACACAAGACCAACAACGCCATCGGTCAGGCACTGCTGGCAAAGCTCATGGGTAAAA
CTCGTCTGATCGCTGAGACCGGTGCTGGTCAGCACGGCGTAGCGACTGCAATGGCTGG
TGCACTGCTGGGCATGAAAGTGGACATTTACATGGGTGCTGAGGACGTAGAACGTCAGA
AACTGAACGTATTCCGTATGAAGCTGCTGGGTGCAAACGTAATTCCAGTTAACTCCGGTT
CTCGCACCCTGAAAGACGCATTTGACGAGGCTCTGCGTGATTGGGTGGCTACTTTTGAA
TACACCCACTACCTAATCGGTTCCGTGGTCGGTCCACATCCGTATCCGACCATCGTTCGT
GATTTTCAGTCTGTTATCGGTCGTGAGGCTAAAGCGCAGATCCTGGAGGCTGAGGGTCA
GCTGCCAGATGTAATCGTTGCTTGTGTTGGTGGTGGCTCTAACGCGATGGGTATCTTTTA
CCCGTTCGTGAACGACAAAAAAGTTAAGCTGGTTGGCGTTGAGGCTGGTGGTAAAGGC
CTGGAATCTGGTAAGCATTCCGCTAGCCTGAACGCAGGTCAGGTTGGTGTGTCCCATGG
CATGCTGTCCTACTTTCTGCAGGACGAAGAAGGTCAGATCAAACCAAGCCACTCCATCG
CACCAGGTCTGGATCATCCAGGTGTTGGTCCAGAACACGCTTACCTGAAAAAAATTCAG
CGTGCTGAATACGTGGCTGTAACCGATGAAGAAGCACTGAAAGCGTTCCATGAACTGAG
CCGTACCGAAGGTATCATCCCAGCTCTGGAATCTGCGCATGCTGTGGCTTACGCTATGAA
ACTGGCTAAGGAAATGTCTCGTGATGAGATCATCATCGTAAACCTGTCTGGTCGTGGTGA
CAAAGACCTGGATATTGTCCTGAAAGCGTCTGGCAACGTGCTCGAGCACCACCACCACC
ACCACTGAG

Protein sequence:

MWFGEFGGQYVPETLVGPLKELEKAYKRFKDDEEFNRQLNYYLKTWAGRPTPLYYAKRLTE
KIGGAKVYLKREDLVHGGAHKTNNAIGQALLAKLMGKTRLIAETGAGQHGVATAMAGALLGM
KVDIYMGAEDVERQKLNVFRMKLLGANVIPVNSGSRTLKDAFDEALRDWVATFEYTHYLIGS
VVGPHPYPTIVRDFQSVIGREAKAQILEAEGQLPDVIVACVGGGSNAMGIFYPFVNDKKVKLV
GVEAGGKGLESGKHSASLNAGQVGVSHGMLYFLQDEEGQIKPSHSIAPGLDHPGVGPEHA
YLKKIQRAEYVAVTDEEALKAFHELSRTEGIIPALESAHAVAYAMKLAKEMSRDEIIIVNLSGRG
DKDLDIVLKASGNVLEHHHHHH*

3.2. Bacterial strains and media

The cultivation and protein expression were carried out in *E. coli* BL21(DE3). For the preculture, LB liquid medium (lysogeny broth) containing 10 g/L tryptone, 5 g/L yeast extract, and 2 g/L sodium chloride was used. The main culture was grown in TB liquid medium (terrific broth; Carl Roth, Karlsruhe, Germany), which included 12 g/L casein, 24 g/L yeast extract, 12.54 g/L K_2HPO_4, 2.3 g/L KH_2PO_4, and 4 mL/L glycerol, supplemented with kanamycin (SgMT) or ampicillin (TrpB^{Pf0A9}) at a final concentration of 100 µg/mL. All culture media were prepared using distilled water and sterilized by autoclaving prior to use.

3.3. Proteinexpression

Competent *E. coli* BL21(DE3) cells were transformed with the desired plasmid by heat shock: 100 ng of plasmid DNA was added to 100 µL of competent cells and incubated on ice for 30 minutes. The cells were then heat-shocked in a 42 °C water bath for 90 seconds, followed by the addition of 700 µL of LB medium. The mixture was shaken at 37 °C for 1 hour. Afterward, the cells were centrifuged at 2000 rpm for 2 minutes, and the resulting pellet was resuspended in 100 µL of LB medium before plating on LB agar plates with kanamycin (SgMT) or ampicillin ($TrpB^{Pf0A9}$). The plates were incubated overnight at 37 °C. To prepare a preculture, a single colony from the transformed cells was inoculated into 5 mL of LB medium containing kanamycin (SgMT) or ampicillin ($TrpB^{Pf0A9}$) and incubated at 37 °C for 16 hours. This preculture was then used to inoculate the main culture (500 mL of TB medium) at a 1:100 dilution. The main culture was incubated at 37 °C with shaking at 130 rpm until the optical density at 600 nm (OD600) reached 0.5. At this point, protein expression was induced by adding IPTG to a final concentration of 100 µM. The culture was then incubated at 25 °C for 20 hours. Cells were harvested by centrifugation at 7000 g for 35 minutes at 4 °C, and the resulting cell pellet was stored at -20 °C for future use.

3.4. Mutagenesis

Amino acids at selected positions were modified using iterative saturation mutagenesis. Mutant libraries were created by applying the 22c-trick, which minimizes codon redundancy and reduces library size.[11,12] Degenerate primer mixtures were utilized in a whole-plasmid amplification PCR approach, with the intended mutation introduced at the center of the forward primer. The PCR mixture was prepared as outlined in Table 1, and the protocol in Table 2 was followed. After amplification, PCR products were digested with DpnI at 37 °C for 1 hour and analyzed on a 1% agarose gel (45 minutes at 100V) using Midori Green staining. The gel-purified products were ligated with T4 DNA ligase, using 6% (v/v) PEG 4000 to enhance efficiency. For transformation, 20 µL of the ligated product was added to chemically competent *E. coli* DH5α cells, which were heat-shocked to facilitate plasmid uptake. Plasmid isolation was performed with an Innuprep plasmid isolation kit (Analytik Jena), and sequencing confirmed successful introduction of the desired mutations.

**Component**	**Volume (µL)**
5X PrimeSTAr GXL Buffer	10
dNTP (2.5 mM)	4
Primer fw (10 µM)	1.5
Primer rev (10 µM)	1.5
Template vector (10 ng/µL)	0.7
PrimeSTAR polymerase	1
ddH2O	31.3
TOTAL	50

Step	Temperature (°C)	Time	Cycles
1	98	30 s	
2	98	10 s	17X
3	Touchdown	15 s	
4	68	3 min	
5	98	10 s	25X
6	55	15 s	
7	68	3 min	
8	68	5 min	
9	4	storage	

3.5. Enzyme purification of SgMT

A 200 mg cell pellet was resuspended in 1 mL of KP_i buffer (50 mM, pH 8) and lysed using glass bead (0.2 mm) cell disruption with a swing mill (Retsch, Type MM400) at 30 Hz for 10 minutes. Following lysis, the mixture was centrifuged at 7000 rpm for 30 minutes. The resulting lysate was combined with 100 µL of pre-washed Ni-NTA agarose beads and incubated on ice for 40 minutes with gentle inversion. To remove impurities, the beads were washed with 1 mL of KP_i buffer (50 mM, containing 40 mM imidazole, pH 7.5). The bound sgMT protein was then eluted using 500 µL of elution buffer (50 mM KP_i, 250 mM imidazole, pH 7.5). For buffer exchange and protein concentration, centrifugal concentrators (Vivaspin 500, 10,000 MWCO PES, Sartorius Stedim Biotech) were used. The concentrated protein solution, now in KP_i buffer (50 mM, pH 8), was snap-frozen in liquid nitrogen and stored at −20 °C for later use.

3.6. General procedure for Mtase glo assay

The Mtase Glo assay (Promega) was employed to determine methyltransferase activity following the manufacturer's protocol. Luminescence measurements were recorded using an Infinite plate reader (Tecan). Each reaction was performed in triplicate within a 96-well plate (Nunc™; flat-bottom, white polystyrene). Reaction mixtures included 3 µM enzyme, 25 µM SAM and 25 µM of the additional substrate. The reaction was incubated at 45 °C for 15 minutes and stopped by adding 0.5% TFA (finals concentration).

3.7. Enzymatic preparative scale methylation reaction with immobilized SgMT

The cell pellet was resuspended in reaction buffer (50 mM KP_i, pH 8) and lysed using an ultrasonic cell disruptor (Branson Sonifier II "Model W-250", Heinemann) for two 10-minute sessions at 35–40% amplitude. Following lysis, the cell debris was removed by centrifugation (4 °C, 10,000 × rcf, 20 minutes), and the supernatant was collected as the lysate. The lysate was incubated washed Protino® Ni-NTA agarose suspension at 4 °C for 40 minutes with gentle end-over-end mixing. After removing the supernatant, the enzyme-bound beads were washed

with washing solution (50 mM KP_i, pH 8, 40 mM imidazole) for 10 minutes at 4 °C. Residual imidazole was removed by two additional washes with reaction buffer (50 mM KP_i, pH 8).

The Ni-NTA-bound enzyme was then transferred to a glass bottle and the desired volume was adjusted with buffer. The reaction mixture was prepared by adding cWW substrate (dissolved in DMSO, 1 mM cWW final concentration) and S-adenosylmethionine disulfate tosylate (3 mM final concentration) in KP_i buffer (50 mM, pH 8). The reaction proceeded at 40 °C with gentle shaking at 300 rpm. After 48 hours, the Ni-NTA resin was removed by filtration. The product was extracted three times with ethyl acetate, dried over $MgSO_4$, and concentrated under reduced pressure. Finally, the product was purified via column chromatography using ethyl acetate as the eluent.

3.8. Reaction optimization (MTase reaction)

A reaction mixture was prepared with 1 mM 5-Br cWW, 2.5 mM SAM, and 10–100% lysate (v/v) od SgMT in KP_i buffer (50 mM, pH 7.5) in a total volume of 500 µL. The mixture was incubated at 45 °C with shaking at 700 rpm for 20 hours. To stop the reaction, TFA was added to a final concentration of 0.5% (v/v). A 50 µL aliquot of each sample was subsequently analyzed following the different assay procedures.

3.8.1. HPI Assay

A 50 µL aliquot of the analyte aqueous solution was pipetted into a 96-well clear microtiter plate. To this, 30 µL of 1 M H_2SO_4 was added, mixed thoroughly, and incubated for 10 minutes. Then, 2.5 µL of a 50 mM cerium sulfate solution (in water with 0.5% v/v H_2SO_4) was added, and the mixture was well-mixed. Absorbance was measured at 470 nm using a Tecan® plate reader.

3.8.2. Indole Assay

A 50 µL aliquot of the analyte aqueous solution was pipetted into a clear 96-well microtiter plate. To this, 50 µL of concentrated H_2SO_4 (98%) was added, and the mixture was incubated at room temperature for 10 minutes. Then, 50 µL of a 300 mM of p-dimethylaminobenzaldehyde solution in isopropanol was added and mixed thoroughly. The reaction mixture was then exposed to 405 nm LED light for 10 minutes. Following the exposure, absorbance was measured at 560 nm using a Tecan® plate reader.

3.9. Preparation of tryptophan synthase for preparative scale reactions

The cell pellet was resuspended in lysis buffer (approx. 1 g pellet in 5 mL Buffer, 50 mM KP_i, pH 8, 40 µM pyridoxal phosphate (PLP), 2 mM $MgCl_2$) and lysed using an ultrasonic cell disruptor (Branson Sonifier II "Model W-250", Heinemann) for two 10-minute sessions at 35–40% amplitude. The crude lysate was then heated to 70 °C for 30 minutes to remove heat-instable proteins and then chilled on ice. Afterwards, insoluble proteins and cell debris were removed by centrifugation (4 °C, 10,000 × rcf, 20 minutes), and the supernatant was collected as the lysate. If not used immediately, the lysate was frozen in aliquots under liquid nitrogen and stored in a freezer at -20 °C.

3.10. Automated Screening Process

For the automated library screening the previously described AutoBioTech platform as well as an OT-2 liquid handler (Opentrons, USA) were used.[41]

Per library, 66 colonies were picked using a Tecan Fluent (Tecan, Switzerland) with integrated colony picker (Pickolo, SciRobotics, Israel) to inoculate 900 µL LB media in a square well deep well plate (sqDWP). 10 µL of cryo-cultures of an pET21a empty vector as well as pET21a with PsmD wild type were additionally carried on each plate as negative and positive control. After 16 hours at 800 rpm and 37 °C, 10 µL of these pre-cultures were used to inoculate four main cultures per variant in four fresh sqDWPs with 900 µL TB auto-induction media.

After 24 hours of incubation at 37 °C and 800 rpm, the cells were separated via centrifugation (5 minutes at 3500 rpm), the supernatant removed and cells resuspended in 200 µL freshly prepared reaction mixture containing 1 mM of the four respective substrates and 4 mM SAM in KP_i buffer (50 mM, pH 8). The reaction was performed at 40 °C for 7 hours, without shaking. Afterwards, the reaction was quenched and residual cells and enzyme was precipitated using 20 µL 5% TFA followed by a centrifugation step. 100 µL of the supernatant was transferred to a fresh micro titer plate (MTP) and stored at -20 °C until further use.

For determination of the enzymatic activity, first 50 µL of the supernatant were mixed with 50 µL conc. H_2S0_4 and incubated for 30 minutes at room temperature. Afterwards, 100 µL mixture were transferred to a fresh MTP, 50µL pDMAB (300 mM in isopropanol) were added followed by 10 minutes of UV-light exposure (405 nm LED light). Finally, the absorbance at 540 nm was determined in a plate photometer.

4. Appendix

4.1. Additional figures

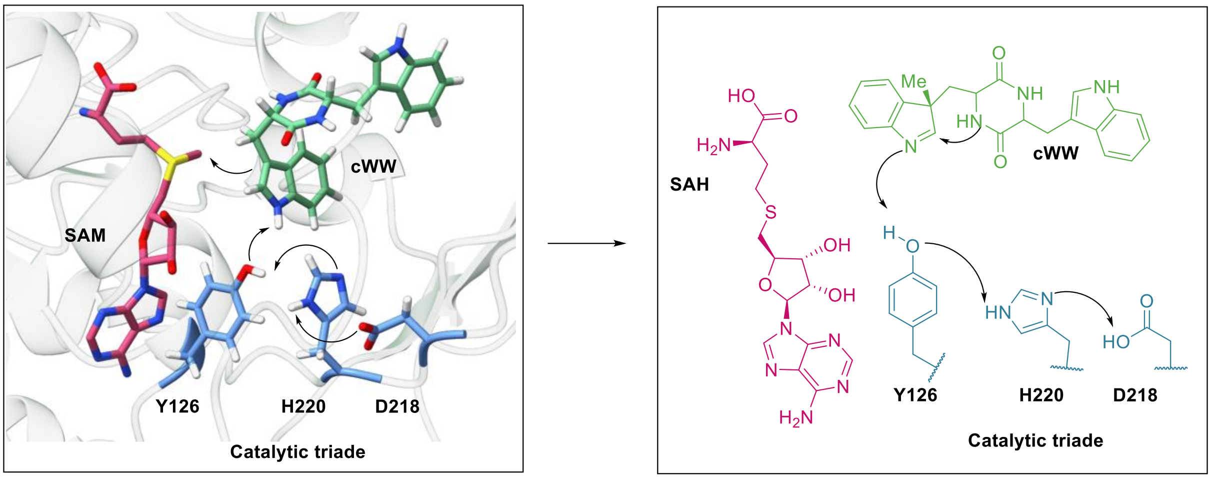

Figure S1: Proposed mechanism for the reaction of SgMT. The cWW substrate is highlighted in green, SAM in pink and the residues of the catalytic triade in blue.

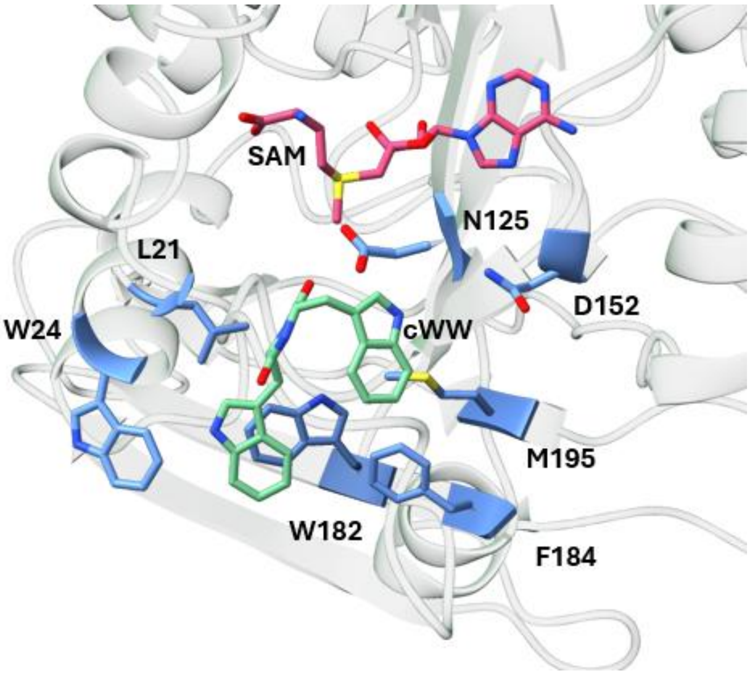

Figure S2: Catalytic side of SgMT (PDB: 9GDJ) with SAM (dark pink), the cWW (green) and the amino acids tested in the alanine scan (blue).[26]

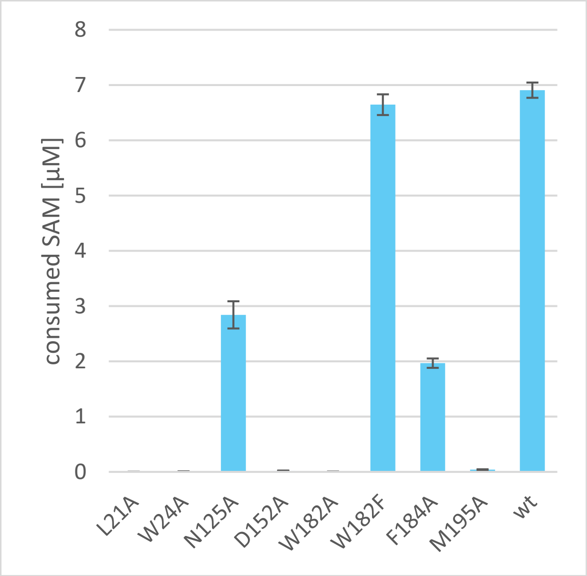

Figure S3: Activity of SgMT variants for the cWW substrate measured with the MTase Glo-Assay (Promega).

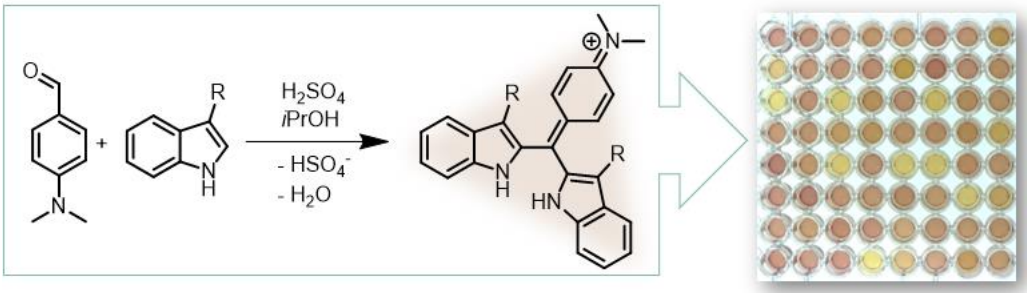

Figure S4: Principle of the indole-Assay: The indolic substrate reacts with the DMAB forming a colorimetric product under acidic conditions in presence of iPrOH.The assay was carried out in a 96 well plate format.

<>	1	2	3	4	5	6	7	8	9
A	1	9	17	25	33	41	49	57	65
B	2	10	18	26	34	42	50	58	66
C	3	11	19	27	35	43	51	59	Positive
D	4	12	20	28	36	44	52	60	Positive
E	5	13	21	29	37	45	53	61	Negative
F	6	14	22	30	38	46	54	62	Negative
G	7	15	23	31	39	47	55	63	
H	8	16	24	32	40	48	56	64	

Figure S5: Layout of the 96 well plate format for the mutagenesis study: 66 variants were tested plus two negative (empty vector) and two positive contols (wt).

<>	1	2	3	4	5	6	7	8	9
A	0,340	0,324	0,323	0,321	0,320	0,308	0,315	0,313	0,337
B	0,316	0,332	0,322	0,314	0,324	0,348	0,323	0,326	0,346
C	0,266	0,329	0,257	0,319	0,313	0,267	0,300	0,315	0,196
D	0,331	0,341	0,302	0,322	0,325	0,303	0,316	0,301	0,195
E	0,416	0,327	0,289	0,312	0,203	0,291	0,315	0,326	0,325

F	0,355	0,367	0,319	0,322	0,327	0,314	0,234	0,295	0,328
G	0,330	0,329	0,327	0,331	0,324	0,312	0,299	0,316	
H	0,339	0,338	0,338	0,204	0,334	0,328	0,308	0,313	

Figure S6: Results of the mutagenesis study for position N125. The indole assay was used to form the colorimetric product, which was measured via absorption at 540 nm.

<>	1	2	3	4	5	6	7	8	9
A	0,334	0,343	0,345	0,297	0,325	0,301	0,321	0,281	0,313
B	0,329	0,329	0,350	0,306	0,296	0,296	0,258	0,282	0,316
C	0,346	0,297	0,316	0,329	0,312	0,313	0,292	0,261	0,183
D	0,346	0,346	0,348	0,308	0,298	0,294	0,317	0,267	0,181
E	0,348	0,299	0,315	0,284	0,296	0,293	0,332	0,285	0,325
F	0,362	0,323	0,307	0,351	0,304	0,309	0,361	0,302	0,350
G	0,363	0,316	0,321	0,290	0,321	0,315	0,343	0,304	
H	0,341	0,344	0,358	0,304	0,294	0,324	0,319	0,291	

Figure S7: Results of the mutagenesis study for position W182. The indole assay was used to form the colorimetric product, which was measured via absorption at 540 nm.

<>	1	2	3	4	5	6	7	8	9
A	0,160	0,221	0,154	0,288	0,164	0,193	0,177	0,172	0,232
B	0,700	0,234	0,199	0,166	0,204	0,240	0,208	0,233	0,259
C	0,326	0,248	0,271	0,252	0,217	0,255	0,283	0,237	0,174
D	0,278	0,232	0,249	0,223	0,293	0,275	0,259	0,233	0,173
E	0,276	0,223	0,256	0,173	0,194	0,208	0,244	0,215	0,263
F	0,206	0,177	0,259	0,217	0,251	0,275	0,204	0,164	0,282
G	0,285	0,240	0,182	0,222	0,228	0,230	0,241	0,235	
H	0,188	0,232	0,230	0,218	0,161	0,276	0,256	0,219	

Figure S8: Results of the mutagenesis study for position F184. The indole assay was used to form the colorimetric product, which was measured via absorption at 540 nm.

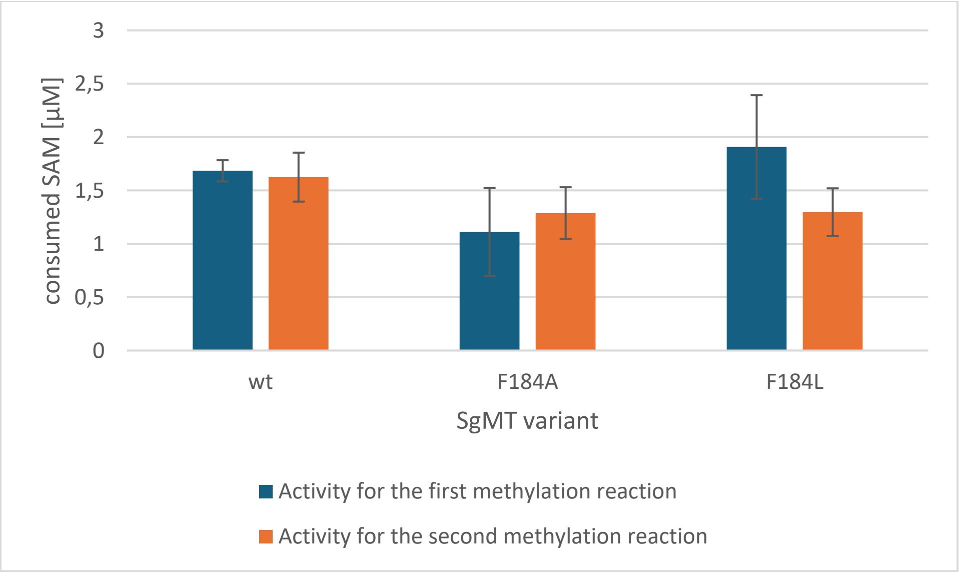

Figure S9: Activity of SgMT variants for the 5-Bromo-cWW substrate (blue) and the single methylated 5-Bromo-cWW intermediate (orange) measured with the MTase Glo-Assay (Promega).

4.2. NMR-spectra

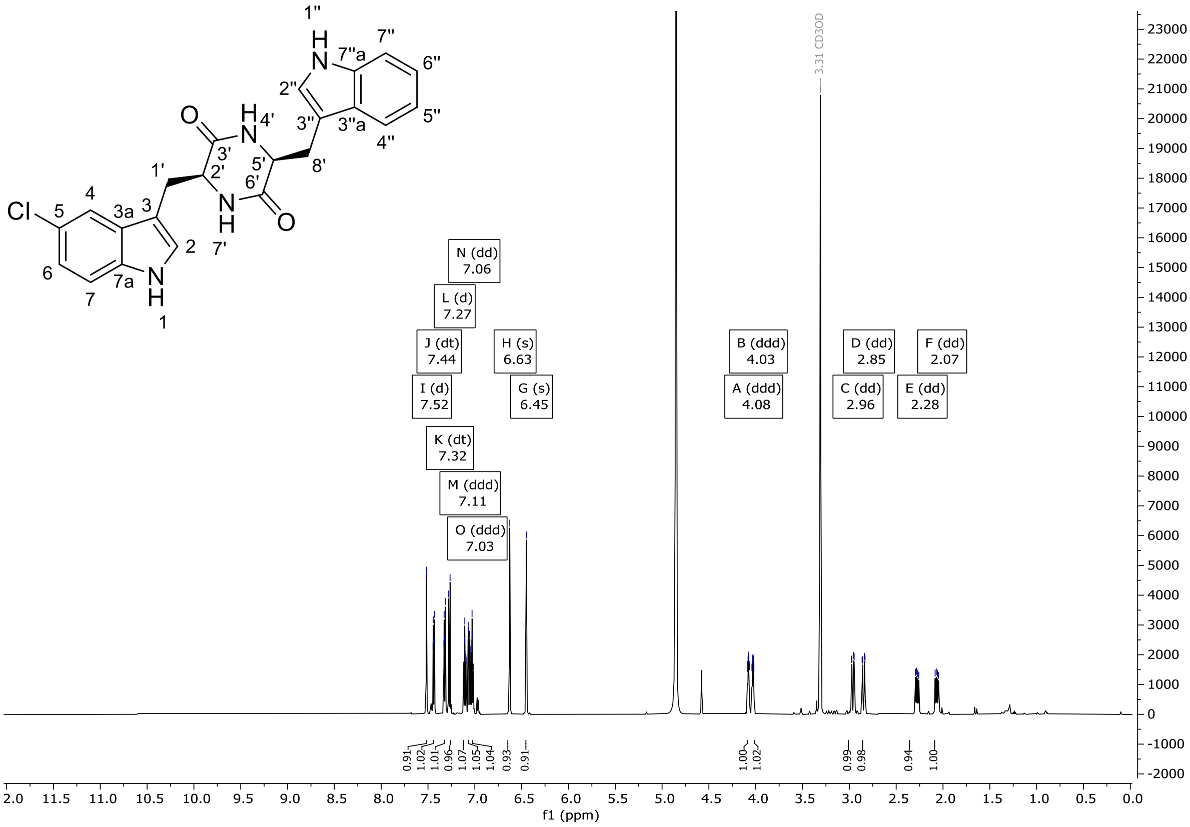

Figure 10: 1H-NMR-spectrum (600 MHz) of **13c** in CD_3OD.

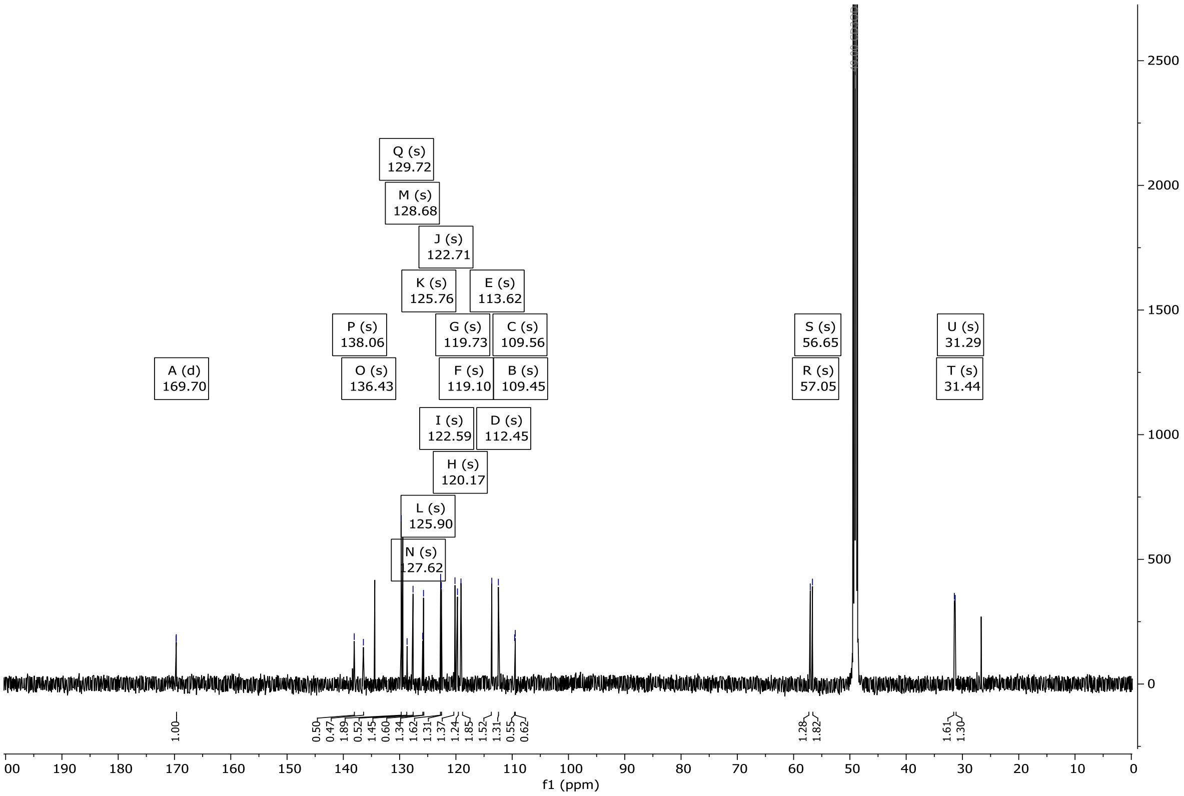

Figure S11: ^{13}C-NMR-spectrum (151 MHz) of **13c** in CD_3OD.

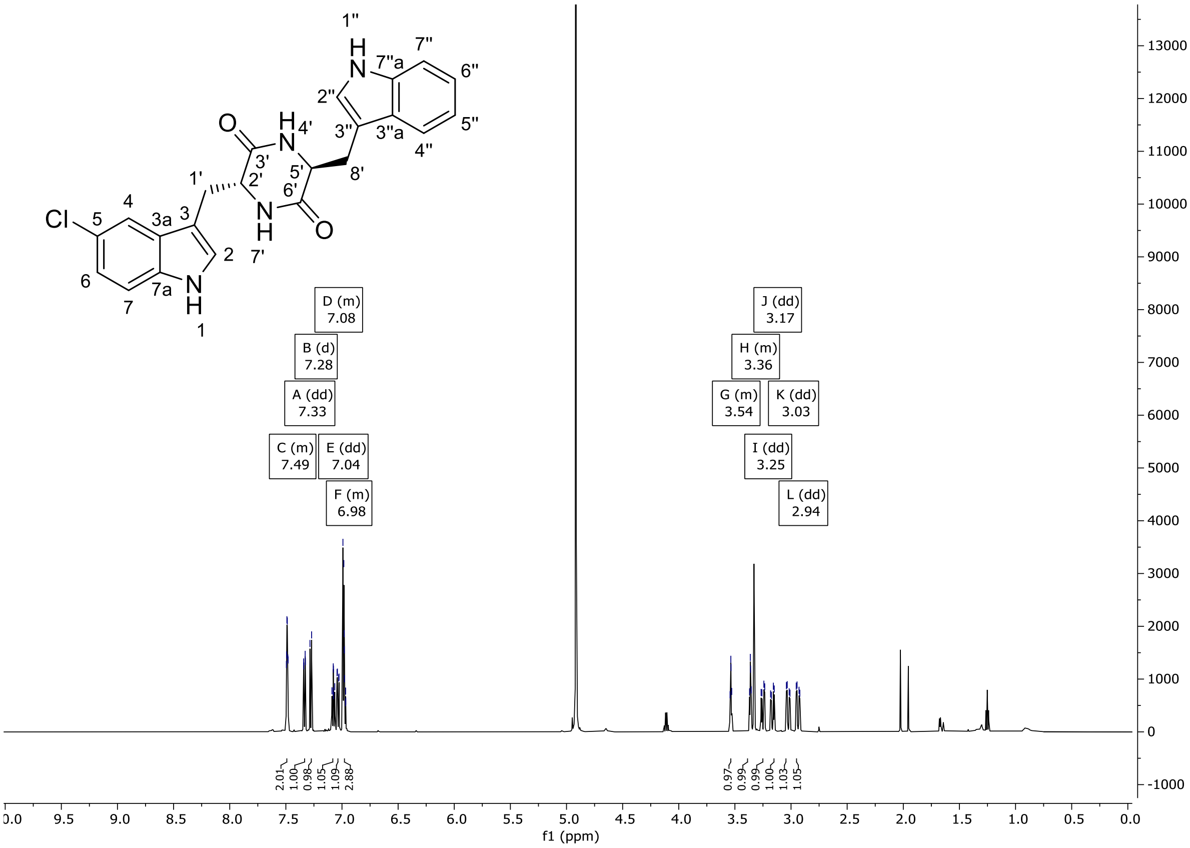

Figure S12: ^{1}H-NMR-spectrum (600 MHz) of **13f** in CD_3OD.

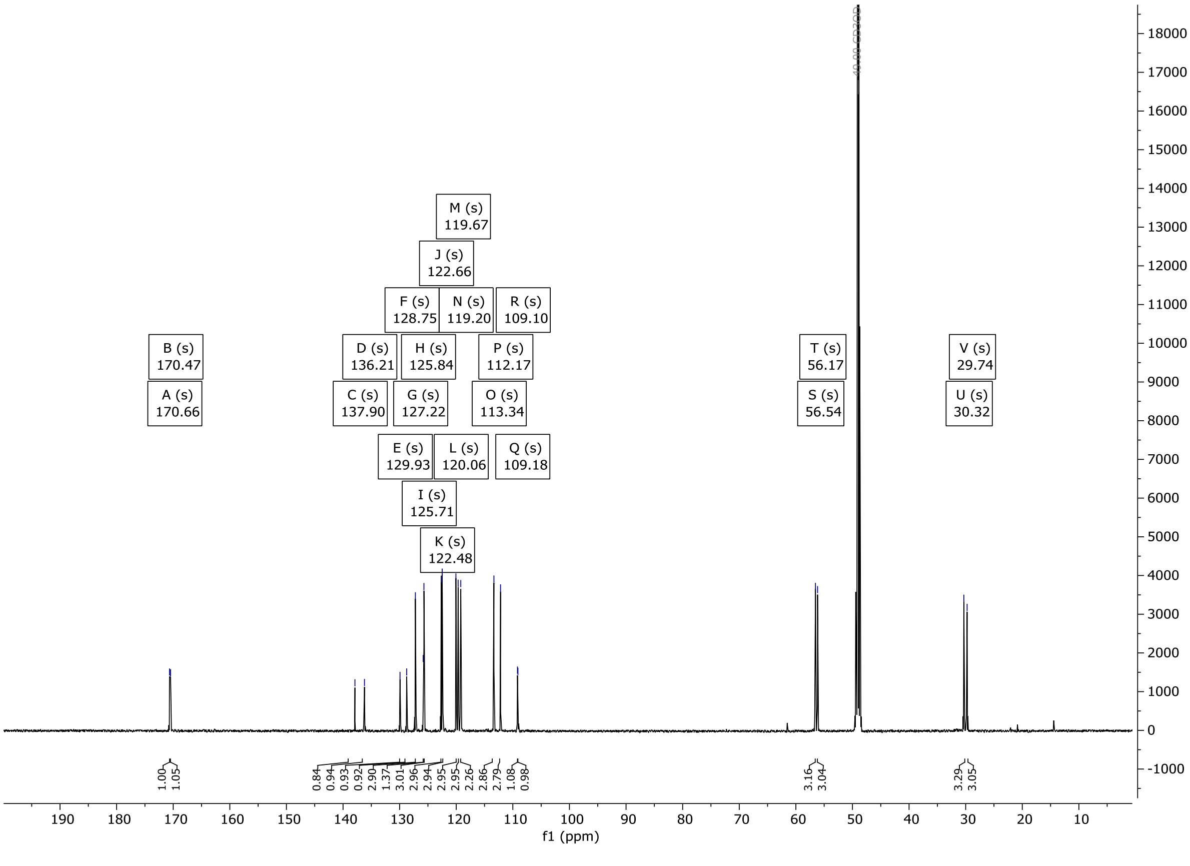

Figure S13: ^{13}C-NMR-spectrum (151 MHz) of **13f** in CD_3OD.

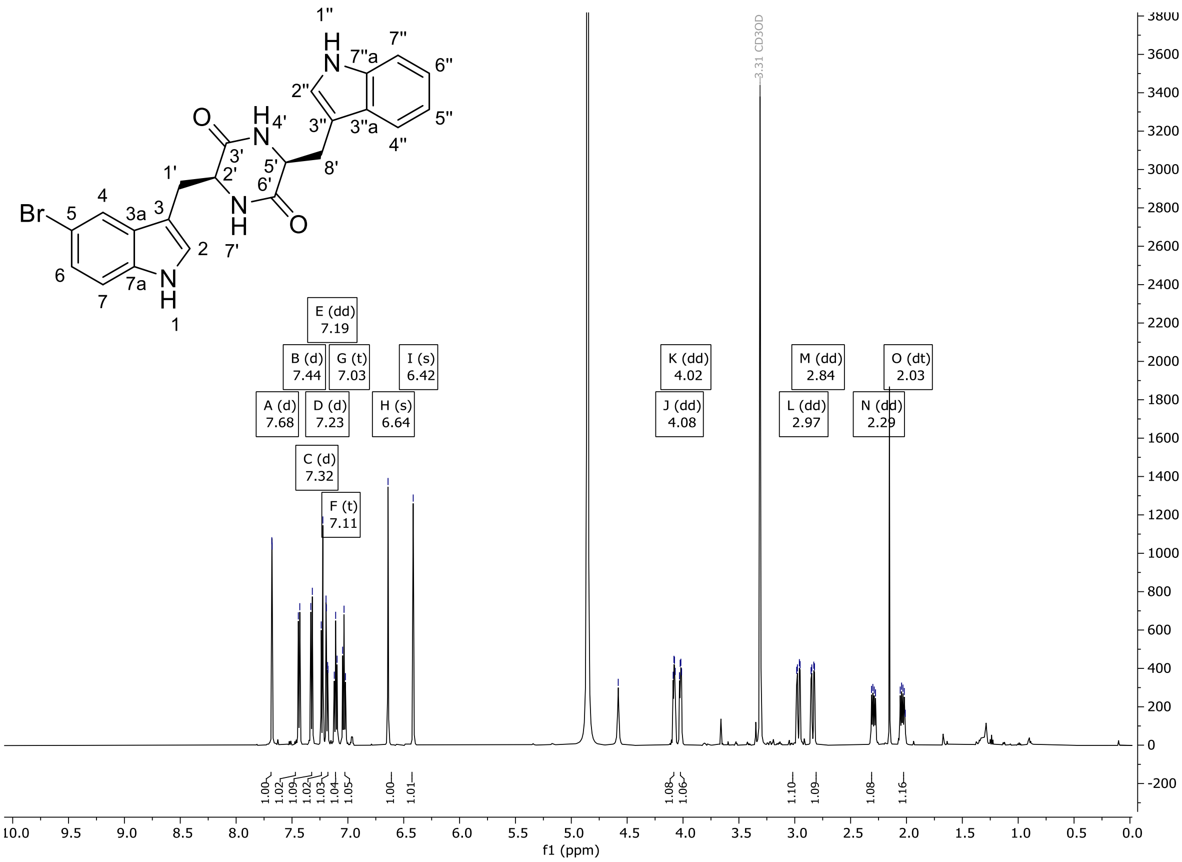

Figure S14: ^{1}H-NMR-spectrum (600 MHz) of **13a** in CD_3OD.

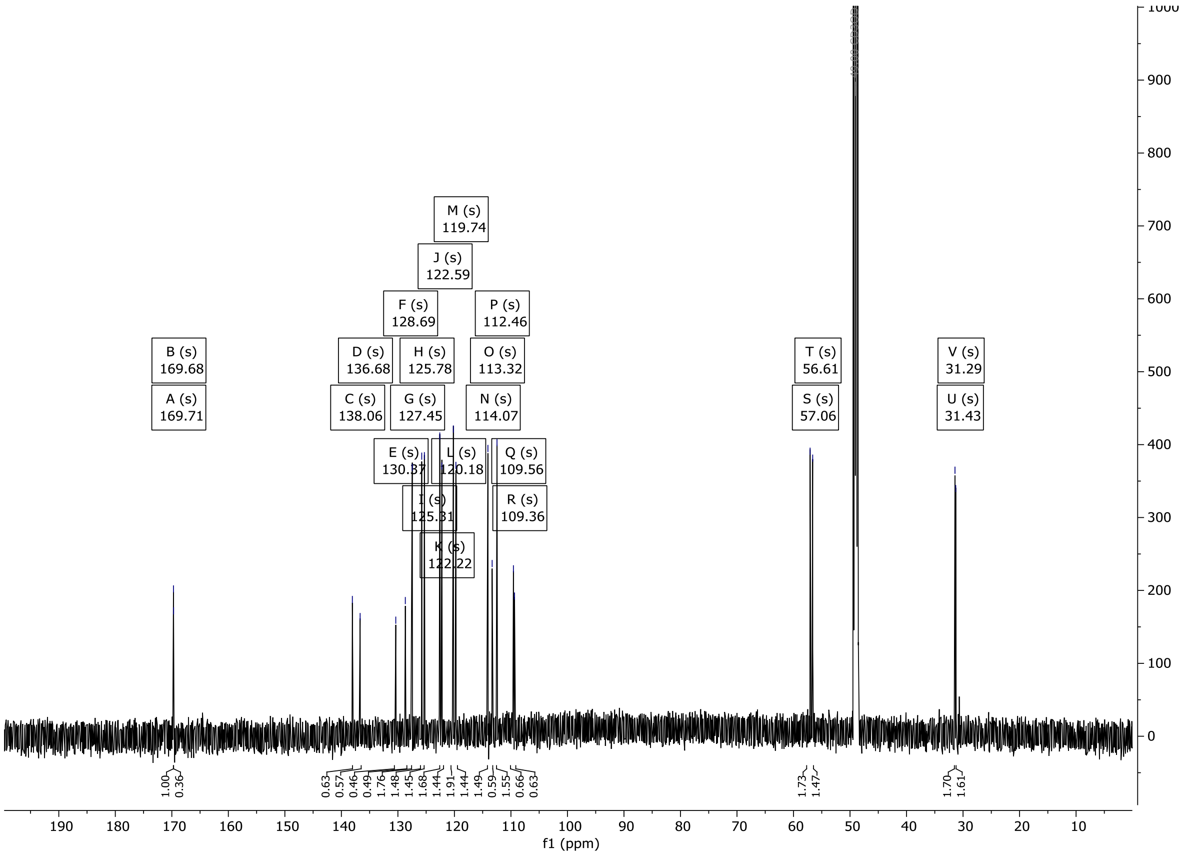

Figure S15: ^{13}C-NMR-spectrum (151 MHz) of **13a** in CD_3OD.

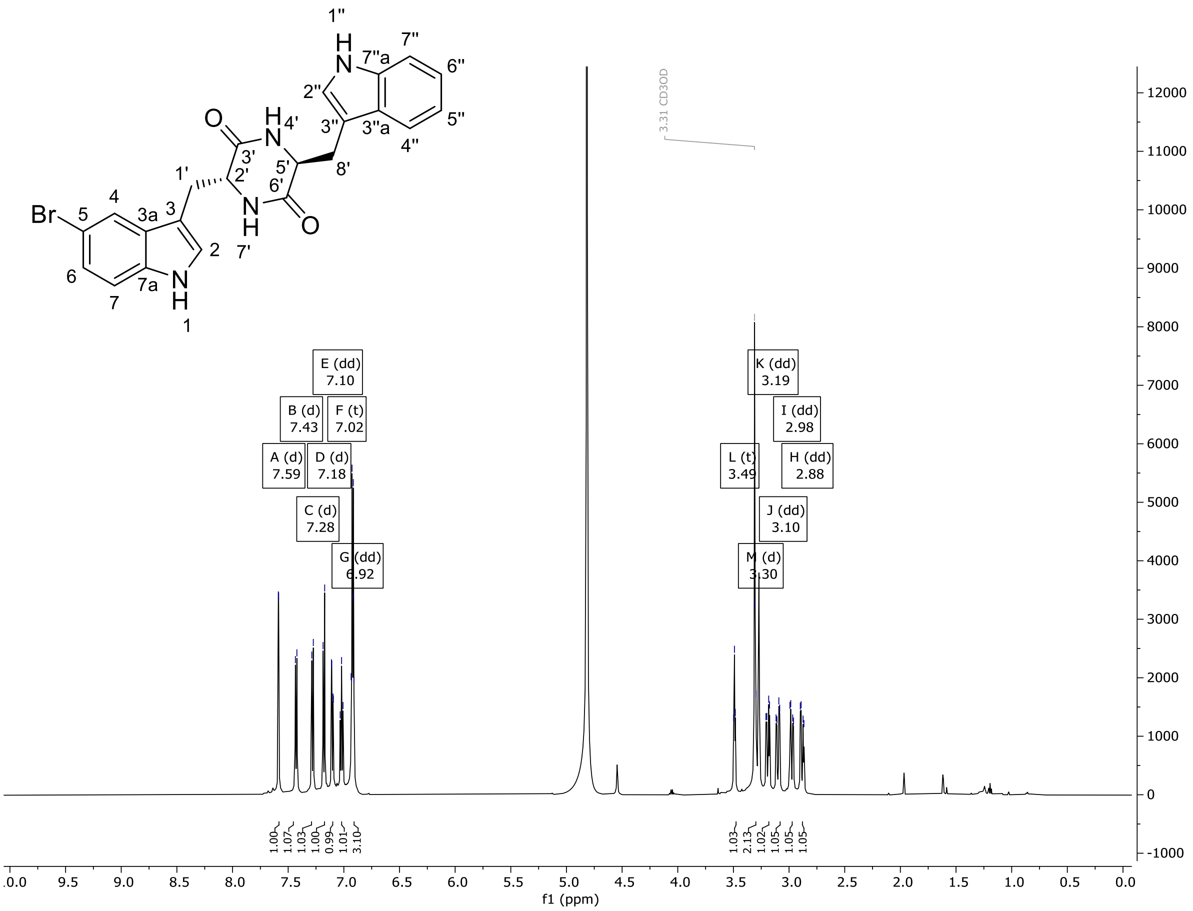

Figure S16: ^{1}H-NMR-spectrum (600 MHz) of **13e** in CD_3OD.

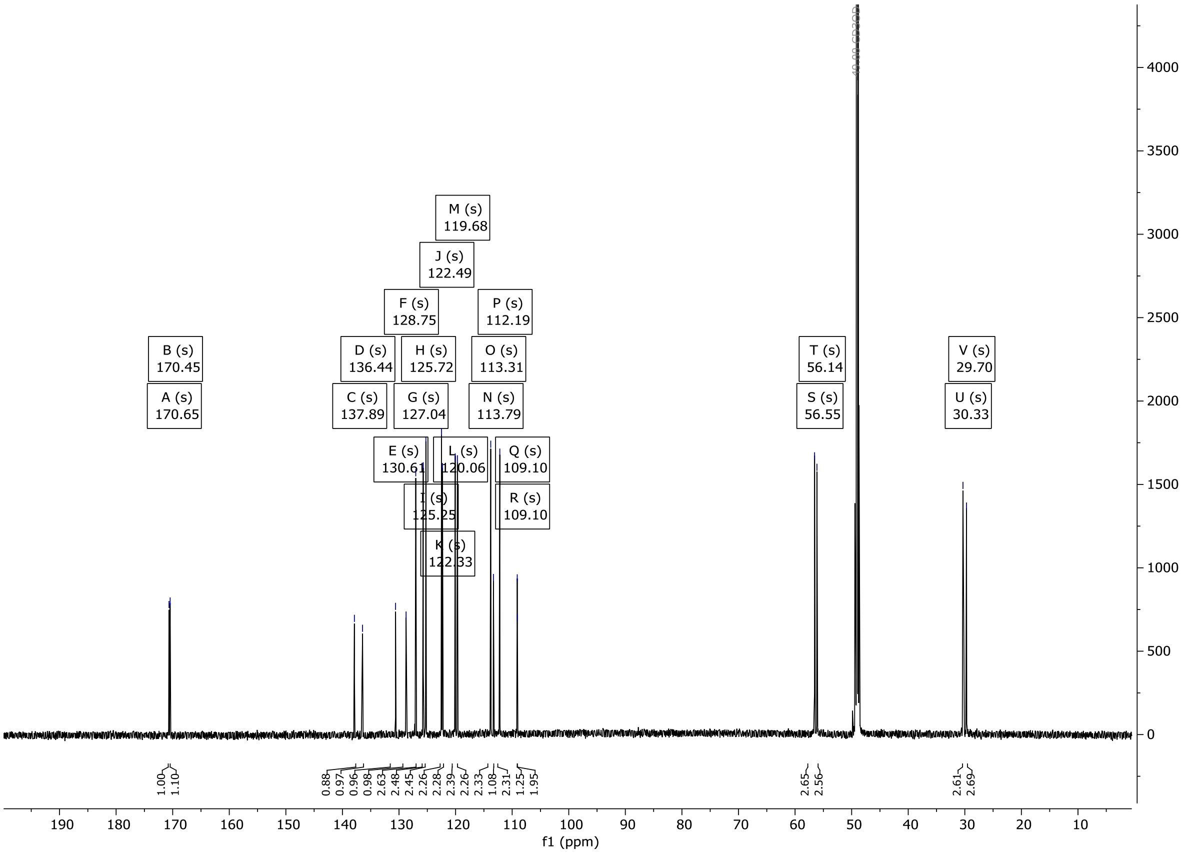

Figure S17: ^{13}C-NMR-spectrum (151 MHz) of **13e** in CD_3OD.

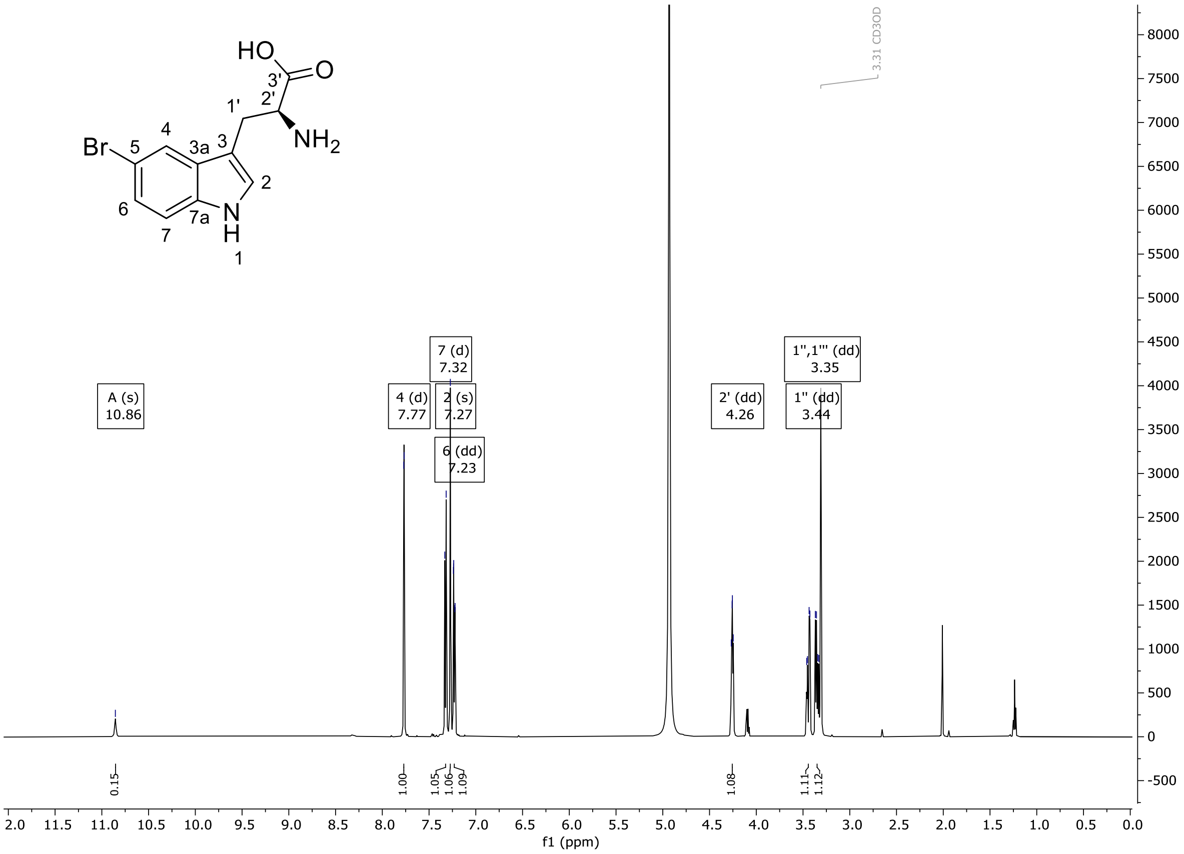

Figure S18: ^{1}H-NMR-spectrum (600 MHz) of **12** in CD_3OD.

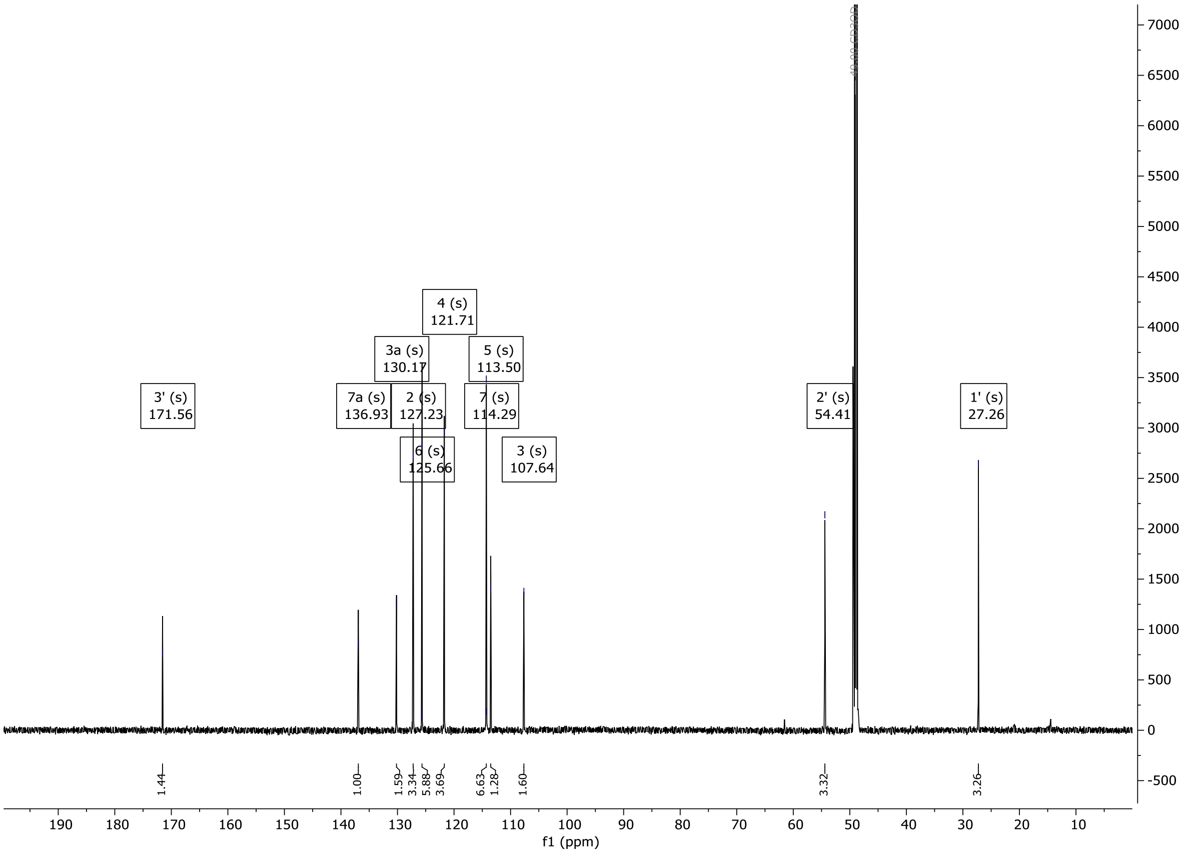

Figure S19: ^{13}C-NMR-spectrum (151 MHz) of **12** in CD_3OD.

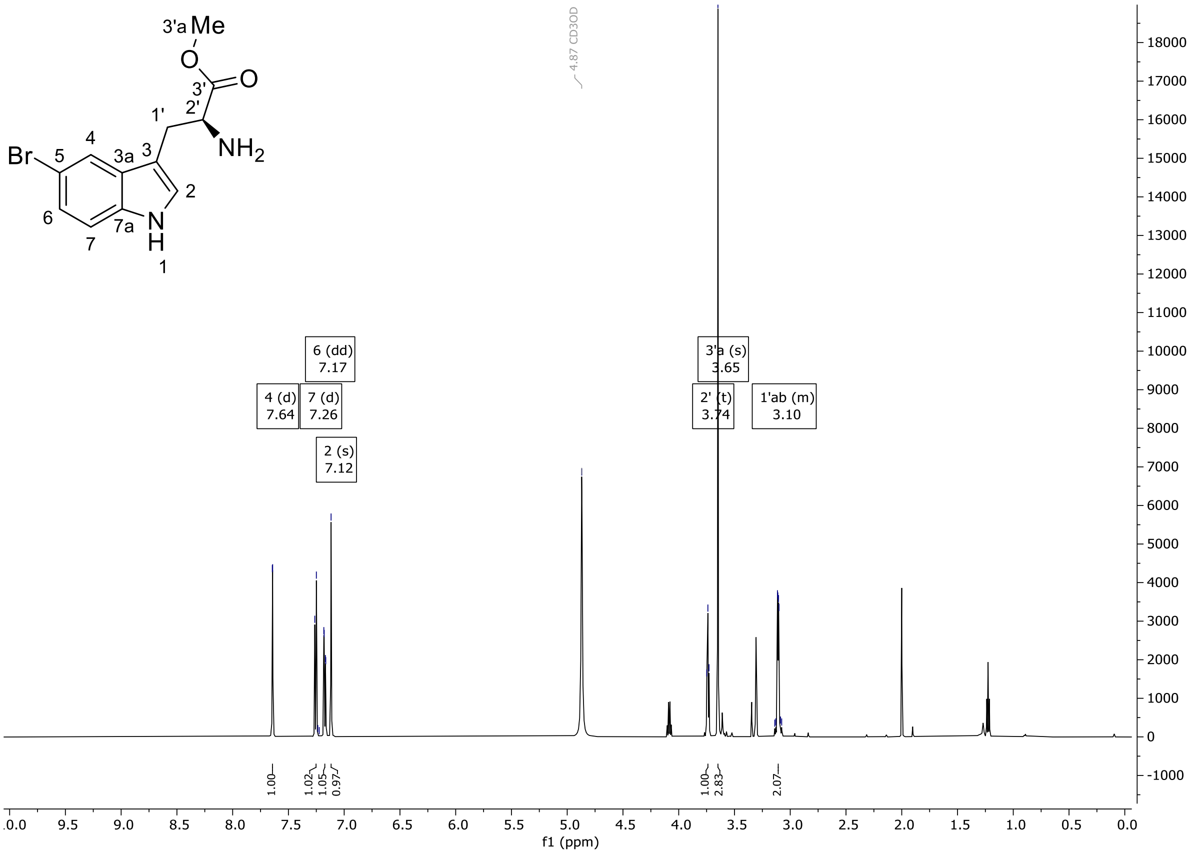

Figure S20: ^{1}H-NMR-spectrum (600 MHz) of **S1** in CD_3OD.

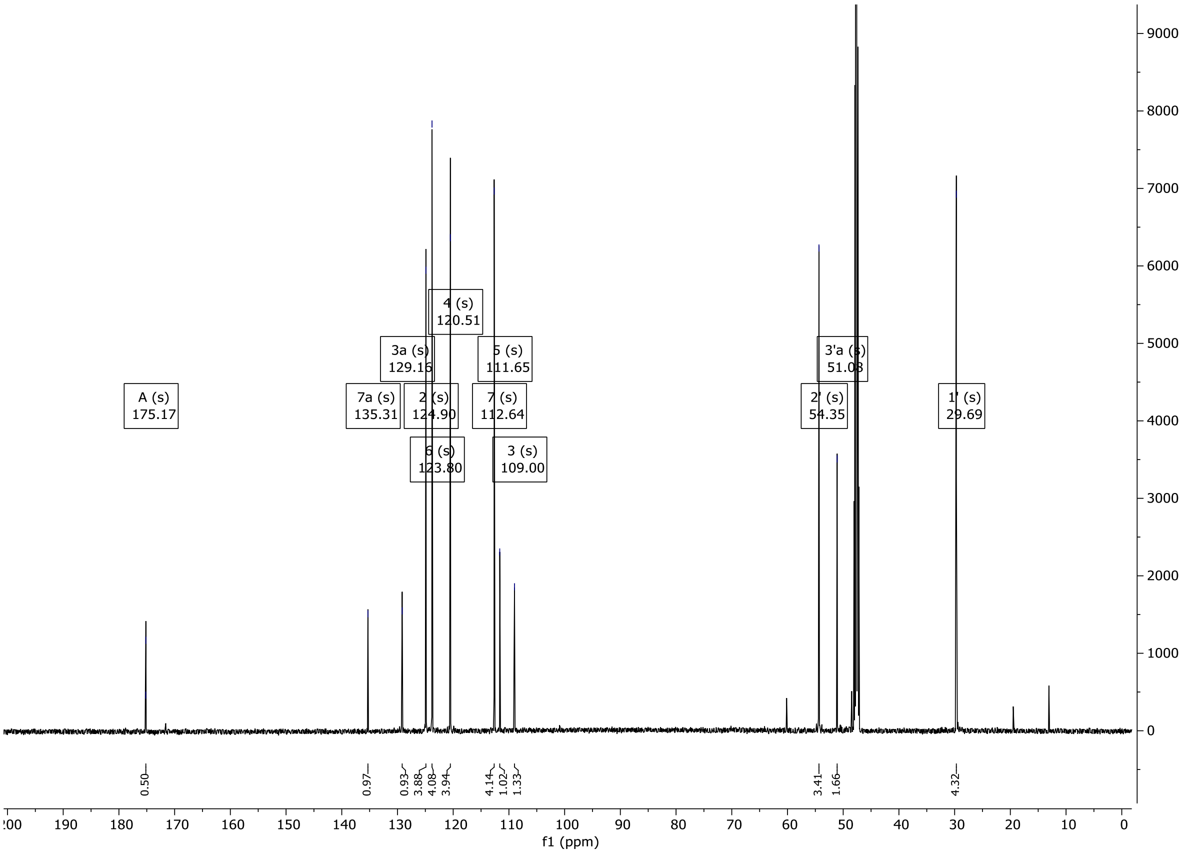

Figure S21: ^{13}C-NMR-spectrum (151 MHz) of **S1** in CD_3OD.

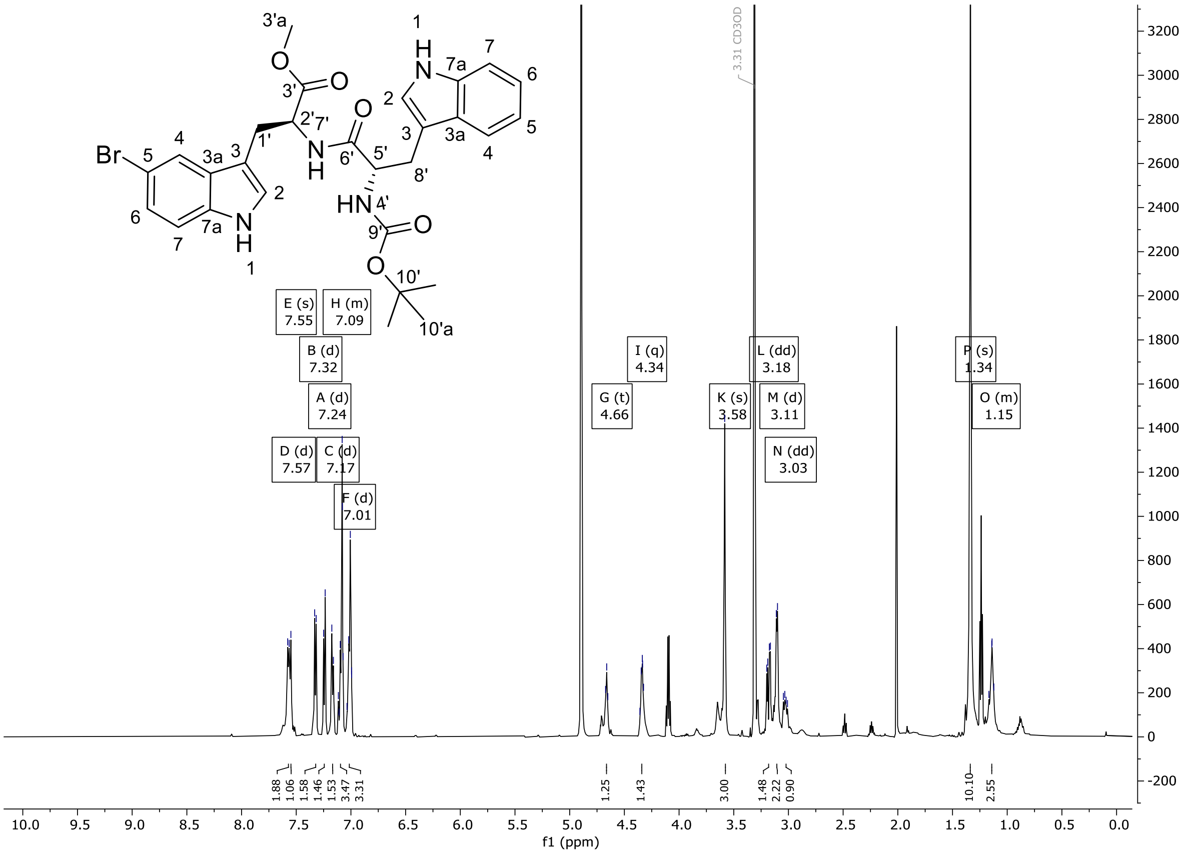

Figure S22: ^{1}H-NMR-spectrum (600 MHz) of **S2** in CD_3OD.

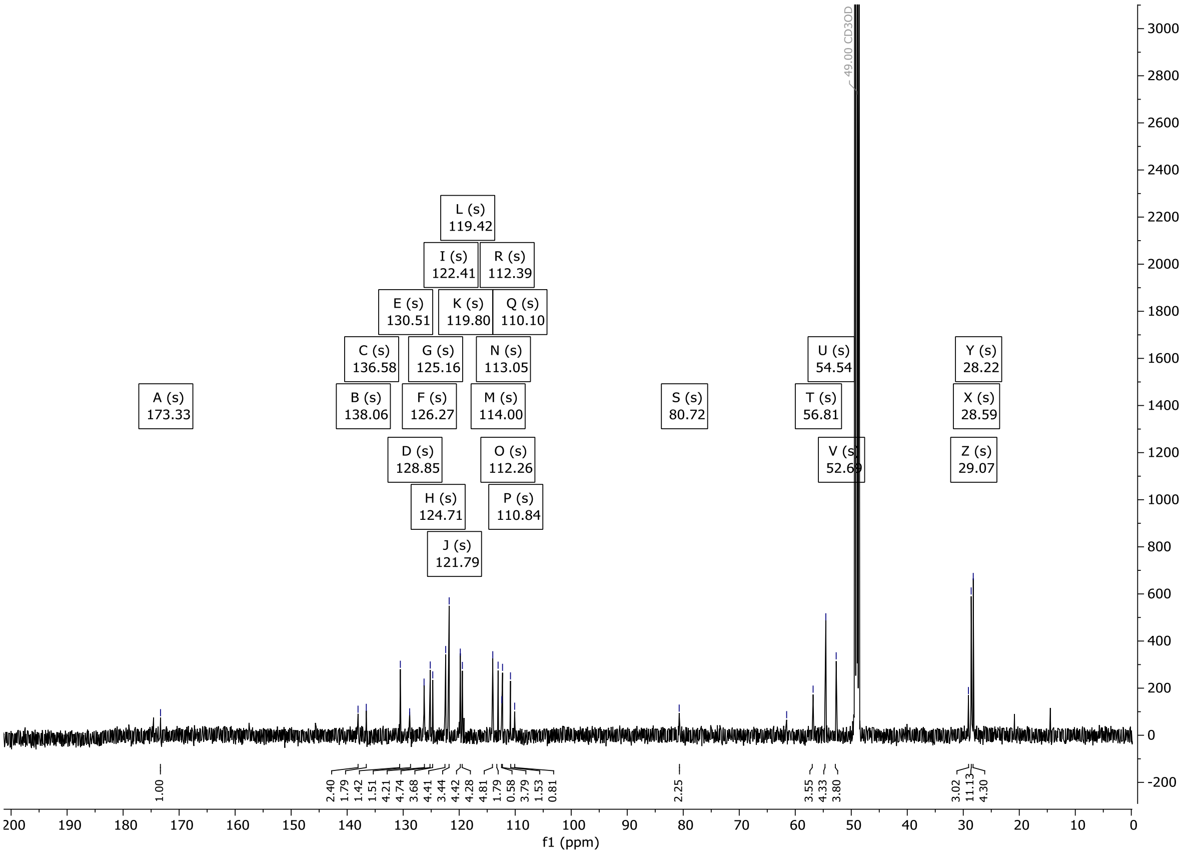

Figure S23: ^{13}C-NMR-spectrum (151 MHz) of **S2** in CD_3OD.

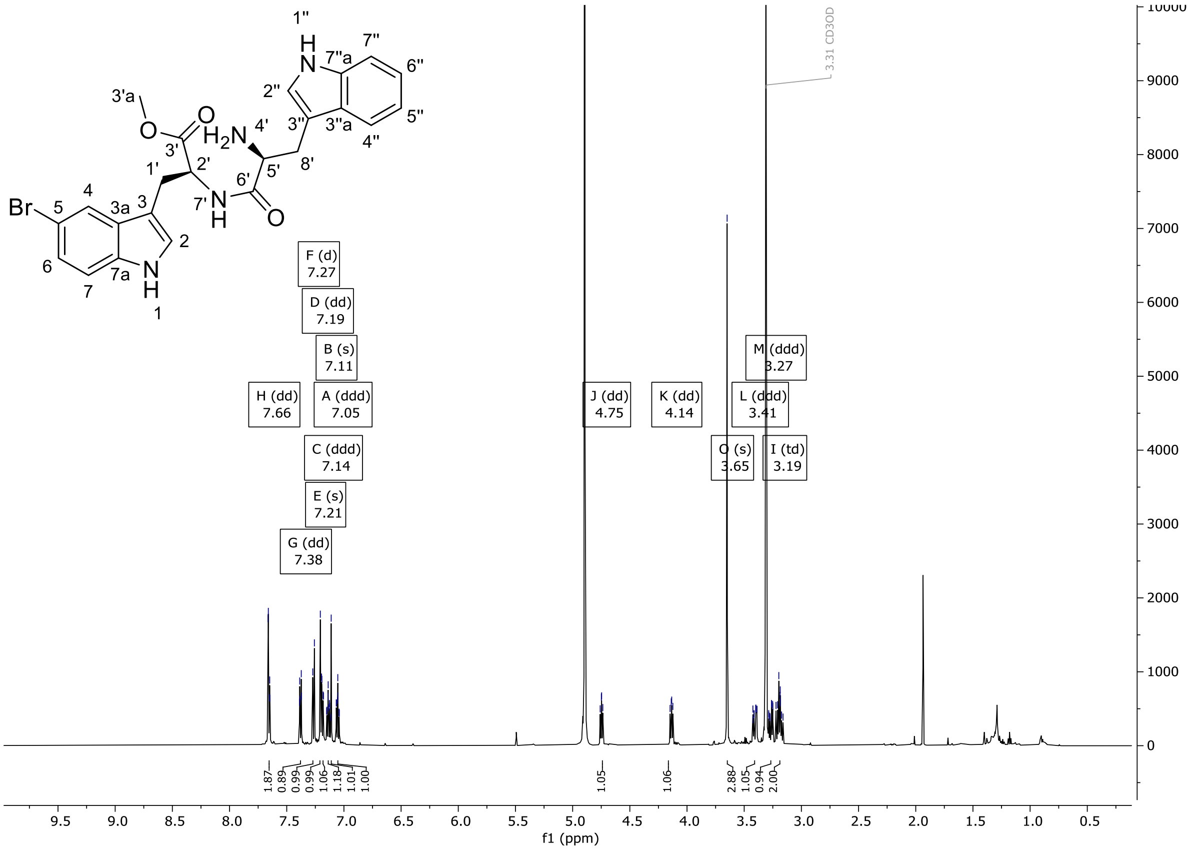

Figure S24: Crude ^{1}H-NMR-spectrum (600 MHz) of **S3** in CD_3OD.

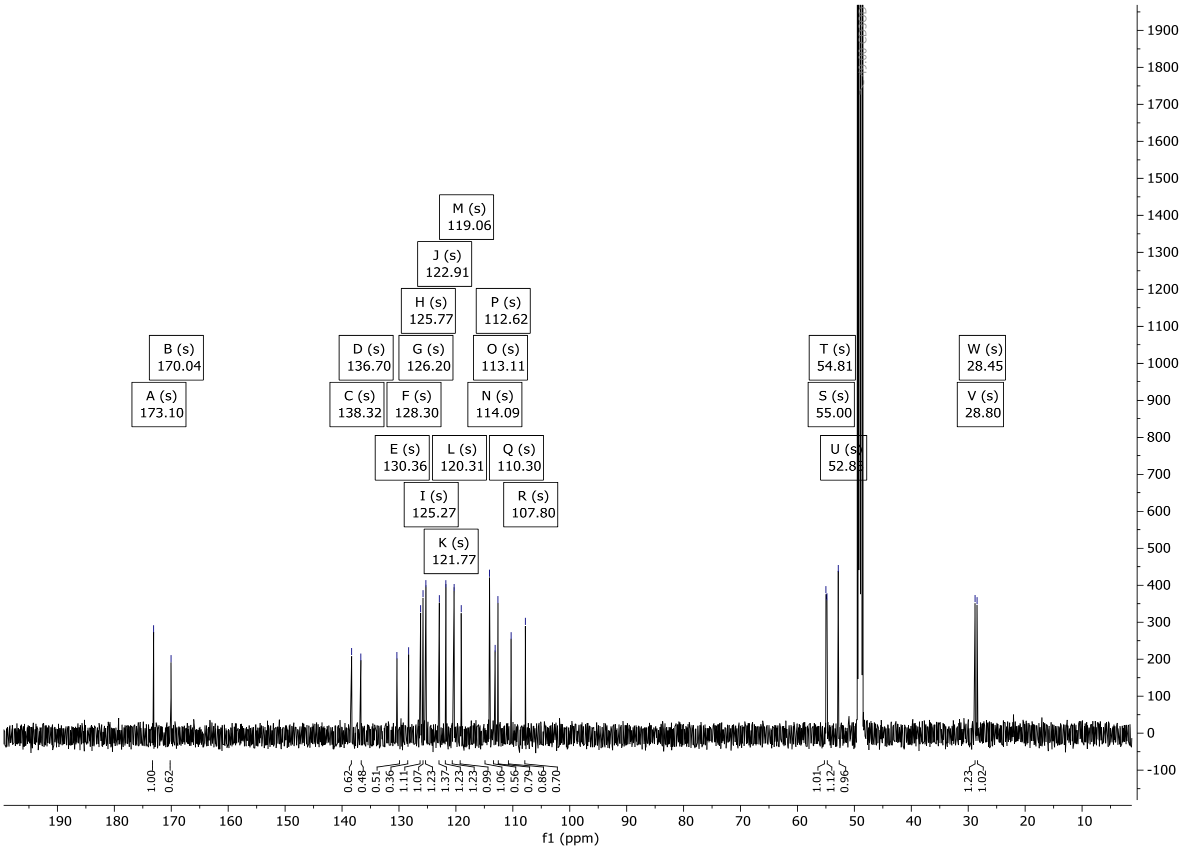

Figure 25: Crude ^{13}C-NMR-spectrum (151 MHz) of **S3** in CD_3OD.

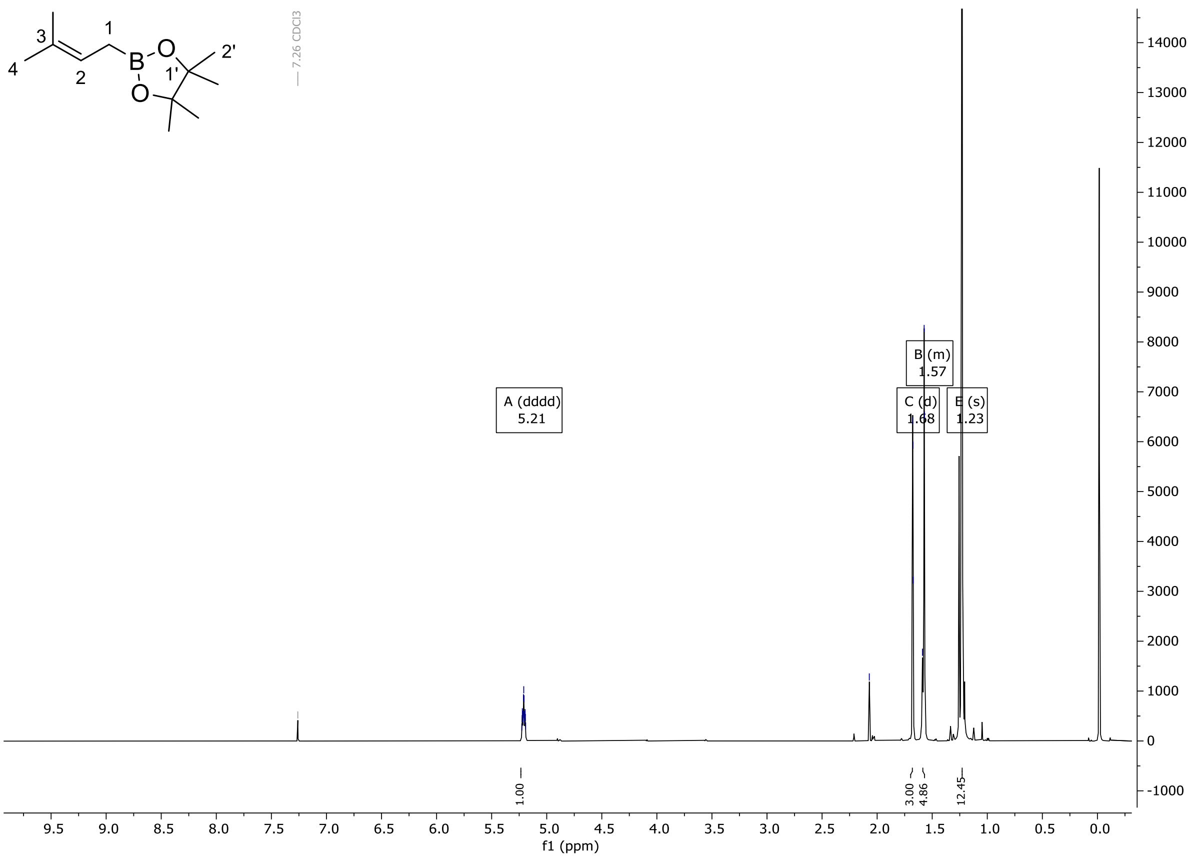

Figure S26: ^{1}H-NMR-spectrum (600 MHz) of **14** in $CDCl_3$.

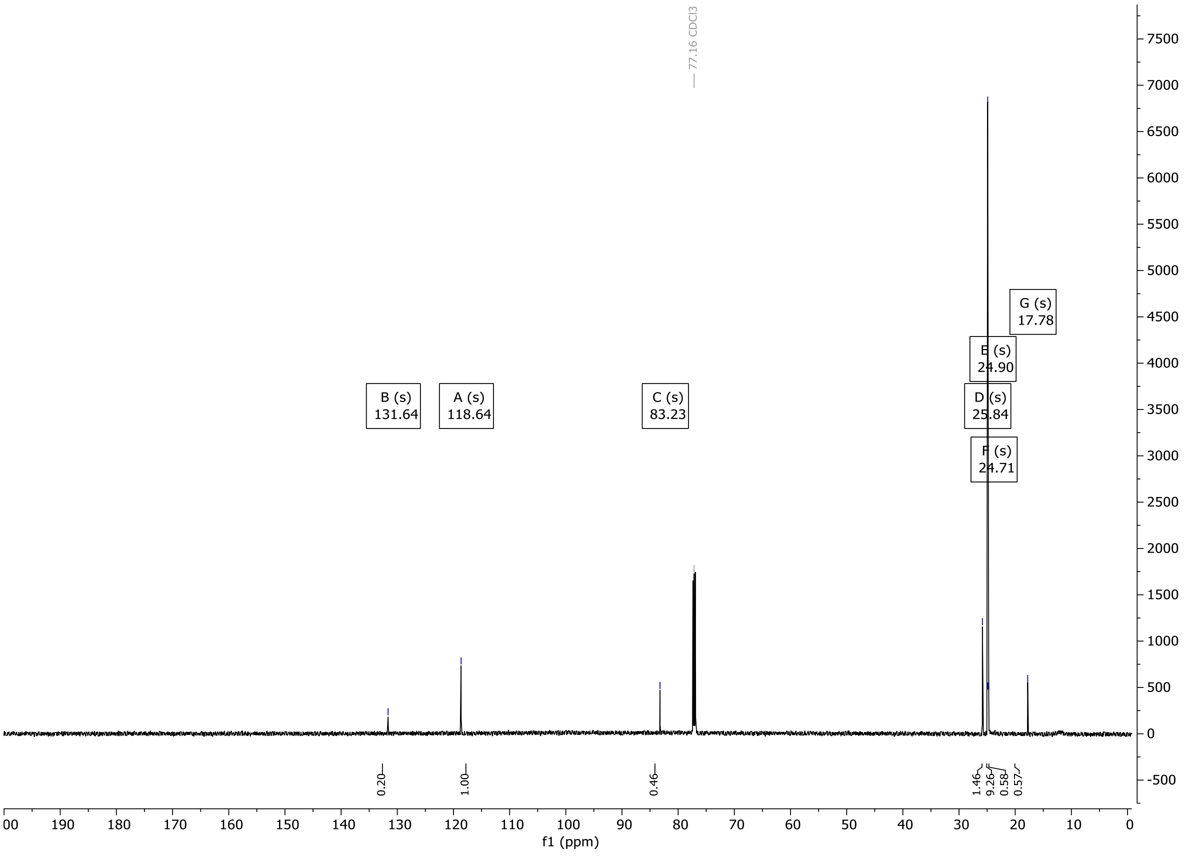

Figure S27: ^{13}C-NMR-spectrum (151 MHz) of **14** in $CDCl_3$.

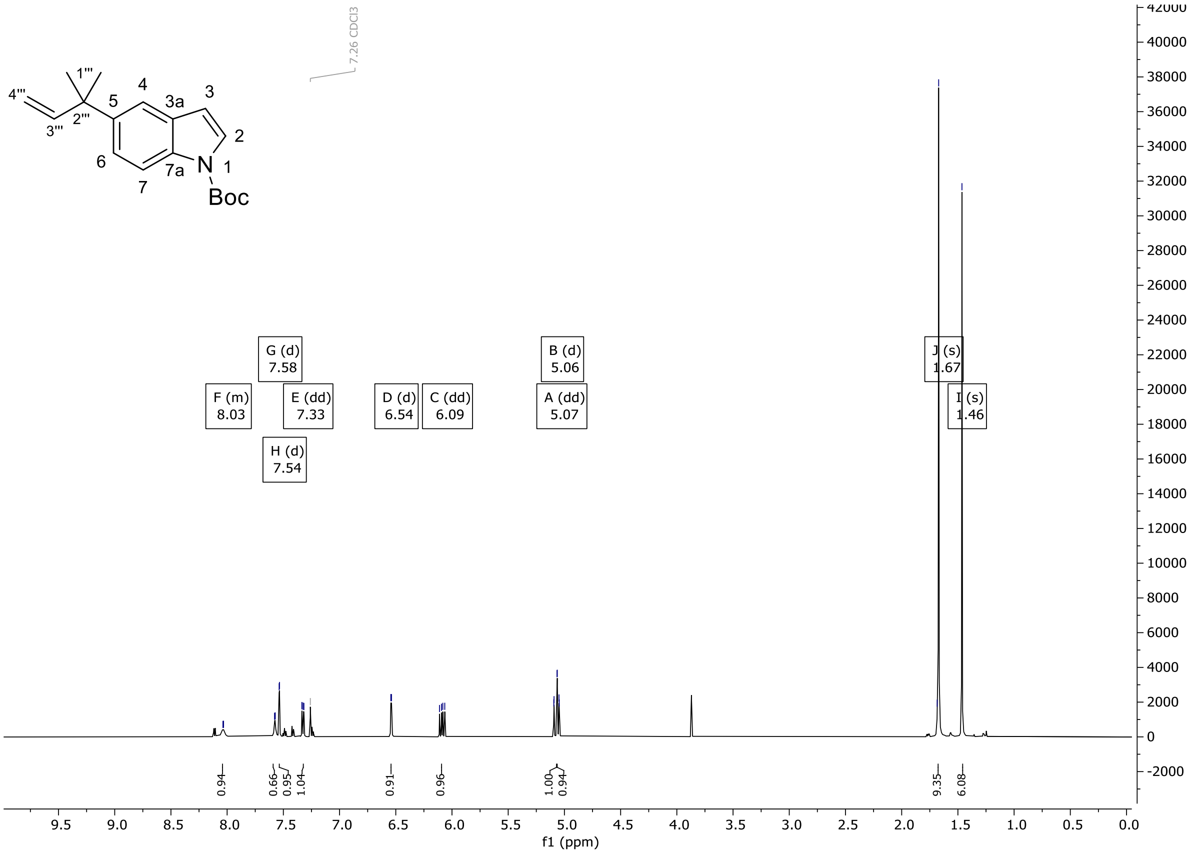

Figure S28: Crude ^{1}H-NMR-spectrum (600 MHz) of **S6** in $CDCl_3$.

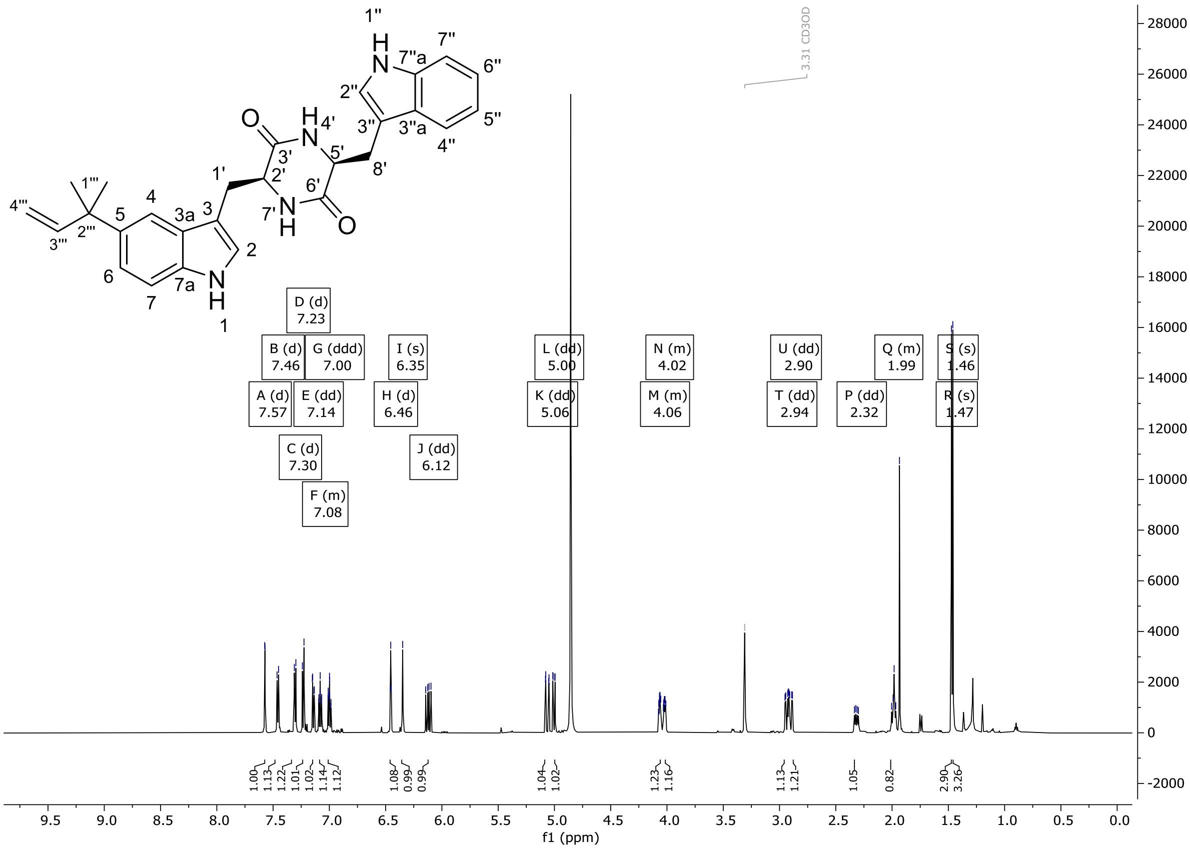

Figure S29 : ^{1}H-NMR-spectrum (600 MHz) of **13b** in CD_3OD.

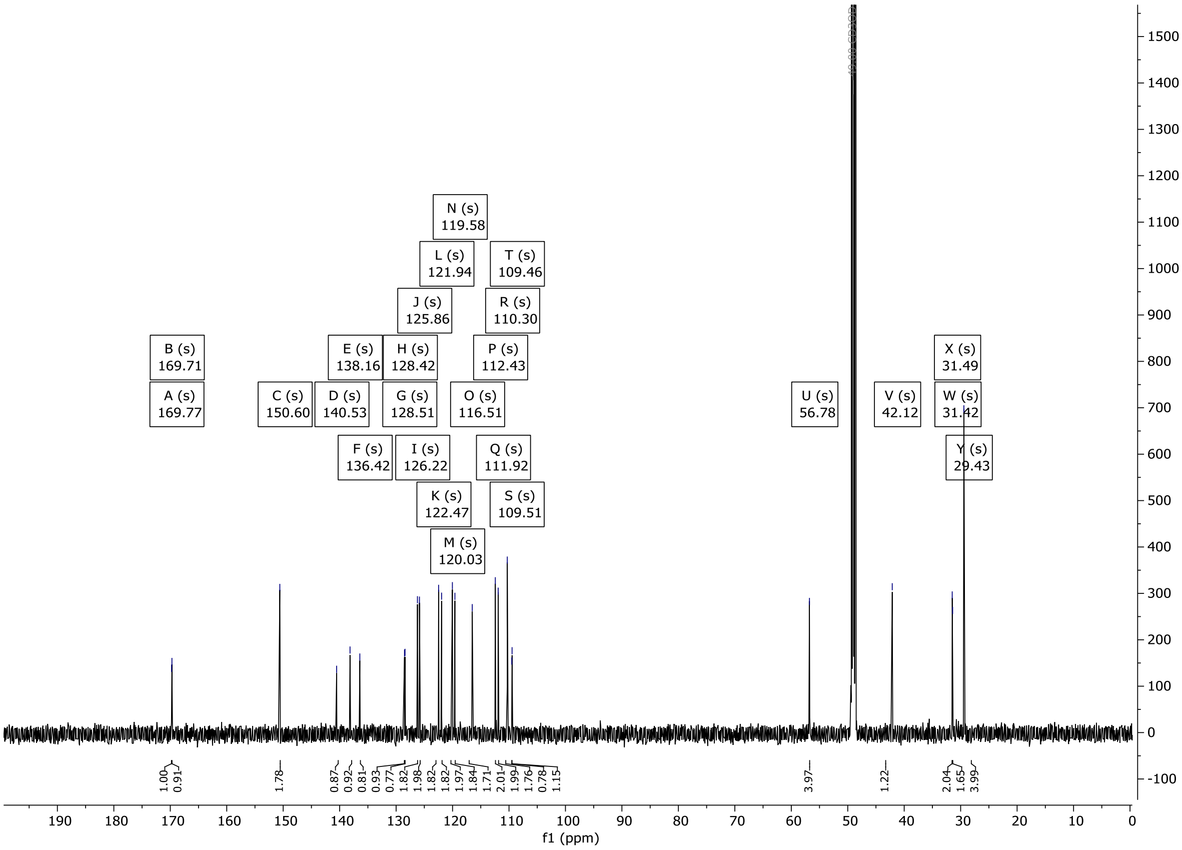

Figure S30: ^{13}C-NMR-spectrum (151 MHz) of **13b** in CD_3OD.

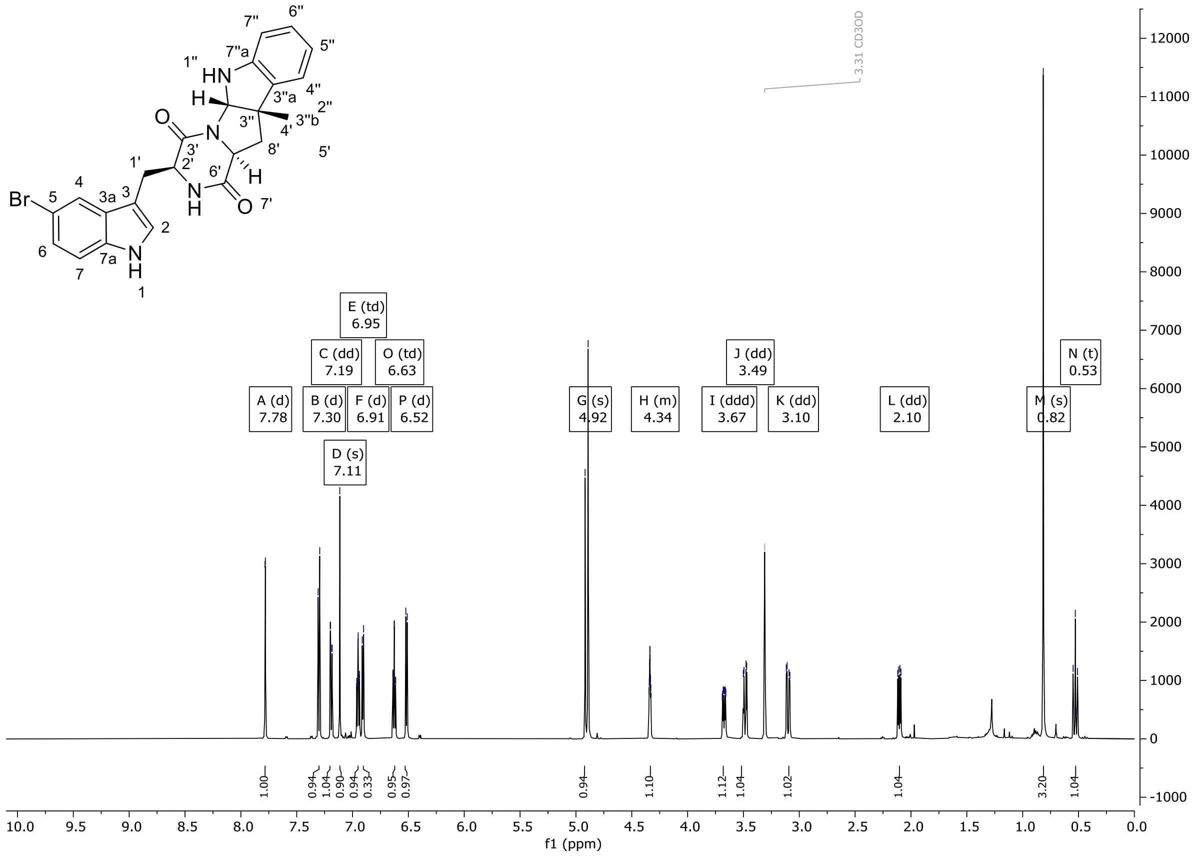

Figure S31: ^{1}H-NMR-spectrum (600 MHz) of **17a** in CD_3OD.

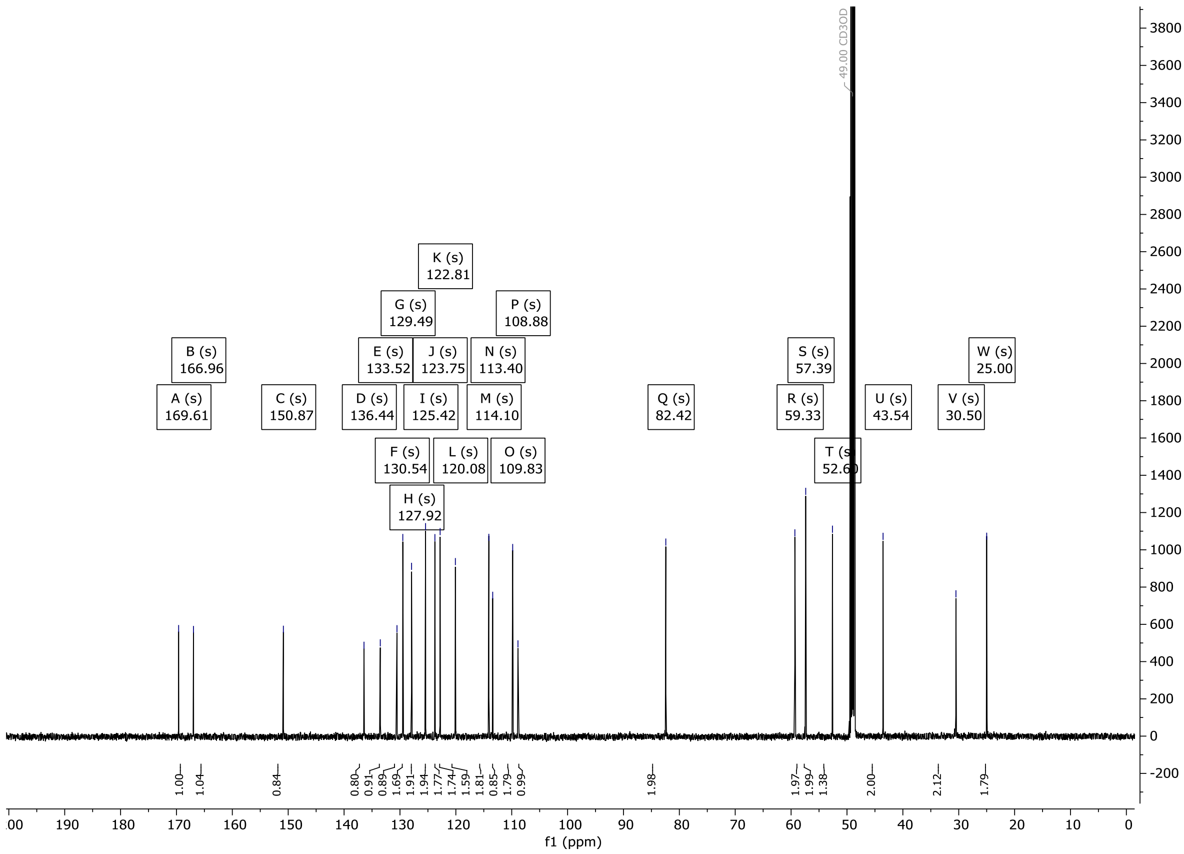

Figure S32: ^{13}C-NMR-spectrum (151 MHz) of **17a** in CD_3OD.

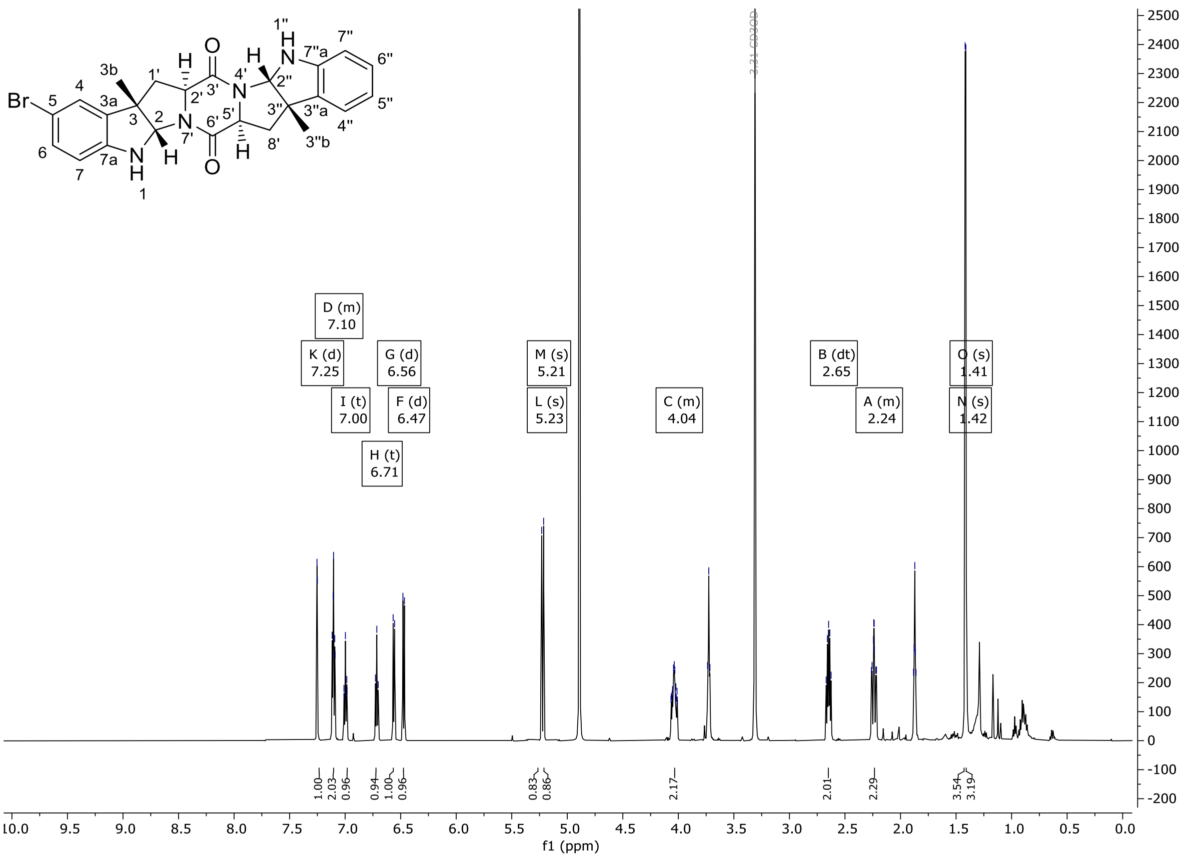

Figure S33: ^{1}H-NMR-spectrum (600 MHz) of **18a** in CD_3OD.

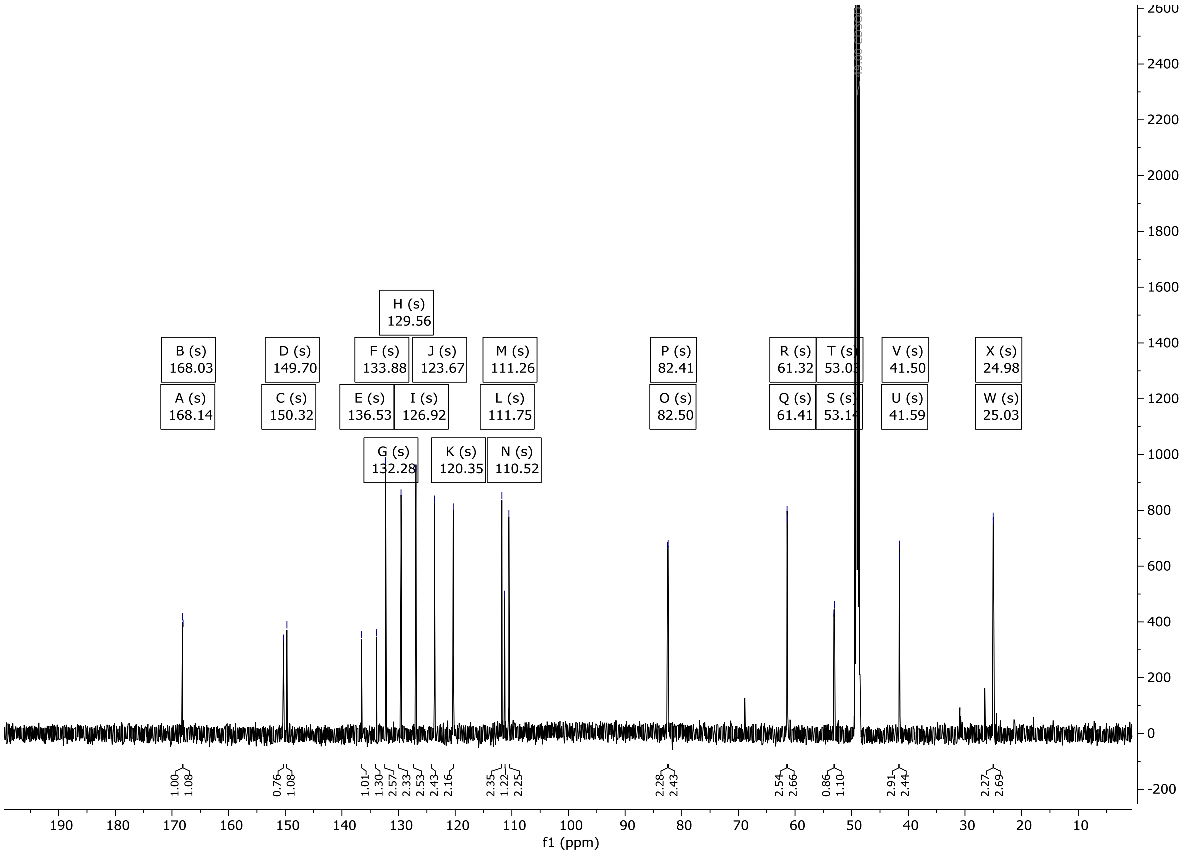

Figure S34: ^{13}C-NMR-spectrum (151 MHz) of **18a** in CD_3OD.

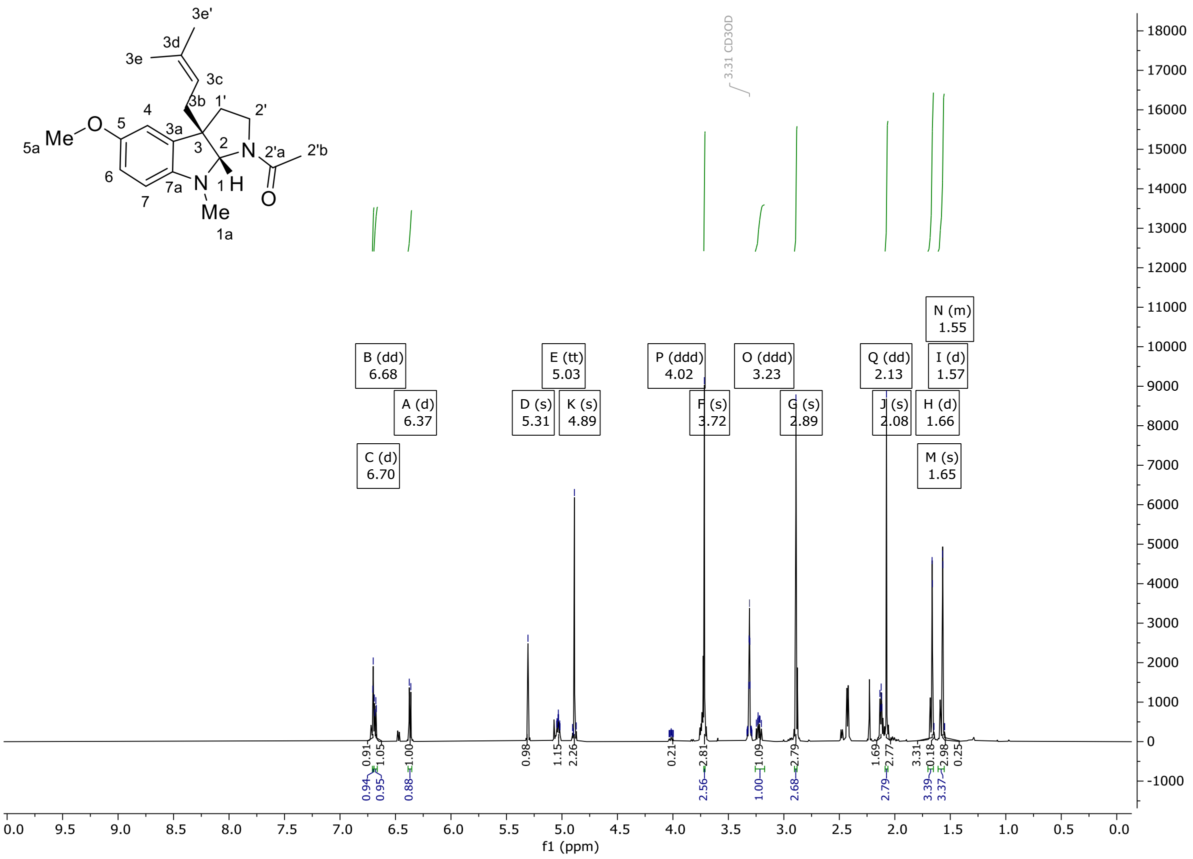

Figure S35: Crude 1H-NMR-spectrum (600 MHz) of S12 in CD3OD.

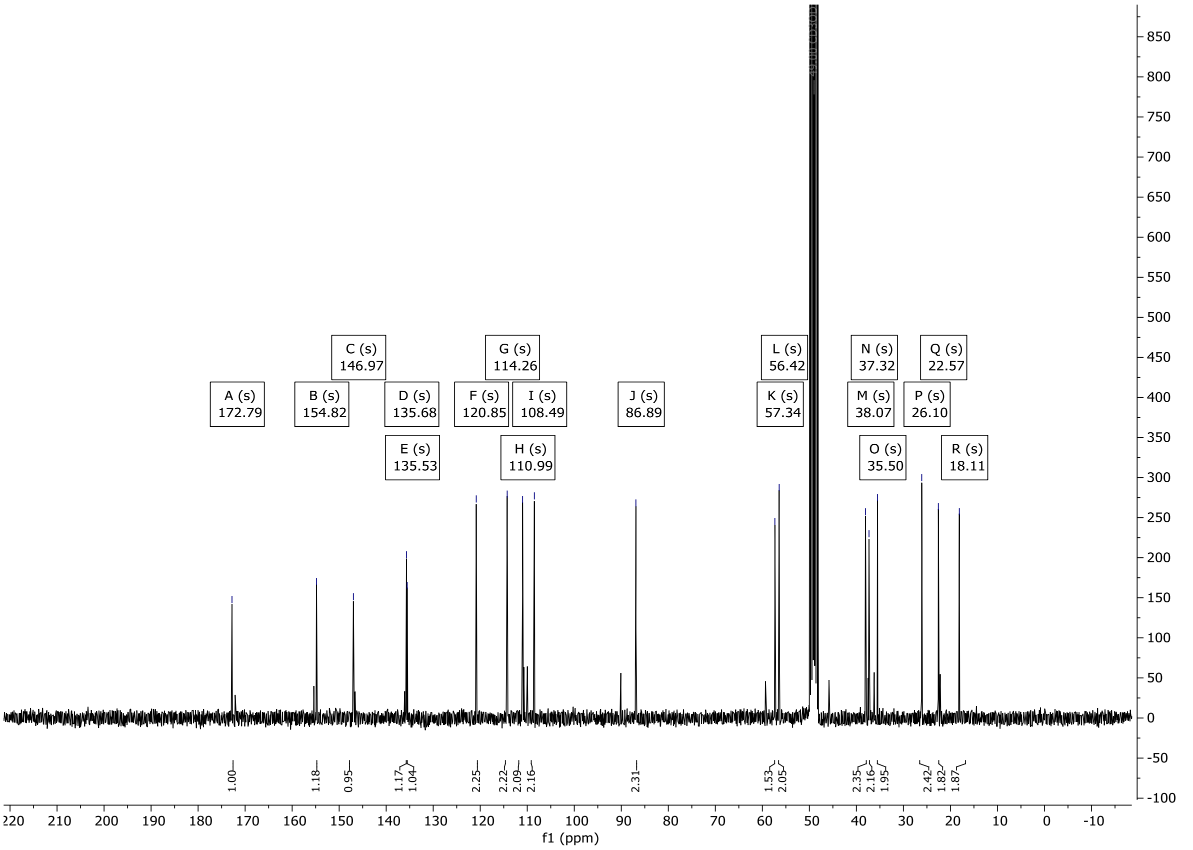

Figure S36: Crude 13C-NMR-spectrum (151 MHz) of S12 in CD3OD.

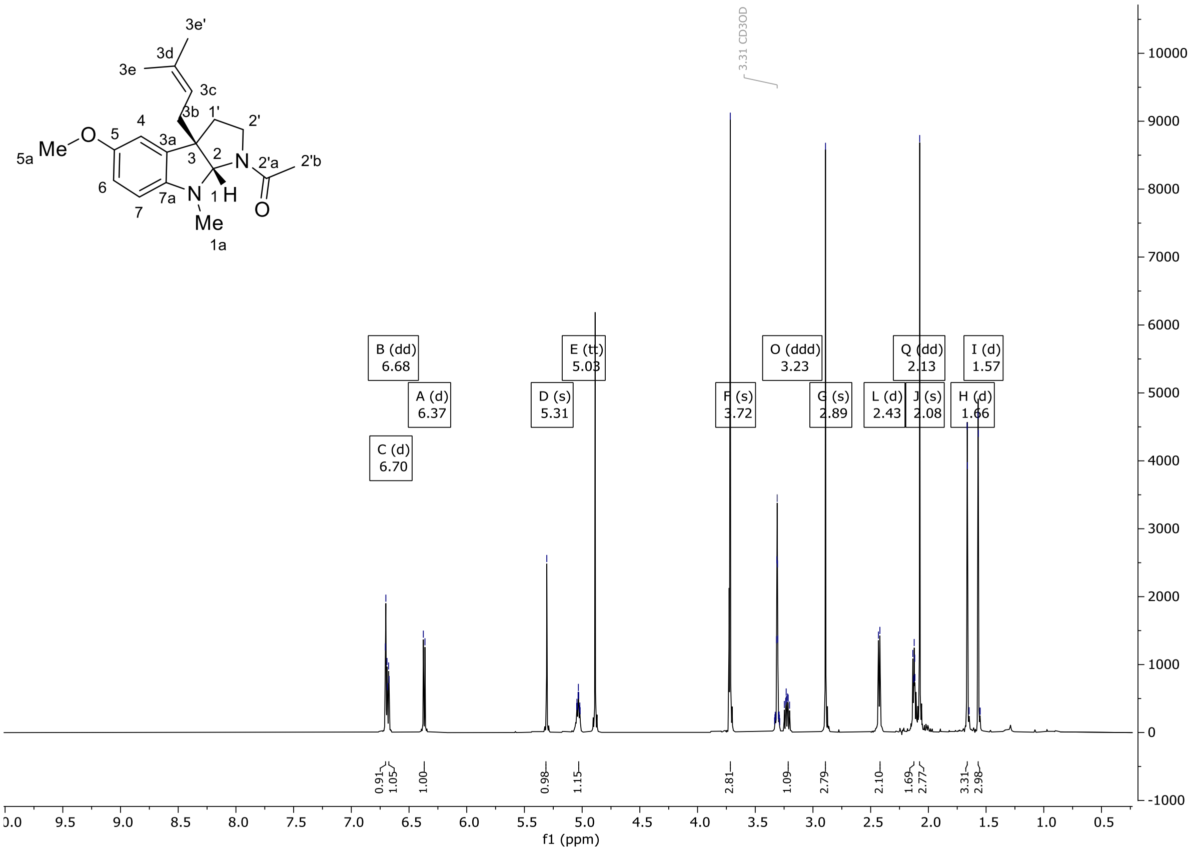

Figure S37: Synthethic ^{1}H-NMR-spectrum (600 MHz) of **S12** in CD_3OD.

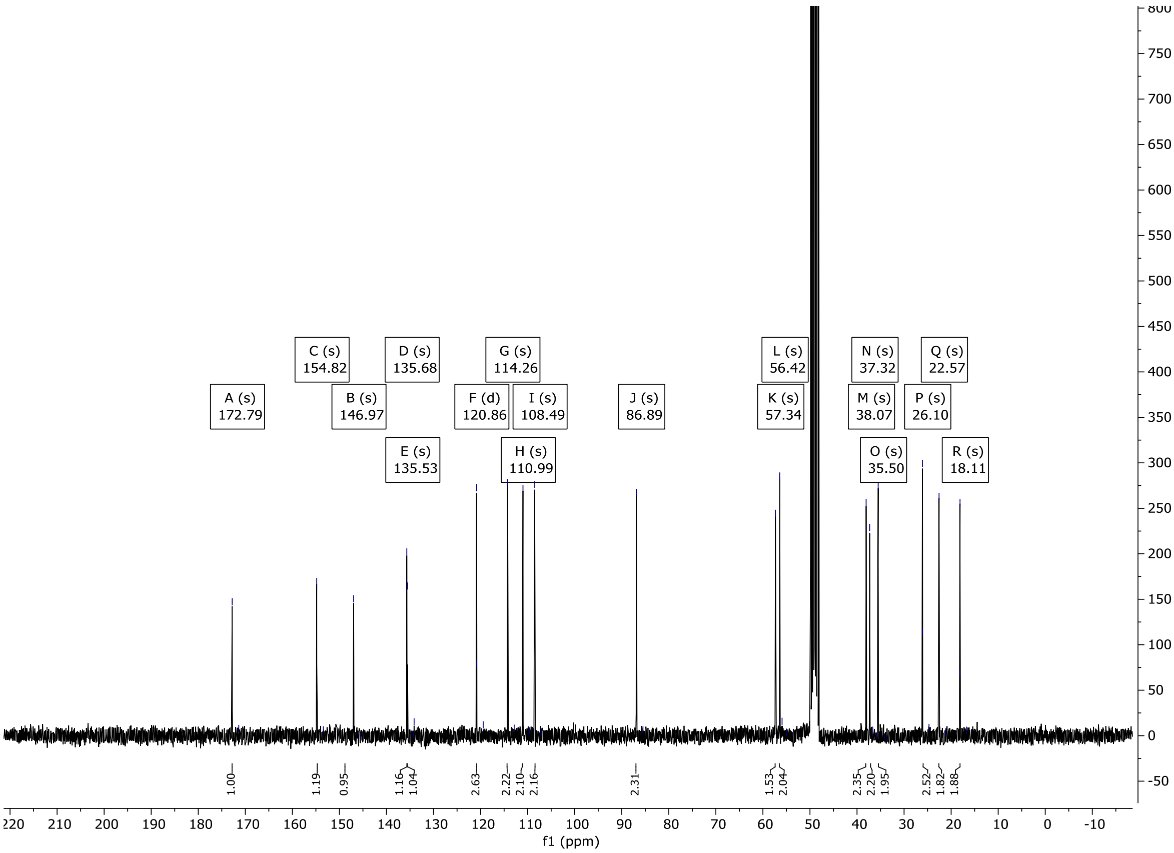

Figure S38: Synthethic ^{13}C-NMR-spectrum (151 MHz) of **S12** in CD_3OD.

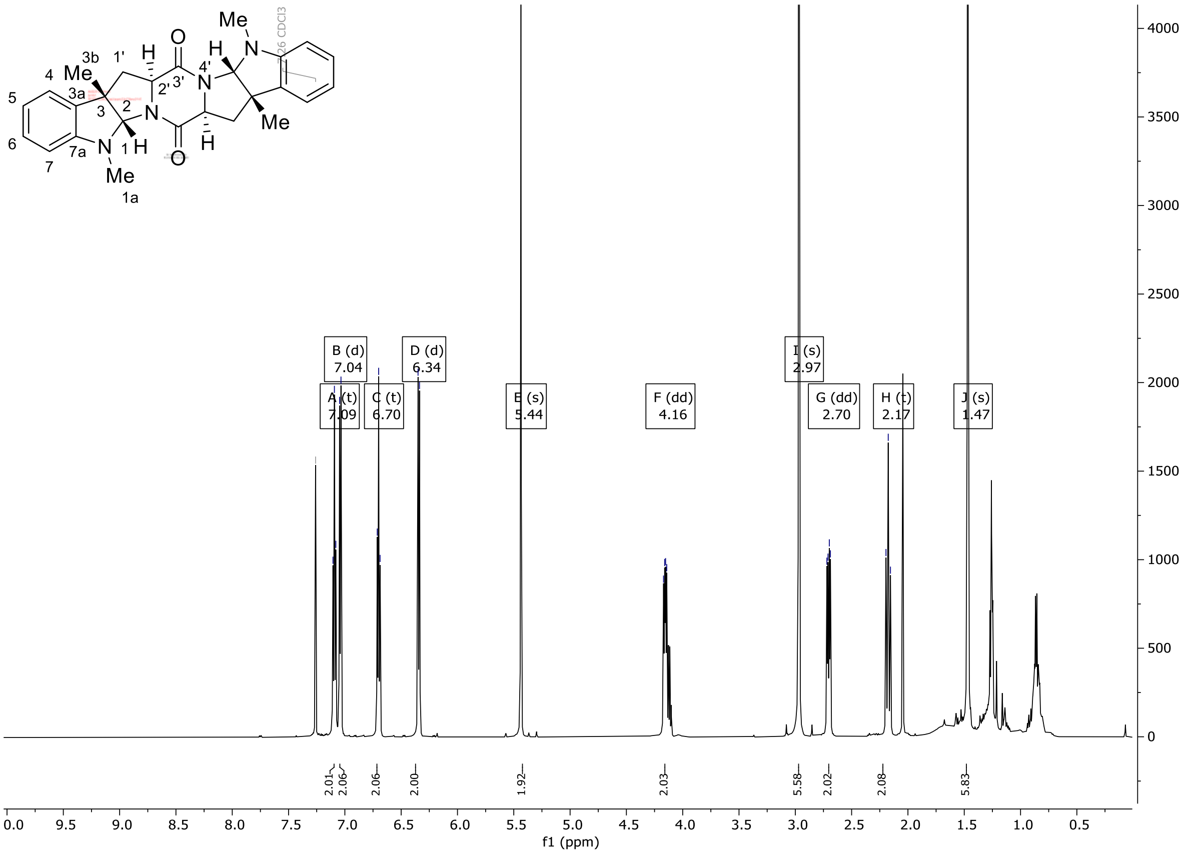

Figure S39: 1H-NMR-spectrum (600 MHz) of **S14** in $CDCl_3$.

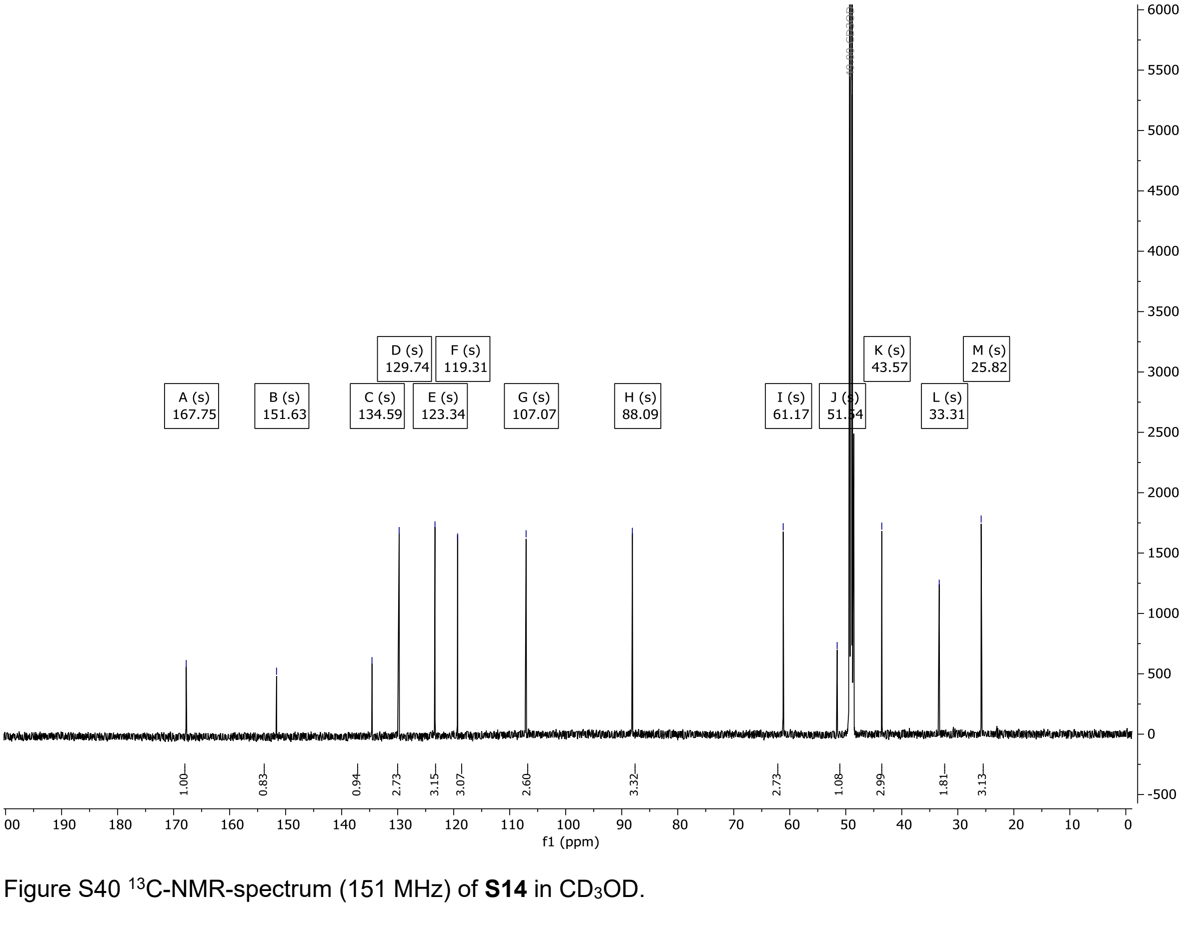

Figure S40 ^{13}C-NMR-spectrum (151 MHz) of **S14** in CD_3OD.

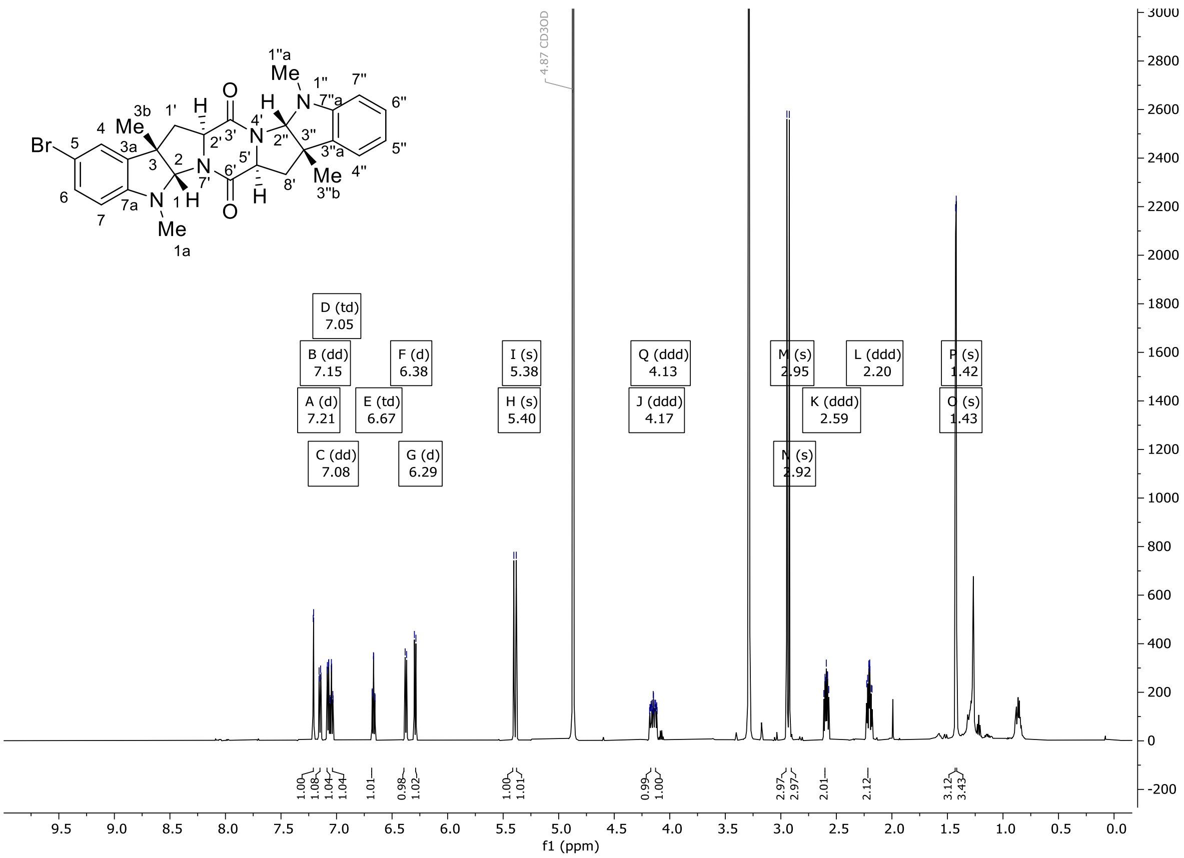

Figure S41: ^{1}H-NMR-spectrum (600 MHz) of **S15** in CD_3OD.

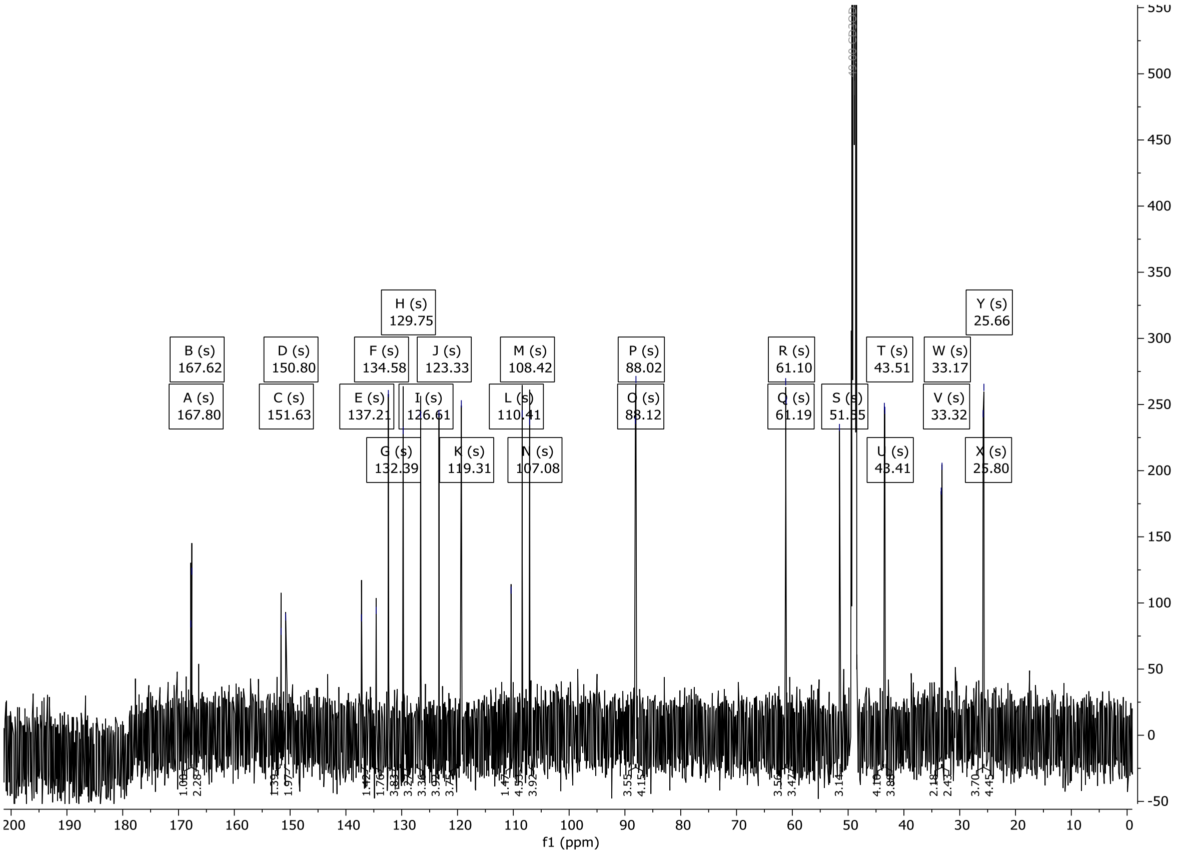

Figure S42: ^{13}C-NMR-spectrum (151 MHz) of **S15** in CD_3OD.

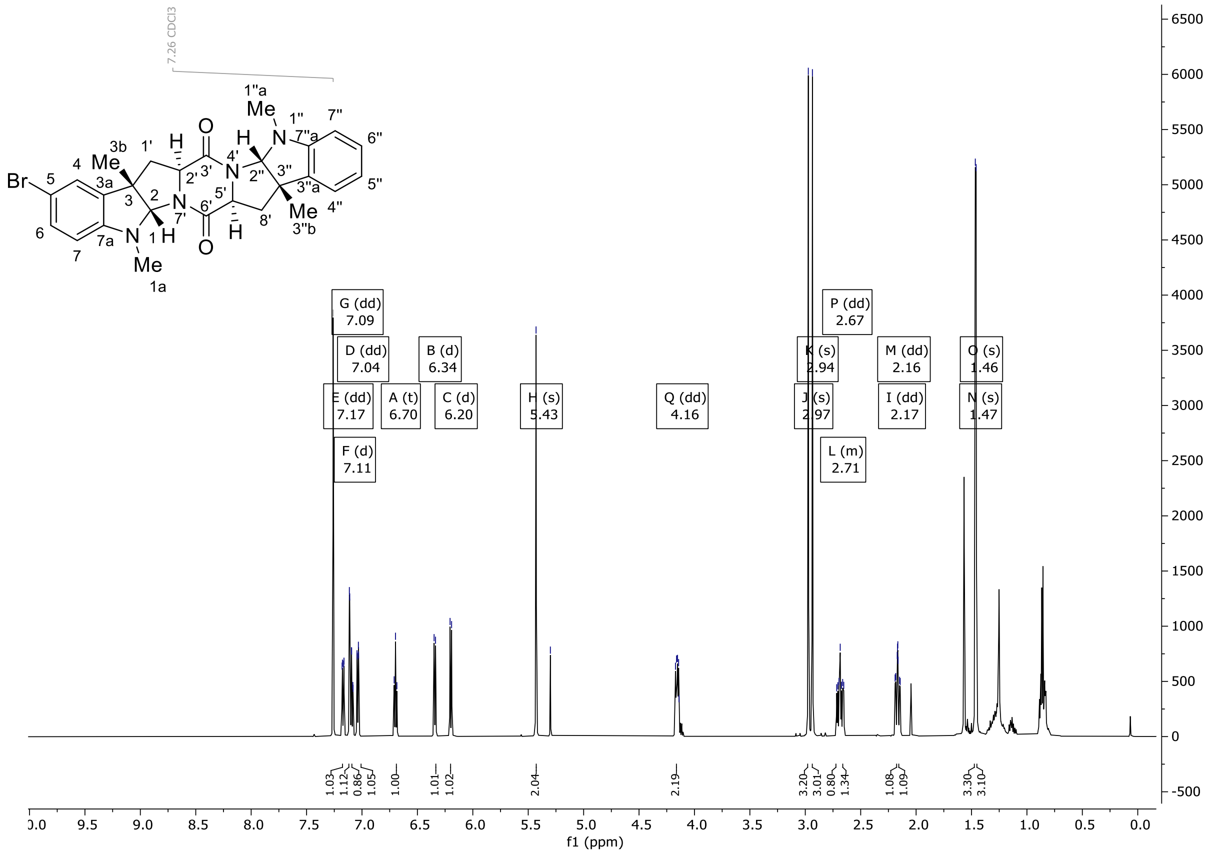

Figure S43: ^{1}H-NMR-spectrum (600MHz) of **S15** in $CDCl_3$.

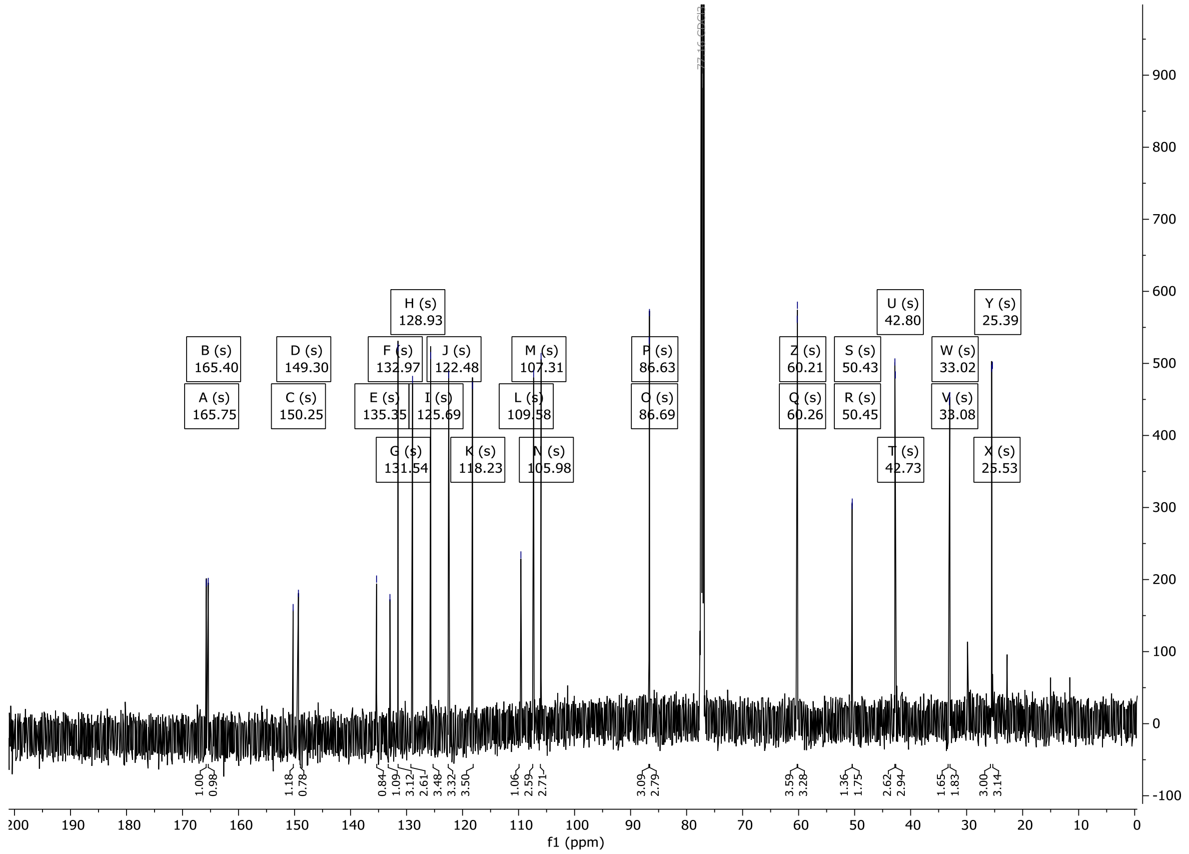

Figure S44: ^{13}C-NMR-spectrum (151 MHz) of **S15** in $CDCl_3$.

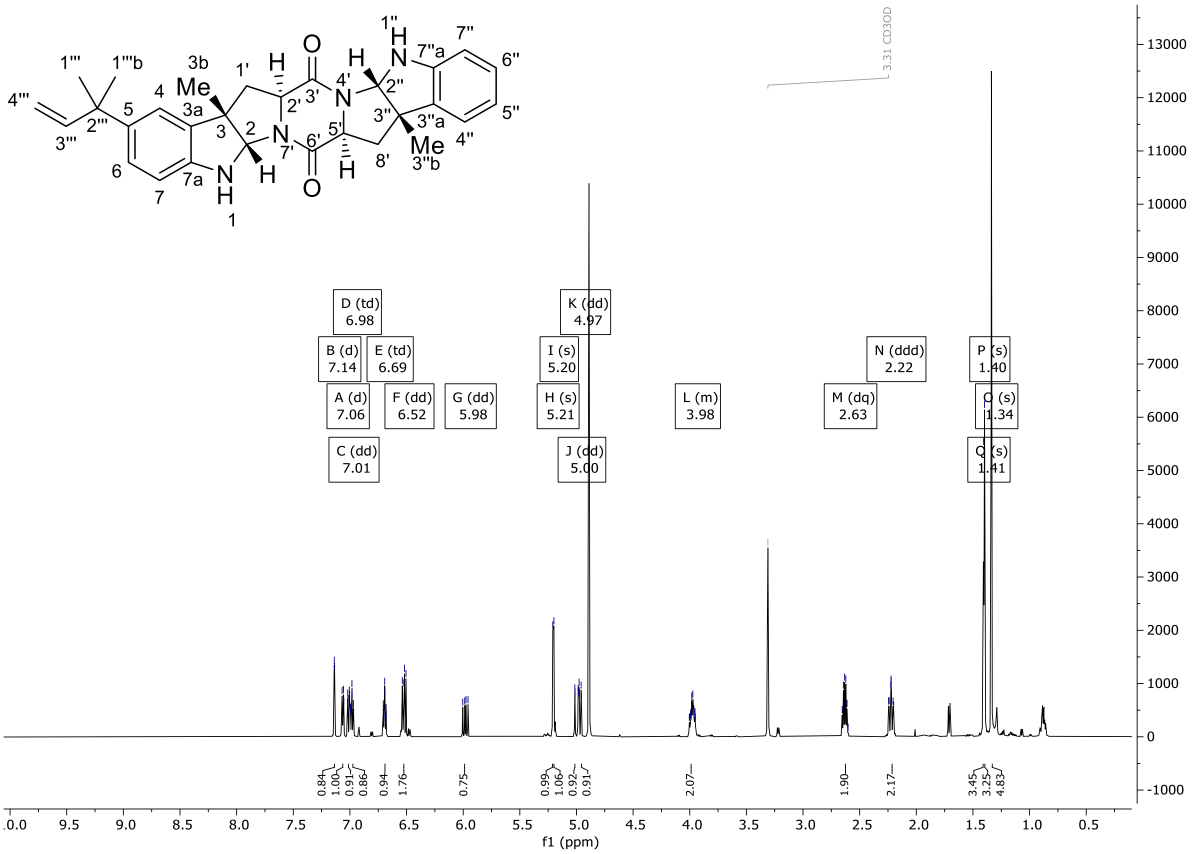

Figure S45: ^{1}H-NMR-spectrum (600 MHz) of **19** in CD_3OD.

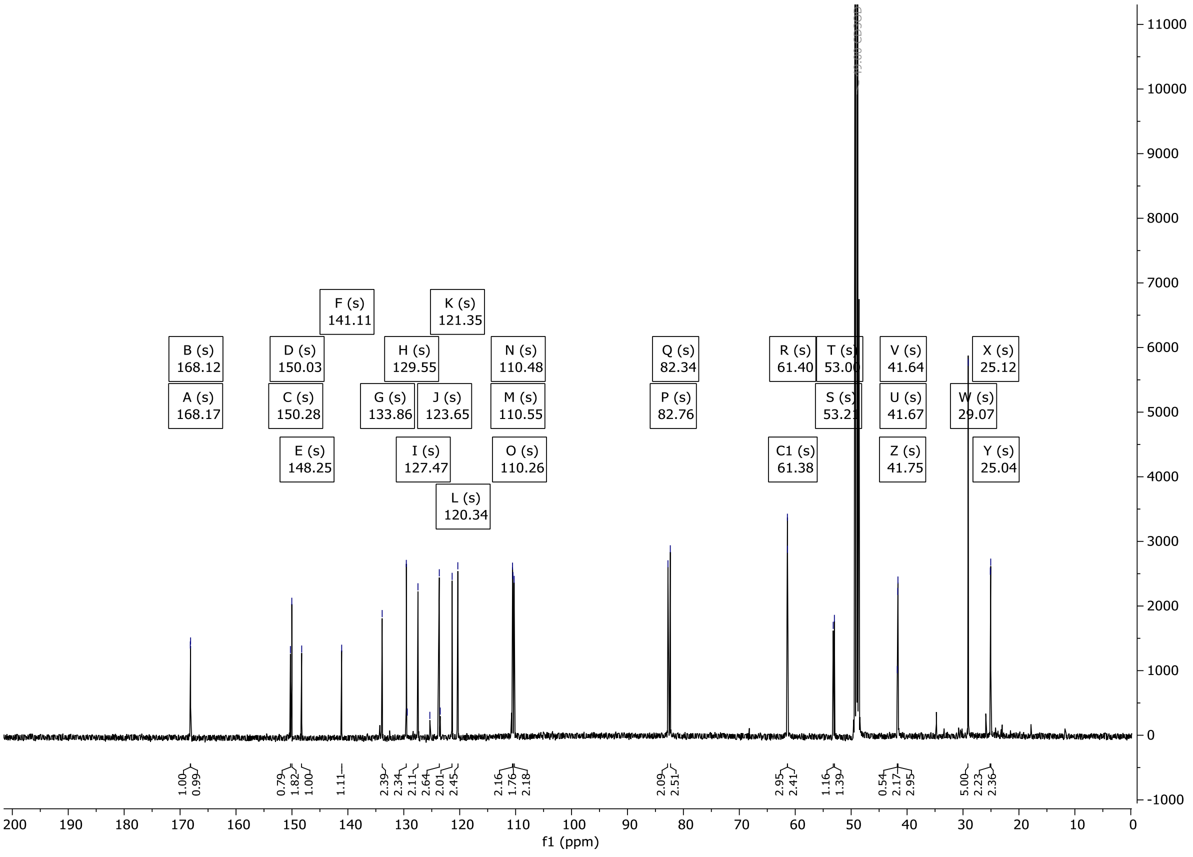

Figure S46: ^{13}C-NMR-spectrum (151 MHz) of **19** in CD_3OD.

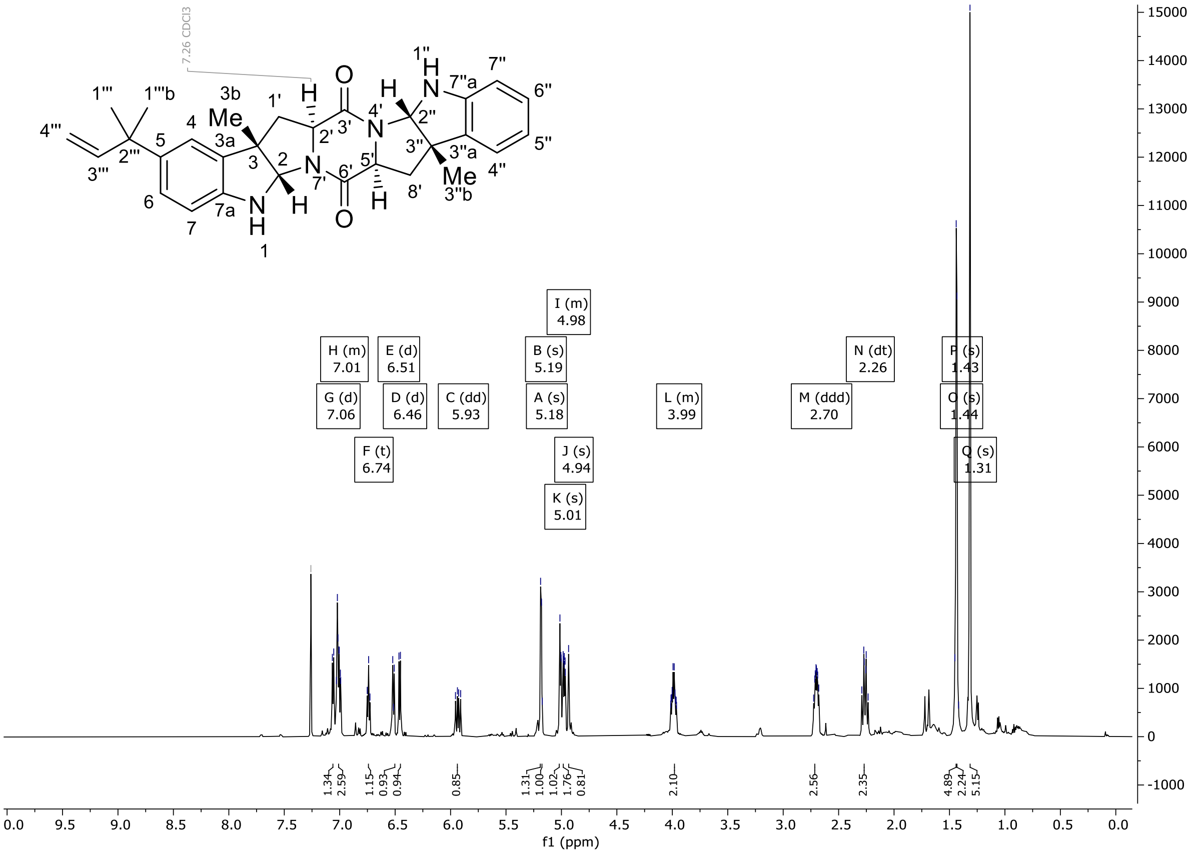

Figure S47: ^{1}H-NMR-spectrum (600 MHz) of **19** in $CDCl_3$.

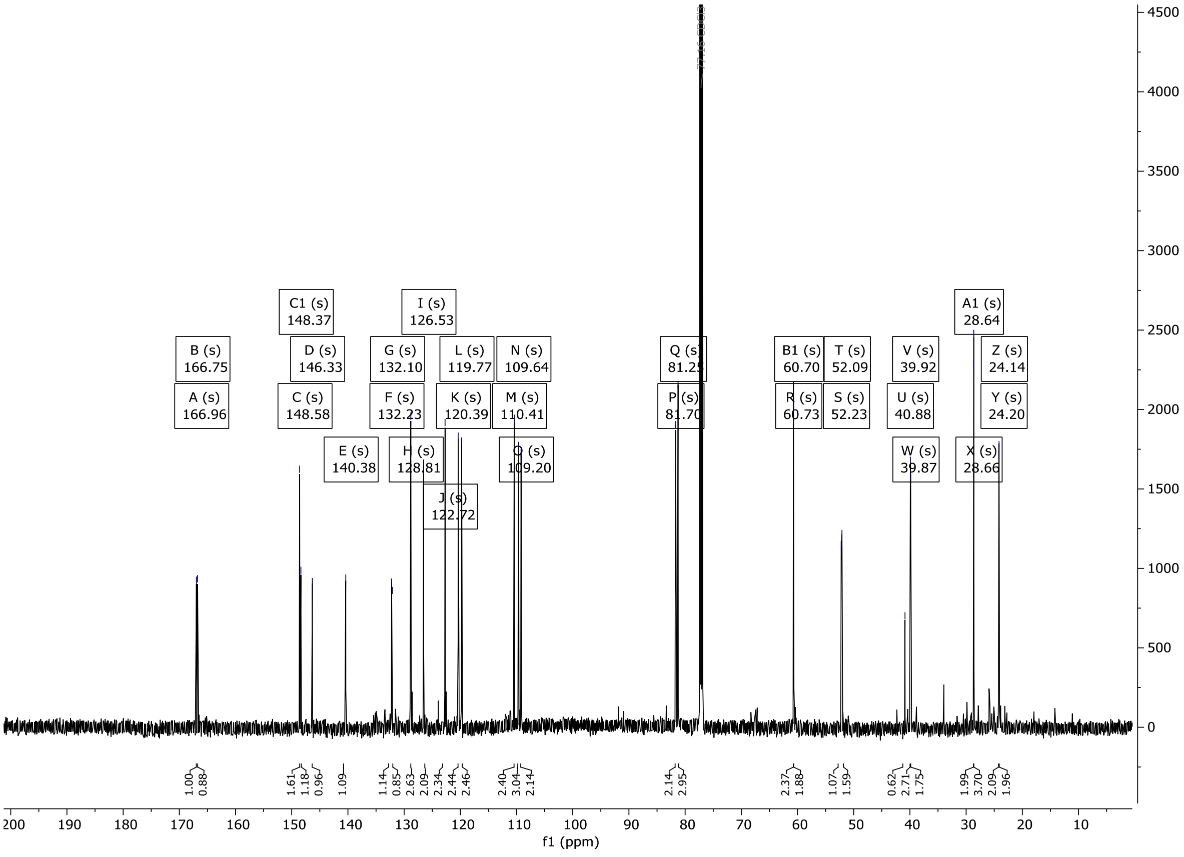

Figure S48: ^{13}C-NMR-spectrum (151 MHz) of **19** in $CDCl_3$.

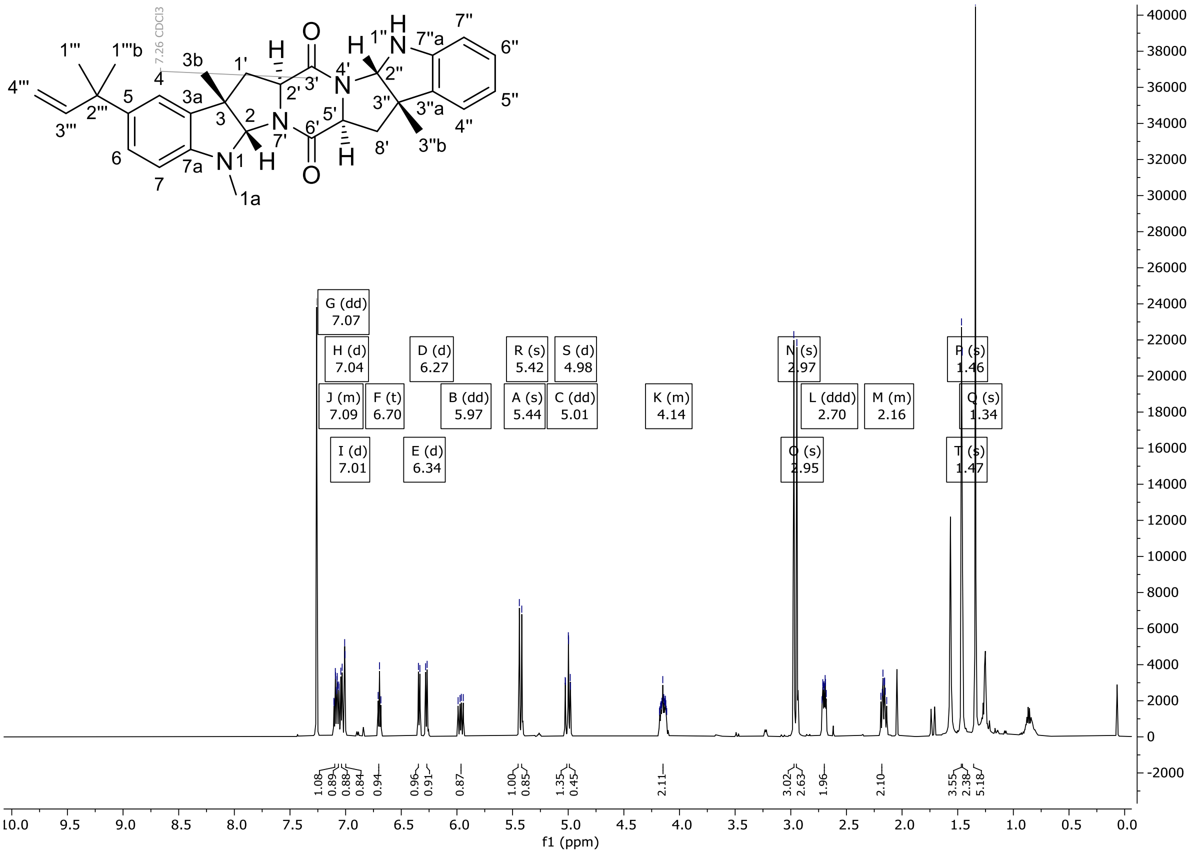

Figure S49 ^{1}H-NMR-spectrum (600 MHz) of lansai B (**3**) in $CDCl_3$.

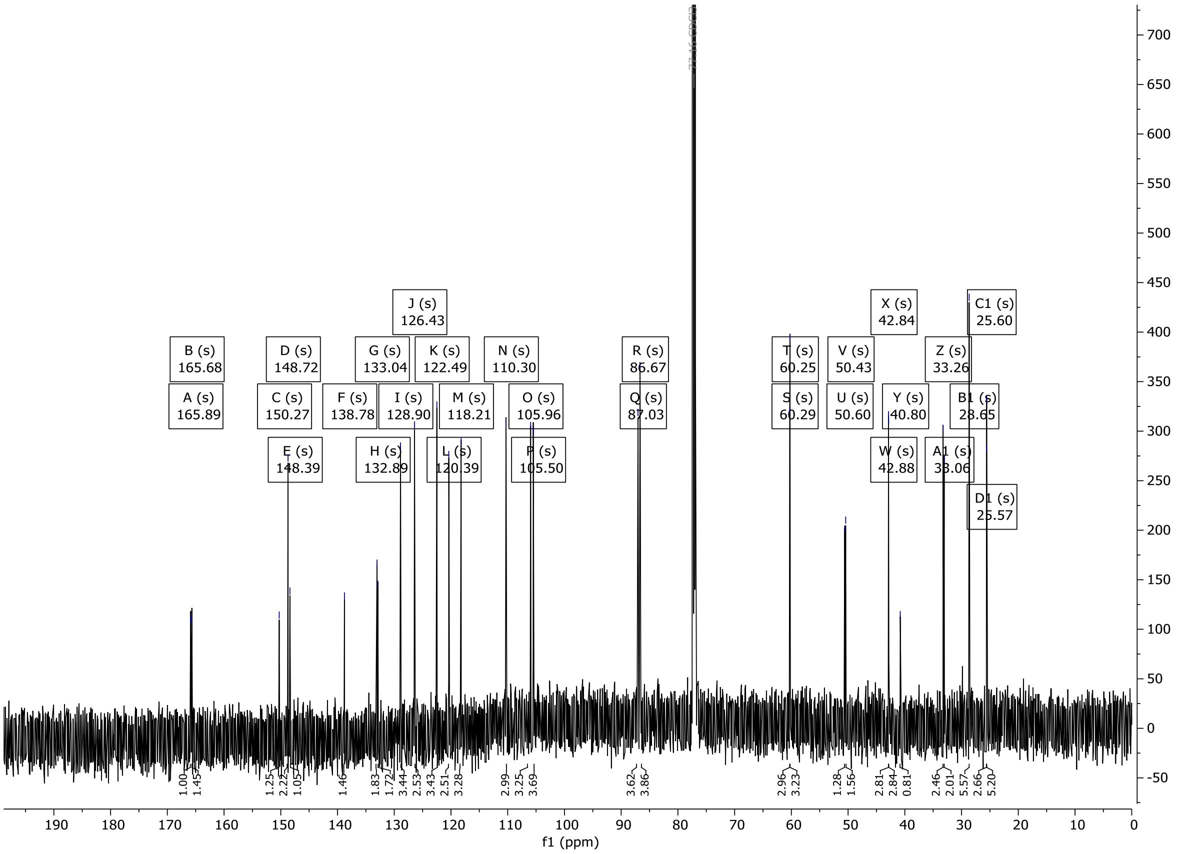

Figure S50: ^{13}C-NMR-spectrum (151MHz) of lansai B (**3**) in $CDCl_3$.

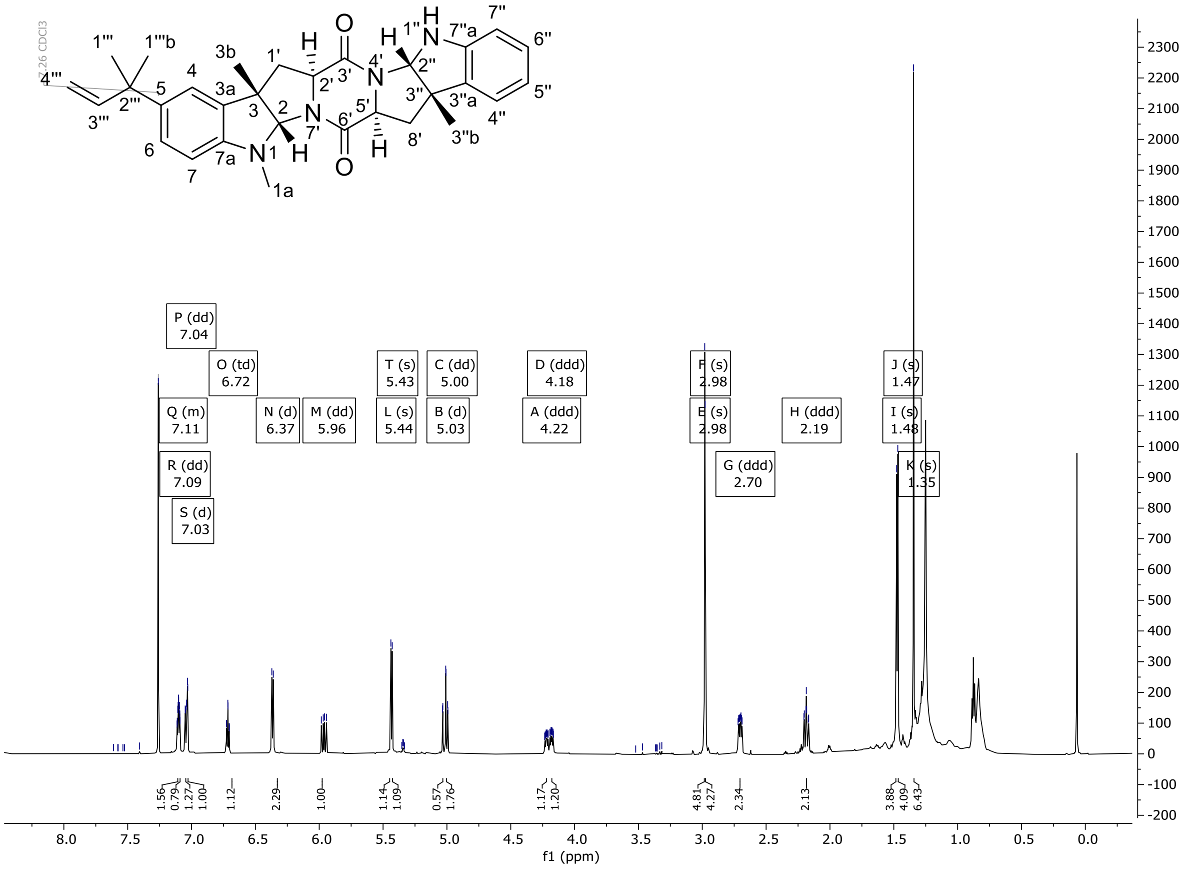

Figure S51: ^{1}H-NMR-spectrum (700 MHz) of lansai B (**3**) in $CDCl_3$.

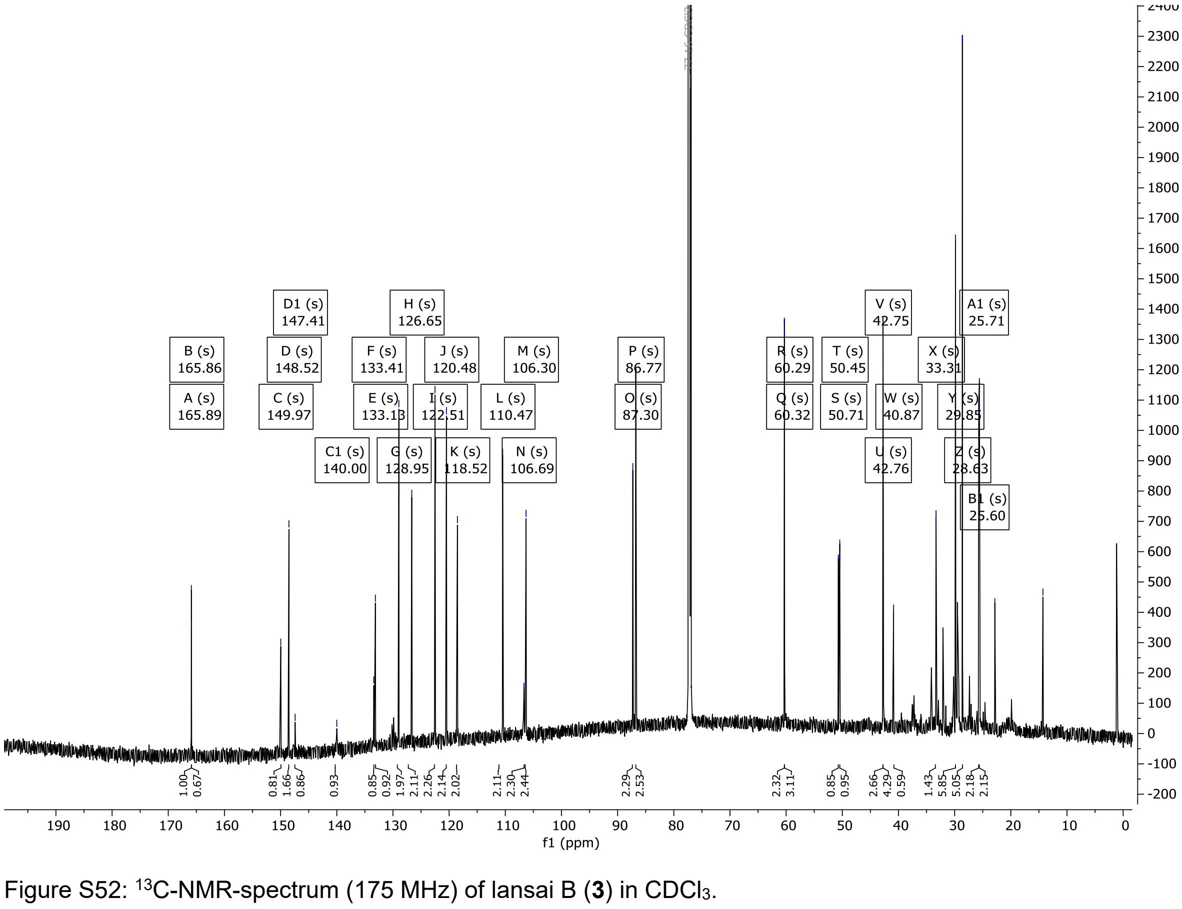

Figure S52: ^{13}C-NMR-spectrum (175 MHz) of lansai B (**3**) in $CDCl_3$.

4.3. References

[7] P. Tuntiwachwuttikul, T. Taechowisan, A. Wanbanjob, S. Thadaniti, W. C. Taylor, *Tetrahedron* **2008**, *64*, 7583-7586.

[14] H. Wang, S. E. Reisman, Angew. Chem. 2014, 126, 6320-6324, *Angew. Chem., Int. Ed.* **2014**, *53*, 6206-6210.

[24] M. Haase, B. David, B. Paschold, T. Classen, P. Schneider, N. Pozhydaieva, H. Gohlke, J. Pietruszka, *ACS Catalysis* **2024**, *14*, 227-236.

[26] M. Haase, O. H. Weiergraeber, B. David, E. Pfirmann, B. Paschold, H. Gohlke, J. Pietruszka, *Chem. Sci.* **2025**, *16*, 4519-4527.

[30] D. K. Romney, J. Murciano-Calles, J. E. Wehrmuller, F. H. Arnold, *J. Am. Chem. Soc.* **2017**, *139*, 10769-10776.

[31] M. Dick, N. S. Sarai, M. W. Martynowycz, T. Gonen, F. H. Arnold, *J. Am. Chem. Soc.* **2019**, *141*, 19817-19822.

[34] Y. Yang, S. L. Buchwald, *J. Am. Chem. Soc.* **2013**, *135*, 10642-10645.

[40] L. Mao, R. Bertermann, S. G. Rachor, K. J. Szabó, T. B. Marder, *Org. Lett.* **2017**, *19*, 6590-6593.

[41] T. M. Rosch, J. Tenhaef, T. Stoltmann, T. Redeker, D. Kösters, N. Hollmann, K. Krumbach, W. Wiechert, M. Bott, S. Matamouros, J. Marienhagen, S. Noack, *ACS Synth. Biol.* **2024**, *13*, 2227-2237.

[42] G. Blaser, J. M. Sanderson, A. S. Batsanov, J. A. K. Howard, *Tetrahedron Lett.* **2008**, *49*, 2795-2798.

[43] A. A. Fayad, C. Pubill-Ulldemolins, S. V. Sharma, D. Day, R. J. M. Goss, *Eur. J. Org. Chem.* **2015**, 5603-5609.
